# Expanding the scope of methylation-sensitive restriction enzyme (MSRE) PCR for forensic identification of body fluids through the novel use of methylation-dependent restriction enzymes (MDRE) and the combination of autosomal and Y-chromosomal markers

**DOI:** 10.1007/s00414-023-03097-9

**Published:** 2023-10-24

**Authors:** Jessica Rothe, Jessica Maria Becker, Maral Charchinezhadamouei, Sophia Mähr, Felizitas Lembeck, Nora Dannemann, Marion Nagy

**Affiliations:** Department of Forensic Genetics, Institute of Legal Medicine and Forensic Sciences, Charité-Universitätsmedizin Berlin, Augustenburger Platz 1, 13353 Berlin, Germany

**Keywords:** MSRE PCR, Body fluid identification, CpG sites

## Abstract

Methylation-sensitive/-dependent restriction enzyme (MSRE/MDRE) PCR can be performed to detect hypomethylated or hypermethylated CpG sites. With the combined use of different tissue-specific CpG markers, MSRE/MDRE-PCR leads to tissue-specific methylation patterns (TSMPs), enabling the correlation of DNA samples to their source tissue. MSRE/MDRE assays can use the same platform as forensic STR typing and offer many advantages in the field of forensic body fluid detection. In the present study, we aimed to establish MSRE assays for the detection of blood, saliva, vaginal secretion, and semen, using markers from literature and from our own database search. We designed two different MSRE test-sets, which include two novel Y-chromosomal non-semen markers, and enable differentiation between female and male non-semen samples. Furthermore, we established an MSRE/MDRE semen approach, which includes only Y-chromosomal non-semen and semen markers. This Y-semen multiplex PCR utilizes the novel combination of the methylation-sensitive enzyme *Sma*I and the methylation-dependent enzyme *Gla*I, which enables more sensitive detection of male body fluids within male/female DNA mixtures. Our validation tests confirmed that MSRE/MDRE assays exhibit high sensitivity, similar to that of STR typing.

**Supplementary Information:**

The online version contains supplementary material available at 10.1007/s00414-023-03097-9.

## Introduction

Contextualization of biological traces through correlation to a body fluid (bf) for example, saliva, blood, semen, or vaginal secretion provides valuable information for the reconstruction of events and activities related to a crime. Multiple different techniques can be used for this purpose, including chemical sprays, microscopy, immunologic assays, or RNA-based analysis [1]. Another promising marker type to identify the bf from a biological trace are DNA methylation sites because of their high stability and discrimination power.

DNA methylations mainly appear at position 5 of a cytosine next to a guanine (termed a CpG site) and are a basic mechanism of epigenetic regulation of the human genome. There are about 28 million CpG sites in the human genome, which have been shown to regulate gene expression and thus play important roles in the formation of different tissues or cell types. CpG sites are commonly grouped into CpG islands (CGI). The largest class of CGIs comprises hypomethylated CpG sites in promoter regions that are responsible for polymerase II–based transcription initiation in 70% of genes. However, other classes of CGIs are only within the vicinity of genes, or even in gene deserts, which are still involved in gene regulation and tissue specificities [2].

Previous studies have demonstrated the use of CpG sites for body fluid identification. However, most of these studies have used bisulfite-based assays, which are not suitable for forensic case work because they require high amounts of DNA (~ 10 ng) [3, 4]. To overcome this issue, Frumkin et al. [5] and Wasserstrom et al. [6] introduced a methylation-sensitive restriction enzyme PCR (MSRE-PCR) assay for the forensic detection of bf, which requires a much lower amount of DNA than bisulfite-based approaches. MSRE uses tissue-specific methylated or non-methylated CpG sites, which lay in a restriction site for a methylation-sensitive restriction enzyme, such as *Hha*I. Before PCR, a digestion step results in cleavage of the DNA sequence with the unmethylated CpG site. Then, the DNA sequence with the methylated CpG is amplified. The combination of several bf-specific markers in one multiplex MSRE-PCR allows identification of bf type based on the obtained tissue-specific methylation pattern (TSMP).

Although MSRE-PCR offers several advantages, little research has been performed to test this method. Notably, Lin and colleagues have discovered new markers for MSRE-PCR, and have impressively demonstrated the simultaneous amplification of MSRE bf and STR markers [7, 8]. To date, only a few markers for MSRE-PCR have been identified. One way to find tissue-specific methylation markers is to search for tissue-specific expressed genes. However, this strategy is more suitable for RNA- or protein-based methods and is less efficient for MSRE-PCR. Tissue-specific expressed genes generally occur within hypomethylated CGI, which would result in complete digestion in MSRE, and thus yield no signal. For MSRE-PCR, suitable bf-specific CpG sites may be identified in methylation databases, such as the Infinium HumanMethylation450 beadarray (450K array). The 450K array contains 485,577 CpG sites of the human genome, covering 99% of the known RefSeq genes [9].

In our present study, we tried to build our own MSRE multiplex test-sets for the identification of bf, including saliva, blood, semen, and vaginal secretion. We tested methylation bf markers that had been identified in previous studies and also aimed to find new markers within NCBI datasets from the 450K array. Furthermore, we established the first MSRE-PCR multiplex PCR with Y-chromosome bf markers. Y-linked bf markers can correlate the bf to a male or female identity and thereby help to reveal complex female/male mixtures, as often appear in casework, especially in sexual assaults. We demonstrate that the design possibilities for MSRE-PCR can be increased by using other restriction enzymes, which are not only methylation-sensitive but also methylation-dependent. We introduce the first application for the forensic identification of bf, which uses both a methylation-sensitive and a methylation-dependent restriction enzyme (MDRE) in a single PCR. We validated our multiplex MSRE/MDRE PCR for specificity and sensitivity, with different male/female mixtures, as well as for stability by testing long-term stored samples.

## Material and methods

### Sample collection and preparation

Body fluids were collected from 59 volunteers. All volunteers signed an informed consent after reading study information. This study (EA4 189 21) was approved by the Medical Ethics Committee of the University Hospital Charité in Berlin.

Vaginal secretion and menstrual blood were collected from the volunteers using sterile cotton swabs. Buccal mucosa was collected by oral scraping, also with sterile cotton swabs. Saliva and semen were first collected in a Falcon tube, and then, a cotton swab was briefly immersed in the bf and transferred into a fresh 2-mL extraction tube by the volunteers. Peripheral blood was collected from finger tips using a lancet and sterile cotton swabs. We collected a total of 11 semen, 52 buccal mucosa, 50 saliva, 45 blood, 7 menstrual blood, and 17 vaginal secretion samples. Furthermore, we also tested 38 blood, 10 semen DNA samples, and 1 saliva DNA sample stored from the German interlaboratory GEDNAP tests, which had been processed in the years 2014 to 2020 (Online Resource 1a).

From our collected samples, DNA was extracted using the EZ1®DNA Blood 200 µL kit with the 6GC Magtration System M6 robot (Qiagen, Hilden, Germany), following the manufacturer’s instructions but with the use of ATL lysis buffer. From the GEDNAP samples, DNA was extracted using the Investigator Kit, with the ATL lysis buffer, and the QiaSymphony robot (Qiagen). The applied extraction protocols are the same protocols used in routine forensic casework. DNA quantification was conducted using the Quantus™ Fluorometer and the QuantiFluor® ONE dsDNA Kit (Promega, Mannheim, Germany), following the manufacturer’s instructions.

### Search and selection of autosomal and Y-chromosomal CpG loci

For our loci search, we applied two different strategies. First, we looked for previously published bf markers from MSRE-PCR or bisulfite-based assays. Second, we extended our search by using the NCBI database of the Infinium HumanMethylation450 BeadChip (platform GPL13534). Our loci search, which was particularly focused on blood and vaginal secretion markers, yielded 103 potential CpG markers from the database search and 40 from the literature (more details of loci search are described in Online Resource 1b and c, Online Resource 2). Promising markers were selected for pre-testing, mostly by gel electrophoresis. In some cases, we tested markers from the literature directly by capillary electrophoresis, particularly the markers reported by Wasserstrom et al. [6] and Lin et al. [7].

### MSRE/MDRE-PCR

MSRE/MDRE-PCR was conducted in a 10-µL volume, using 1 × Multiplex Mastermix (Qiagen, Hilden, Germany), 12% Detection Enhancer (Applied Biosystems), with or without 0.5 µL of the restriction enzyme *Hha*I, *Sma*I (Thermo Fischer Scientific, Vilnius, Lithuania), or *Gla*I (SibEnzyme, Ludwigshafen, Germany), and with 0.1–0.6-µM primer (TIB Molbiol, Berlin, Germany) (Online Resource 2). PCR was performed in a Thermocycler Dyad (BioRad) or Thermocycler Mastercycler nexus (Eppendorf). The cycling protocol was adopted from Lin et al. as follows: digestion at 37 °C for 15 min (*Hha*I) or 30 °C for 15 min (*Sma*I/*Gla*I); inactivation and denaturation at 95 °C for 11 min; 30 cycles of 94 °C for 20 s, 59 °C for 2 min, and 72 °C for 1 min; and a final extension step of 60 °C for 45 min [7]. PCR products were analyzed using capillary electrophoresis (CE) on a 3500 Genetic Analyzer (Applied Biosystems, Darmstadt Germany). For a preliminary check of newly designed markers, MSRE-PCR was carried out with four additional cycles using unlabeled primers. The product was checked by gel electrophoresis (GE) (Online Resource 2). Pre-tests were conducted using only one male and one female sample for each bf.

### Analysis of the tissue-specific methylation patterns

All TSMPs were analyzed using Genemapper ID X1.4 software (Applied Biosystems) with a 50 RFU threshold. From each multiplex, the single markers of the TSMPs were assessed for a successful or non-successful digest in order to determine their specificity for each tested bf. Therefore, each multiplex contained a PCR control (PC) with no cutting site and a digestion control (DC) with at least one cutting site. Body fluid markers were interpreted as non-digested when they showed higher peak heights than the DC. To generate a representative digestion score, we calculated the ratios of the single markers to the digestion control: ratio = peak height of marker/peak height of digestion control. When the digestion control was ~ 5000 RFU or higher, the analyses were repeated with reduced DNA input for complete digestion. For the Y-chromosomal semen multiplex (Y-semen test-set), ratios were calculated by using either the non-semen marker or the semen marker (depending on the bf) as the digestion control (DC). This was due to the abundance of representative digestion control in the Y-semen test-set, because the intended DC always showed complete digestion, such that the digestion score would have resulted in extremely high ratios. R-Studio was used to plot the TSMPs as a Boxplot and Heatmap for displaying marker specificity and PCR efficiency. Markers were counted as “false-positives” when the ratio (digestion score) exceeded “2” for non-intended markers. Markers were counted as “false-negatives” when the ratio (digestion score) was lower than “2” for intended markers.

## Results

### Loci search and pre-testing

Our loci search from the literature and the NCBI database yielded the identification of 143 loci, from which we selected 49 (16 from literature and 33 from own database search) for further pre-tests (Table 1, Online Resource 2). In our literature search, we especially focused on the markers reported in MSRE-based investigations from Lin et al. [7] and Wasserstrom et al. [6], because these markers have been shown to exhibit bf specificity after *Hha*I digestion [13]. Among the markers reported by Wasserstrom et al., all tested semen markers (L6 and L7) and non-semen markers (L3 and L4), as well as the digestion control (DC), were confirmed as specific for their intended bf. However, among the tested markers reported by Lin et al. including SE-I (cg05261336) and SE-II (cg07485723) for semen, BL-I (cg04011671) and BL-II (cg18454288) for blood, SA-I (cg09652652) and SA-II (cg09107912) for saliva, and VG(cg15402210) for vaginal secretion we could only confirm SE-I and SA-I as bf-specific. In gel electrophoresis, SE-II also showed weak signals for buccal mucosa and vaginal secretion, and thus, we did no further testing with capillary electrophoresis (Table 1, Online Resource 2). In capillary electrophoresis the markers BL-I, BL-II, and SA-II were always digested by *Hha*I treatment. We examined their sequence information and found that BL-I and SA-II were designed with four *Hha*I cutting sites in the resulting PCR product. Therefore, we designed new variants for these markers with reduced numbers of *Hha*I cutting sites. Pre-testing revealed that the new variants BL-I_cut3 (two cutting sites) and SA-II_cut4 (one cutting site) were blood-specific and saliva-specific, respectively. The marker BL-II from Lin et al. harbored two closely localized cutting sites, which made it difficult to design a new variant with only one cutting site. For the marker VG, we did not test the primers designed by Lin et al. [7], but rather tested different variants with different numbers of cutting sites in gel and capillary electrophoresis. However, none of the tested variants showed specificity for vaginal secretion.

**Table 1:** Summary of loci search from all tested methylation sites

					Mean beta values for vaginal secretion, saliva, blood, and semen			
Approach	Ref	Intended body fluid specificity	Name in this study	Name in other studies	From Lin et al.	From this study	From other (see Ref.)	Conclusions
Bisulfite	[10]	Blood	BL_chr6_l	cg08792630	13%, 10%, **37%**, 5%	10%, 11%, **43%**, 8%	10%, 9%, **50%**, 8%	*Hha*I: not specific in GE, event. test in CE
	[10]	Saliva	SA-lV **SA-III**	cg26107890	6%, **33%,** 3%, 1%	8%, **51%**, 7%, 3%	8%, **53%**, 5%, 2%	*Hha*I: saliva specific, in SBV assay
	[10–12]	Vaginal	VG_chr7_l_A2 **VG-I**	VG-B/VF1 cg09765089	**40%**, 5%, 6%, 1%	**56%**, 5%, 12%, 5%	**35%**, 6%, 9%, 6%	*Hha*I: vaginal specific, in SBV assay
	[10–12]	Semen	Sperm_chr2_l **SE-III**	SE-B/SE1 cg17610929*	0,5%, 1%, 1%,** 98%**	5%, 7%, 8%,** 90%**	1%, 1%, 1%, **95%**	*Hha*I: semen specific, in auto/Y-semen assay
Diverse	[7, 12]	Semen	Sperm_chr7_SE_lB **SE-IV**	SE-I SE-A cg05261336*	1%, 1%, 1%, **95%**	1%, 2%, 2%,** 83%**		*Hha*I: semen specifc, in auto/Y-semen assay
	[8, 11]	Semen	Sperm_chr8_l + IB **SE-V**	SE2 cg26763284	4%, 11%, 5%, **97%**	10%, 7%, 7%, **92%**	2%, 2%, 2%, **90%**	*Hha*I: semen specific, in auto/Y-semen assay
	[7, 11]	Saliva	**SA-l**	SA-l/SA1 cg09652652	2%, **34%**, 2%, 1%	3%, **56%**, 4%, 2%	-	*Hha*I: saliva specific, in SBV assay
MSRE PCR (Endpoint)	[6, 13]	Non-semen	L4 **noSE-II**	L4 cg16363447	98%, 97%, 97%,** 4%**	97%, 96%, 97%, **30%**	-	*Hha*I: non-semen specific, in SBV and auto/Y-semen assay
	[6, 13]	Semen	L6 **SE-I**	L6 cg15639910	7%, 7%, 9%, **80%**	9%, 10%, 11%, **79%**	-	*Hha*I: semen specifc, in auto/Y-semen assay
	[6, 13]	Semen	L7 **SE-II**	L7 cg11721464	49%, 6%, 6%,** 89%**	7%, 9%, 10%, **85%**	-	*Hha*I: semen specifc, in auto/Y-semen assay
	[6, 13]	Non-semen	L3 **noSE-I**	L3 cg16617141	83%, 88%, 95%,** 10%**	82%, 84%, 95%, **34%**	-	*Hha*I: non-semen specific, in SBV and auto/Y-semen assay
	[7]	Saliva	SA-II_cut4 > **SA-II**	SA_II cg09107912*	3%, **22%**, 4%, 1%	6%, **34%**, 7%, 3%	-	*Hha*I: variant saliva specific, in SBV assay
	[7]	Vaginal	VG_chr2_l	VG cg15402210*	**29%,** 2%, 3%, 1%	**11%**, 5%, 7%, 3%	-	*Hha*I: not specific
	[7]	Blood	Bl_I_cut3 cg03717364* **BL-I**	BL I cg04011671	6%, 3%, **25%**, 1%	3%, 2%, **30%**, 2%	-	*Hha*I: variant blood specific, in SBV assay
	[7]	Blood	BL-II cg26163537*	BL-II cg18454288	4%, 4%, **16%**, 2%	6%, 7%, **18%**, 3%	-	*Hha*I: always digested
	[7]	Semen	Sperm_chr5_l	SE-II cg07485723	1%, 1%, 2%, **96%**	4%, 4%, 6%, **84%**	-	*Hha*I: not specific
	This study	Blood	BL_chr1_lll cg19637387	-	8%, 8%, **22%**, 6%	10%, 8%, **23%**, 8%	-	*Hha*I: blood specific, but bad PCR amplification
	This study	Blood	BL-chr2-l cg02345886*	-	28%, 17%, **50%**, 3%	18%, 17%, **53%**, 10%	-	HhaI: not specific
	This study	Blood	Bl_chr2_ll cg06645778	-	14%, 9%, **50%**, 3%	11%, 8%, **49%**, 7%	-	*Hha*I: not specific in GE, event. test in CE
	This study	Blood	BL_chr11_l cg04434593	-	7%, 8%, **22%,** 2%	9%, 9%, **23%**, 4%	-	*Hha*I: not specific
	This study	Blood	BL_chr12_l cg22249612	-	7%, 7%, **35%**, 1%	7%, 10%, **41%**, 2%	-	*Hha*I: non-semen marker, event. test in CE
	This study	Blood	BL_chr14_l cg23051349	-	8%, 4%, **27%**, 2%	5%, 4%, **29%,** 3%	-	*Hha*I: not specific
	This study	Blood	BL_chr16_l cg06329093	-	8%, 9%, **22%**, 5%	10%, 11%, **20%**, 7%	-	*Hha*I: not specific
	This study	Blood	BL_chr17_l cg05164926	-	7%, 6%, **29%**, 1%	6%, 5%, **22%**, 2%	-	*Hha*I: not specific
	This study	Blood	BL_chr22_l cg19343611	-	8%, 8%, **23%**, 1%	10%, 10%, **27%**, 3%	-	*Hha*I: not specific in GE, event. test in CE
	This study	Vaginal	VG_1_l cg13356427	-	60%, 10%, 2%, 2%	26%, 28%, 6%, 4%	-	*Hha*I: not specific
	This study	Vaginal	VG_1_ll cg09809932	-	60%, 4%, 10%, 1%	15%, 8%, 15%, 7%	-	*Hha*I: not specific in GE, event. test in CE
	This study	Vaginal	VG_chr5_l cg04541368	-	**66%**, 8%, 3%, 1%	**32%**, 11%, 8%, 3%	-	*Hha*I: not specific in GE, event. test in CE
	This study	Vaginal	VG_7_ll (cg18502142 cg09803262) **VG-II**	-	**75%,** 28%, 14%, 2%	**55%**, 38%, 23%, 6%	-	*Hha*I: non-semen non-blood specific, in SBV assay
	This study	Vaginal	VG_chr10_l cg26439963	-	60%, 3%, 4%, 3%	7%, 7%, 6%, 9%	-	*Hha*I: not specific
	This study	Vaginal	VG_chr14_l cg09197895*	-	30%, 1%, 1%, 0,4%	2%, 1%, 1%, 1%	-	*Hha*I: not specific
	This study	Vaginal	VG_17_l cg03656099	-	**52%**, 4%, 3%, 1%	**24%,** 4%, 7%, 2%	-	*Hha*I: not specific in GE, event. test in CE
	This study	Vaginal	VG_chr22_l cg14703829	-	**61%**, 13%, 12%, 5%	**23%**, 13%, 16%, 11%	-	*Hha*I: not specific in GE, event. test in CE
	This study	Blood	BL-chrY-l cg03416979*	-	-, 17%, **92%**, 5%	-, 65%, **87%**, 20%	-	*Hha*I: not specific
	This study	Blood	BL-chrY-II cg14029254*	-	-, 12%, **89%**, 3%	-, 68%, **87%**, 19%	-	*Hha*I: non-semen specific
	This study	Blood	BL_Y_lll cg08265308	-	-, 4%, **91**%, 2%	-,7%, **90%**, 14%	-	*Hha*I: non-semen specific
	This study	Blood	BL_chrY_lV cg10363397	-	-, 13%, **94%**, 1%	-, 55%, **96%**, 13%	-	*Hha*I: non-semen specific
	This study	Blood	BL_chrY_V cg09460641	-	-, 15%, **97%**, 5%	-, 55%, **96%**, 13%	-	*Hha*I: non-semen specific, *Gla*I: semen specifc (low signals)
	This study	Non-semen	noSp_Y_ l_A_(B) cg02288797 **noSE-Y-I** or **SE-Y-I **^**#**^	-	-, 87%, 88%, **9%**	-, 90%, 88%, **18%**	-	*Hha*I: non-semen *Gla*I: semen specifc, in both semen assays
	This study	Non-semen	noSp_Y_II_lalu cg04964672 **SE-Y-IV**	-	-, 62%, 84%, **3%**	-, 89%, 92%, **26%**	-	*Hha*I: non-semen specific, *Gla*I: semen specifc, in Y-semen assay
	This study	Semen /non-semen	Sp_Y_III_Gla **noSE-Y-II** or **SE-Y-III**^**#**^/ Sp_Y_III_Sma cg07728631 **noSE-Y-III**	-	-, 90%, 84%, **7%**	-, 86%, 83%, **18%**	-	*Hha*I and *Sma*I: non-semen specific, *Gla*I: semen specifc, in Y-semen assay
	This study	Non-semen	noSp_Y_lV cg23834181	-	-, 68%, 72%, **7%**	-, 59%, 77%, **19%**	-	*Hha*I: non-semen specific
	This study	Saliva	SA_chrY_ll cg25012987	-	-, 86%, 37%, 10%	-, 44%, 47%, 19%	-	*Hha*I: non-semen specific
	This study	Non-saliva	SA(-)-Y-l cg03905640	-	-, 9%, 94%, 94%	-, 81%, 91%, 92%	-	unspecific products
	This study	DC	VK_lalu_I cg15183843	-	-, 96%, 85%, 82%	-, 81%, 80%, 80%	-	*Gla*I: no digest control
	This study	DC	VK_lalu_II cg27254225**	-	-, 58%, 83%, 88%	-, 81%, 89%, 87%	-	PCR failed
	This study	DC	VK_lalu_III cg17913570** **SE-Y-II**	-	-, 65%, 69%, 79%	-, 74%, 70%, 77%	-	*Gla*I: semen specific, in Y-semen assay
	This study	DC	VK_lalu_IV cg05999368**	-	-, 44%, 68%, 68%	-, 71%, 69%, 70%	-	*Gla*I: no digest control
	This study	DC	VK_Gla cg15183843**	-	-, 96%, 85%, 82%	-, 97%, 80%, 80%	-	*Hha*I: digest control, *Gla*I: no digest control

Given beta-values are from the indicated references or own dataset (more information in Online Resource 2). Values in bold indicate concordant beta-values for differentiating one bf between the datasets. Marker names in bold indicate the marker names used in the MSRE/MDRE test-sets. Numbers in bold indicate discriminating beta values. The original names of the markers in this study were chosen based on primer design, the different variants of one locus, the intended tissue specificity, or on the naming from other publications. *GE*, gel electrophoresis; *CE*, capillary electrophoresis; *DC*, digestion control. *CpG site is located on a *Hha*I cutting site; ** CpG sites are 0.5–4.5 kb from the cutting site; # marker is specific for semen or non-semen depending on the assay or digestion enzyme used (*Hha*I or *Gla*I). More information about each CpG site is presented in Online Resources 3 and 4

Besides those reported by Wasserstrom et al. and Lin et al., there are also several identified methylation bf markers based on bisulfite assays [10–12]. However, many of these markers could not be tested because they did not contain any cutting site for *Hha*I, or their beta-values were not appropriate for an MSRE assay. Since bisulfite-based assays can detect both methylated and non-methylated status, the beta-values can be chosen in a different way than for restriction enzyme-based studies. Overall, we tested one additional blood marker, one vaginal secretion marker, and two semen markers from the bisulfite-based studies, and confirmed that all except the blood marker were bf-specific (Table 1).

In addition to markers published in the literature, we also tested newly identified CpG sites with promising beta-values from our database search. We focused on new Y-chromosomal semen markers and markers on autosomal blood- and vaginal-specific sites. Our loci search for further autosomal blood and vaginal secretion markers for the *Hha*I enzyme resulted in two additional promising markers; Bl_chr1_III (cg19637387) for blood and VG chr7_II (cg18502142) for vaginal secretion. However, we did not include Bl_chr1_III in our final multiplex MSRE-PCR, because its PCR product appeared to be 20 bp longer (checked by Sanger sequencing, data not shown) than predicted based on the NCBI reference sequence, leaving us uncertain about its origin. Our pre-testing of the potential vaginal secretion showed that the VG chr7_II marker also appeared for saliva, which can be explained by the moderately increased beta-values (~ 30%) for saliva (for more details see Online Resource 1c).

Examining the beta-values on the Y-chromosomal CpG sites, and searching for semen markers, revealed that none of the Y-chromosomal CpG sites showed semen-specific beta-values for *Hha*I digestion. Rather, we only found the opposite: low beta-values for semen and high values for the other bfs. Therefore, we tried to design markers that used a methylation-dependent restriction enzyme, like *Gla*I. This method could enable attainment of a semen-specific signal after *Gla*I digestion, and a non-semen-specific signal with *Hha*I digestion. To combine a methylation-sensitive and methylation-dependent enzyme in one approach, we had to replace *Hha*I with *Sma*I, because *Hha*I and *Gla*I have the same cutting motif. We tested a total of 16 Y-chromosomal markers, five intended to be specific for blood, two for saliva, four for semen, and five as digestion controls for the *Gla*I enzyme. Surprisingly, the tests showed that 10 of the tested markers had a semen or non-semen specificity (depending on the used enzyme). Hereby the markers intended for blood or saliva showed also a non-semen specificity (after *Hha*I) and even one intended digestion control, cg17913570, appeared to be semen-specific after *Gla*I treatment.

Overall, we tested 33 autosomal markers (16 from literature and 17 from own database search) and 16 new Y-chromosomal markers, of which 14 autosomal loci and 10 Y-chromosomal loci were determined to be bf-specific (complete summary Online Resource 1c).

### Design of body fluid MSRE/MDRE multiplex-PCR

**Fig. 1:**
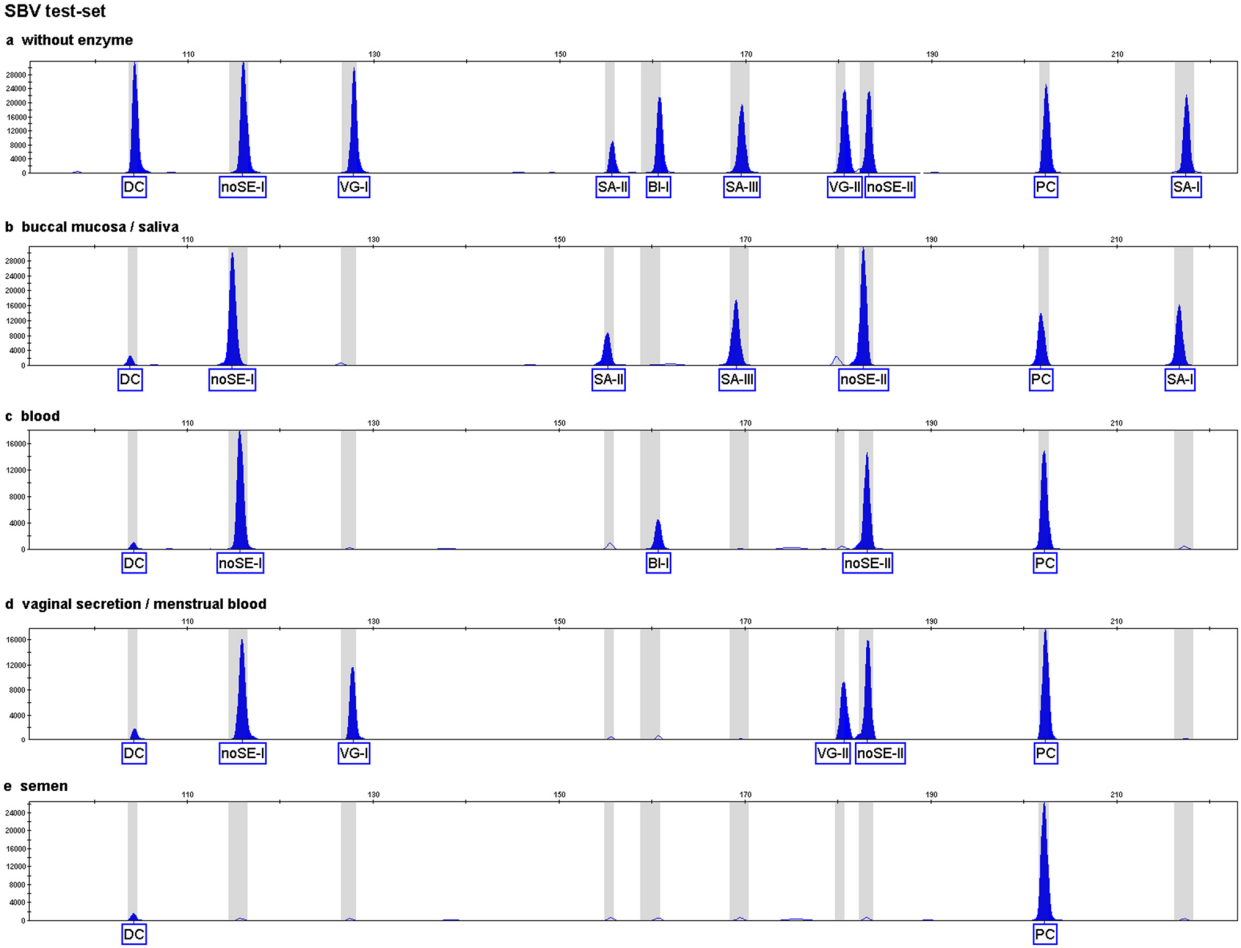
Different tissue-specific methylation patterns of the saliva-blood-vaginal secretion (SBV) test-set with one ng of DNA samples after capillary electrophoresis (Y-axis in RFU, X-axis in bp)

The 17 loci identified from our search were used to develop three different test-sets. First, we developed a saliva-blood-vaginal (SBV) test-set that includes a blood marker (BL-I), three saliva markers (SA-I, SA-II, and SA-III), two vaginal secretion markers (VG-I and VG-II), and two non-semen markers (noSE-I and noSE-II). When the SBV test-set was performed using DNA from blood, buccal mucosa, saliva, vaginal secretion, or semen, we obtained four different tissue-specific methylation patterns (TSMPs) for blood, saliva/buccal mucosa, vaginal secretion, or semen (Fig. 1b–e). However, because our SBV test-set does not contain any semen-specific markers, semen can only be detected indirectly because of the missing non-semen markers (Fig. 1e). We observed the same TSMP for both menstrual blood and vaginal secretion, because the BL-I marker was not detected in menstrual blood.

**Fig. 2:**
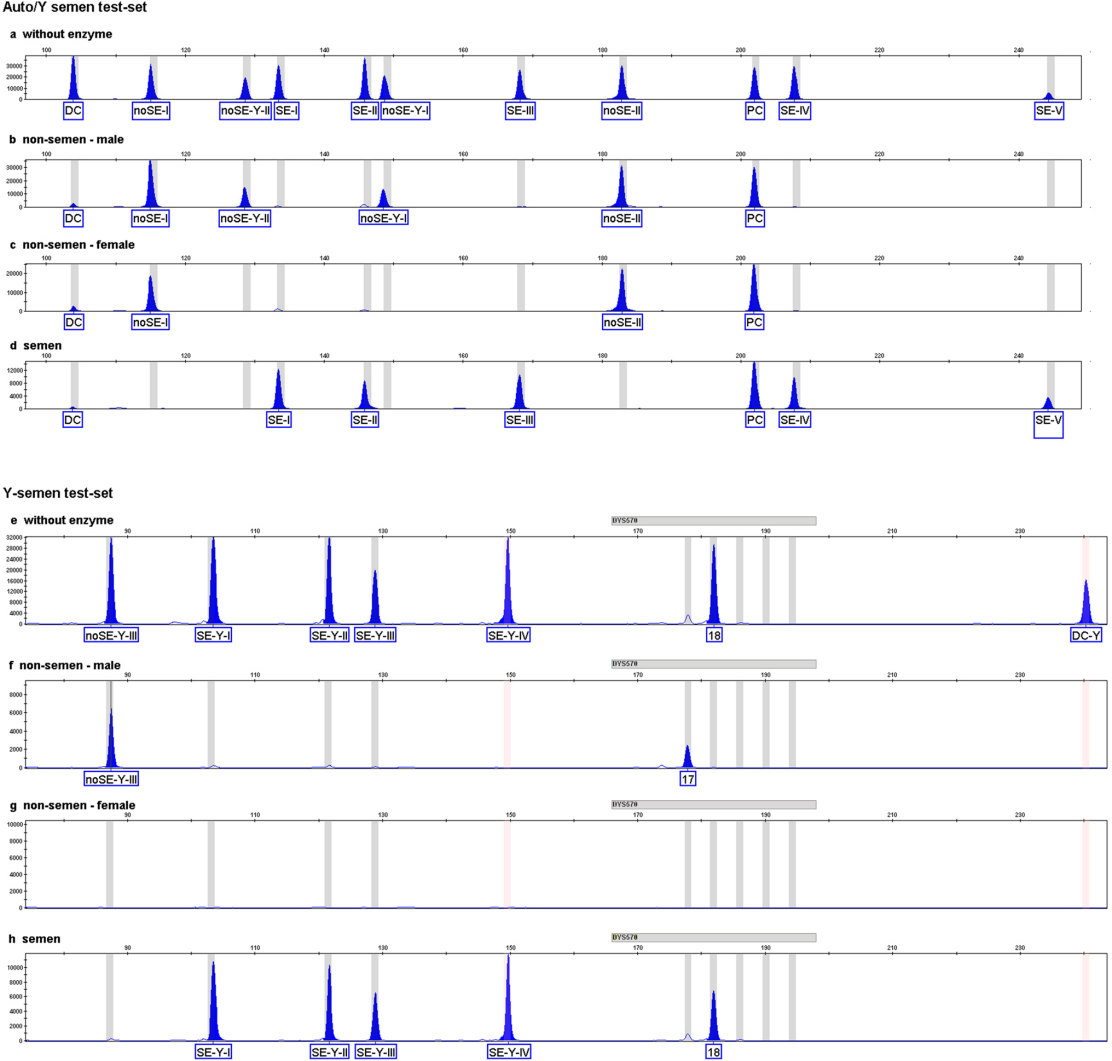
Different tissue-specific methylation patterns of the auto/Y- and Y-semen test-sets with one ng of DNA samples after capillary electrophoresis (Y-axis in RFU, X-axis in bp). **a**–**d** Tests of different bf with the auto/Y-semen test-set, **e**–**h** tests of different bf with the Y-semen test-set. Non-semen male methylation pattern (**b** and **f**) is obtained from the tested bf from male blood, buccal mucosa, and saliva samples. Non-semen female methylation pattern (**c** and **g**) is obtained from the tested bf from female blood, buccal mucosa, saliva, vaginal secretion, and menstrual blood samples

Because semen is the bf of greatest interest in sexual assaults, we also designed two additional semen test-sets: one using only Y-chromosomal markers, termed the “Y-semen test-set,” and one including Y-chromosomal and autosomal markers, termed the “auto/Y-semen test-set.” The auto/Y-semen test-set includes five autosomal semen-specific markers (SE-I, SE-II, SE-III, SE-IV, and SE-V), as well as two autosomal non-semen markers from the SBV test-set (noSE-I and noSE-II) and two additional Y-chromosomal non-semen markers (noSE-Y-I and noSE-Y-II). Testing the auto/Y-semen test-set with DNA from blood, menstrual blood, buccal mucosa, saliva, vaginal secretion, or semen resulted in three different TSMPs: one TSMP for non-semen DNA from male persons, including the two Y-chromosomal non-semen markers (noSE-Y-I and noSE-Y-II), and another TSMP for female persons, which lacks the non-semen Y-chromosomal markers (Fig. 2b, c). The third TSMP for the auto/Y-semen test-set is specific for semen (Fig. 2d). Both the auto/Y-semen and the SBV multiplexes utilize the same digestion control (DC) and the same PCR control (PC) [6, 7].

The second semen test-set contains only markers on the Y-chromosome (noSE-Y-III, SE-Y-I, SE-Y-II, SE-Y-III, and SE-Y-IV). Two of these markers are the same loci as from the auto/Y-semen multiplexes, noSE-Y-I and noSE-Y-II. However, in the Y-chromosomal semen test-set, these markers change their tissues specificity from non-semen to semen, because here they are digested using the methylation-dependent enzyme *Gla*I. For example, for the locus cg02288797, we designed two primer pairs: one that is used for *Hha*I digestion (noSE-Y-I) and is thus non-semen-specific and one that is the target for *Gla*I digestion (SE-Y-I) and is thus semen-specific. The locus cg07728631 is even more complex because it covers three markers. Here, we also used two primer pairs: one that contains a *Sma*I cutting site (noSE-Y-III) and one that contains both a *Gla*I (SE-Y-III) and a *Hha*I (noSE-Y-II in auto/Y-semen test-set) cutting site (Table1, Online Resource 2 and 4). Furthermore, an additional PCR product is generated from the forward primer for marker noSE-Y-III and the reverse primer for the marker SE-Y-III, since these two markers are located near each other (Online Resource 4 page 9). We used that additional PCR product as a digestion control (DC-Y). No signal of DC-Y indicates a positive digestion by *Gla*I or/and *Sma*I. Unfortunately, we did not find digestion controls, which would contain a cutting site only for either the enzyme *Gla*I or *Sma*I. The Y-semen test-set will be negative for all female samples and, therefore, negative for vaginal secretion and menstrual blood. With the Y-semen test-set, we obtained only two different TSMPs: one for non-semen from male persons and one for semen (Fig. 2f, h). Additionally, we combined the Y-semen test-set with one Y-STR, DYS570, which serves as a positive PCR control [14].

### Specificity tests

**Fig. 3:**
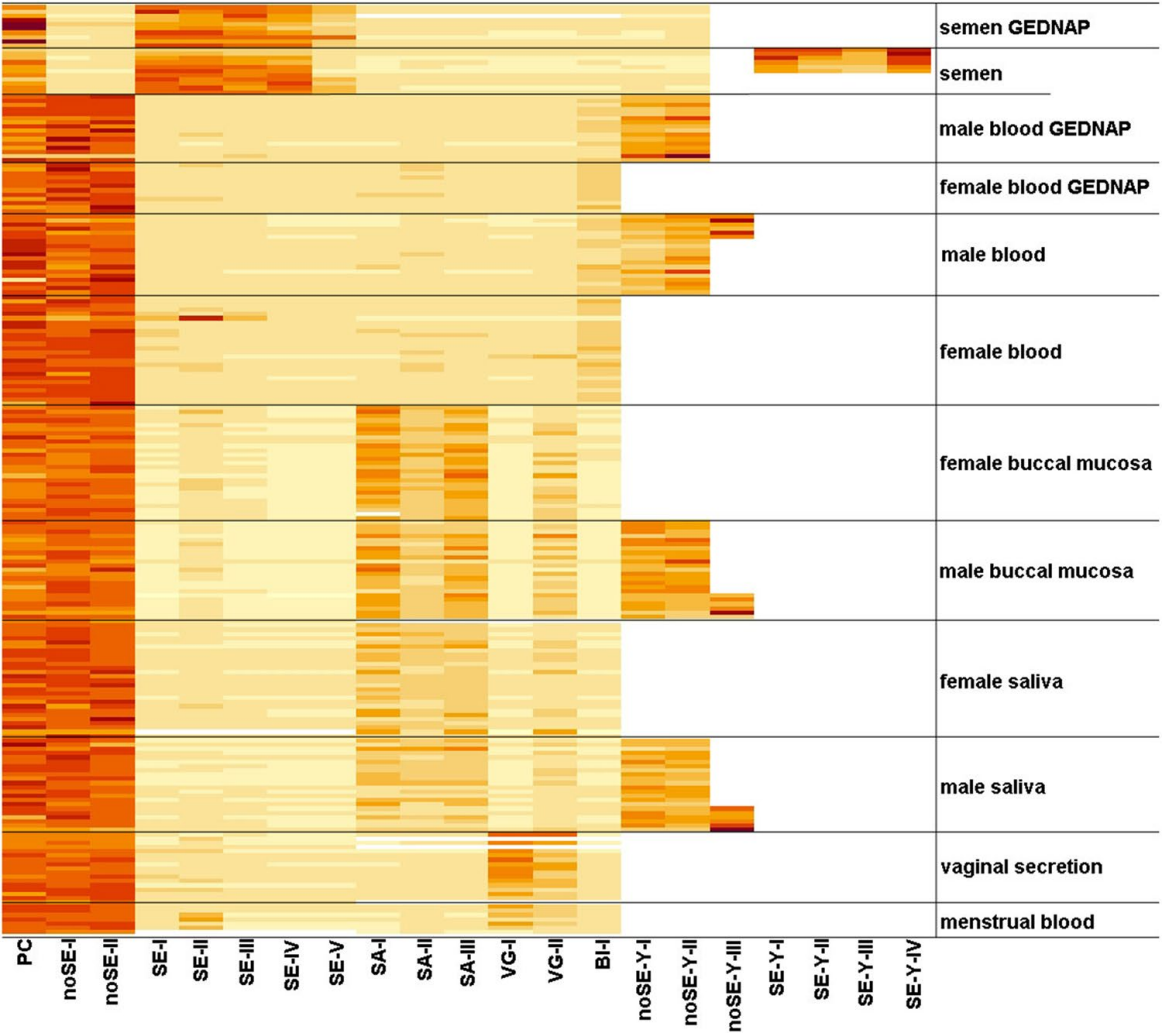
Heatmap of the saliva-blood-vaginal and semen test-sets from tested samples. Yellow to red color scale presents high to low digestion score values. Number of samples: 10 semen, 49 buccal mucosa, 49 saliva, 46 blood, 7 menstrual blood, and 17 vaginal secretion samples from own sample collection, as well as 38 blood and 10 semen GEDNAP samples. White gaps represent no data, because no Y-chromosomal marker can be detected for female samples, and because the Y-semen test-set was applied to a smaller assortment of 6 samples from our own collection for each body fluid (bf)

To validate our three bf approaches, we tested them using different bfs from our own sample collection, and available interlaboratory GEDNAP samples from the last 8 years. Our tests showed no significant differences between fresh collected DNA samples and the stored GEDNAP samples (Fig. 3, Online Resource 1d). With all approaches, the best results for specificity and PCR performance were obtained from the semen and non-semen markers (Fig. 3, Table 2). The only exceptions were small false-positive rates for SE-I, SE-II, and SE-III in blood and saliva (Table 2). However, these false-positives showed a peak ratio of only 2 compared to an average ratio of 20 for semen, such that correct and false-positive reactions could be clearly distinguished. Additionally, for the marker SE-II, we obtained a significant rate of false-positives for several bfs (Table 2). Although the signal intensities of these false-positives were not the same as for the intended bf semen (Fig. 3), the marker SE-II is not truly semen-specific and it should be interpreted with caution. The semen marker SE-V showed no false-positive results, but showed false-negative results in almost 30% of the tested samples. We think that this was not due to low specificity of the marker, but rather because of reduced PCR amplification efficiency. Also reactions without enzyme often showed a reduced signal intensity for SE-V (Fig. 2a). The three saliva markers SA-I, SA-II, and SA-III showed a false-negative rate of about 20% for the bf saliva, but not for buccal mucosa. The signal intensities, and thus a clear TSMP, for saliva were moderate-to-good depending on the DNA source. We predominantly obtained better signals for buccal mucosa than for saliva. Similar to the saliva markers, for the vaginal secretion markers VG-I and VG-II, we obtained good signal intensities and thus clearly interpretable TSMPs. However, for VG-II, we observed a very high false-positive rate for both saliva and buccal mucosa. The most difficult marker was the BL-I marker, which showed only very weak PCR efficiency after digestion, possibly caused by the relatively low methylation levels for blood (Table 1). The BL-I marker often showed a ratio of only 2, which commonly gave rise to no clear TSMP and a very high false-negative rate of 50%. On the other hand, the BL-I marker showed no false-positive results. Nevertheless, false-negative results were always identified as non-semen body fluids, by the non-semen markers.

**Table 2:** Summary of false-negatives and false-positives

	Indented marker specificity	Methylation marker	Semen	Buccal mucosa	Saliva	Blood	Menstrual blood	Vaginal secretion
			Error rate	Error rate	Error rate	Error rate	Error rate	Error rate
Autosomal	Control	PC	No	No	No	No	No	No
	Non-semen	noSE-I	No	No	No	No	No	No
		noSE-II	No	No	No	No	No	No
	Semen	SE-I	No	no	no	4% fp*	No	No
		SE-II	No	**41% fp**	**12% fp**	7% fp*	**67% fp**	No
		SE-III	No	2% fp*	no	4% fp*	No	No
		SE-IV	No	No	No	No	No	No
		SE-V	**29% fn**	No	No	No	No	No
	Saliva	SA-I	No	2% fn	**16% fn**	1% fp	No	No
		SA-II	No	2% fn	**18% fn**	4% fp	No	No
		SA-III	No	4% fn	**24% fn**	No	No	No
	Vaginal secretion	VG-I	No	No	No	2% fp	-	7% fn
		VG-II	No	**73% fp**	**41% fp**	2% fp	-	No
	Blood	BL-I	No	No	No	**50% fn**	No	No
Y-chromosomal	Non-semen	noSE-Y-I	No	No	No	No	No	No
		noSE-Y-II	No	No	No	3% fn	No	No
		noSE-Y-III	No	No	No	No	No	No
	Semen	SE-Y-I	No	No	No	No	No	No
		SE-Y-II	No	No	No	No	No	No
		SE-Y-III	No	No	No	No	No	No
		SE-Y-IV	No	No	No	No	No	No

Number of samples: 10 semen, 49 buccal mucosa, 49 saliva, 46 blood, 7 menstrual blood, and 17 vaginal secretion samples from own sample collection, as well as 38 blood and 10 semen GEDNAP samples. The Y-semen test-set was applied to a smaller assortment of 6 samples from own collection for each body fluid (bf). *fp*, false-positive; *fn*, false-negative; *no*, no fp or fn were observed. Error rate cannot be calculated, because the proportion of vaginal fluid in the menstrual blood sample was not determined, * markers showed only small ratios of 2 compared to the average ratio of 20 in semen

### Test of sensitivity and the presence of excessive amounts of female DNA in mixtures

**Fig. 4:**
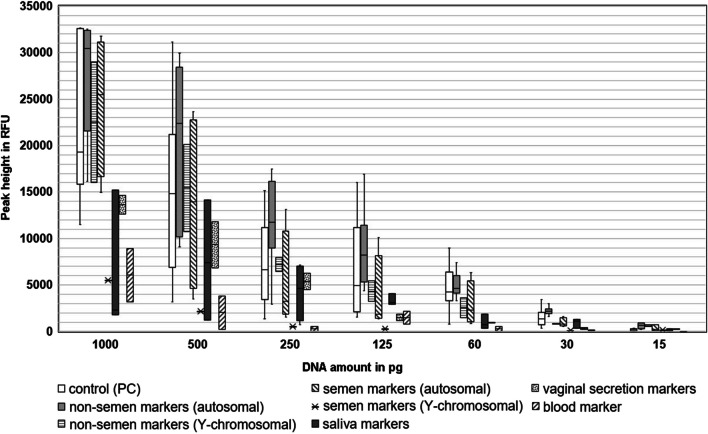
Boxplot diagram of tested dilution series with the saliva-blood-vaginal secretion (SBV) and semen test-sets. In total, we tested 12 dilution series (two from blood, saliva, buccal mucosa, vaginal secretions and four from semen) for the SBV and the auto/Y-semen test-sets, and two dilution series (one male buccal mucosa, and one semen) for the Y-semen test-set

With the SBV and auto/Y-semen approaches, we tested twelve dilution series of different bfs, from 1 ng down to 15 pg DNA (Fig. 4). We observed complete and meaningful results with up to 125 pg DNA input. The only exception was in a blood dilution series, where the BL-I marker dropped out and resulted in a non-semen TSMP (Fig. 1F). When both MSRE-multiplexes were conducted with about 60 pg of input DNA, we observed only 50% valid TSMPs, which dropped down to almost zero when the SBV test-set was conducted with 30 and 15 pg of DNA input. However, the auto/Y-semen test-sets with low DNA input yielded about 50% interpretable TSMPs. The increased sensitivity of the auto/Y-semen test-set is not surprising, since the semen and non-semen markers showed a higher methylation level and correspondingly better PCR amplification efficiency.

We also tested the Y-semen test-set with two dilution series (Fig. 4). We observed that the peak heights of the Y-semen marker were generally lower than the markers in the autosomal test-set, or the non-semen marker noSE-Y-III in the Y-semen test-set. This cannot be due to the markers themselves because, for example, the marker SE-Y-III in the Y-semen test-set is exactly the same marker (same primers) as the marker noSE-Y-II in the auto/Y-semen test-sets. The only difference is that the Y-semen test-set involves use of the enzyme *Gla*I instead of *Hha*I (auto/Y assay). Both enzymes cut the motif GCGC, but the *Gla*I enzyme also cuts further sites because its recognition site is RCGY. Therefore, the noSE-Y-II marker has two *Hha*I cutting sites, while the SE-Y-III marker has three *Gla*I cutting sites (Online Resource 2 and 4 page 8). It is unclear whether this is the only explanation for the lower signals after digestion with the *Gla*I enzyme. However, similar to the auto/Y-semen MSRE multiplexes, the Y-semen test-set also showed a clear TSMP with lower input DNA.

**Fig. 5:**
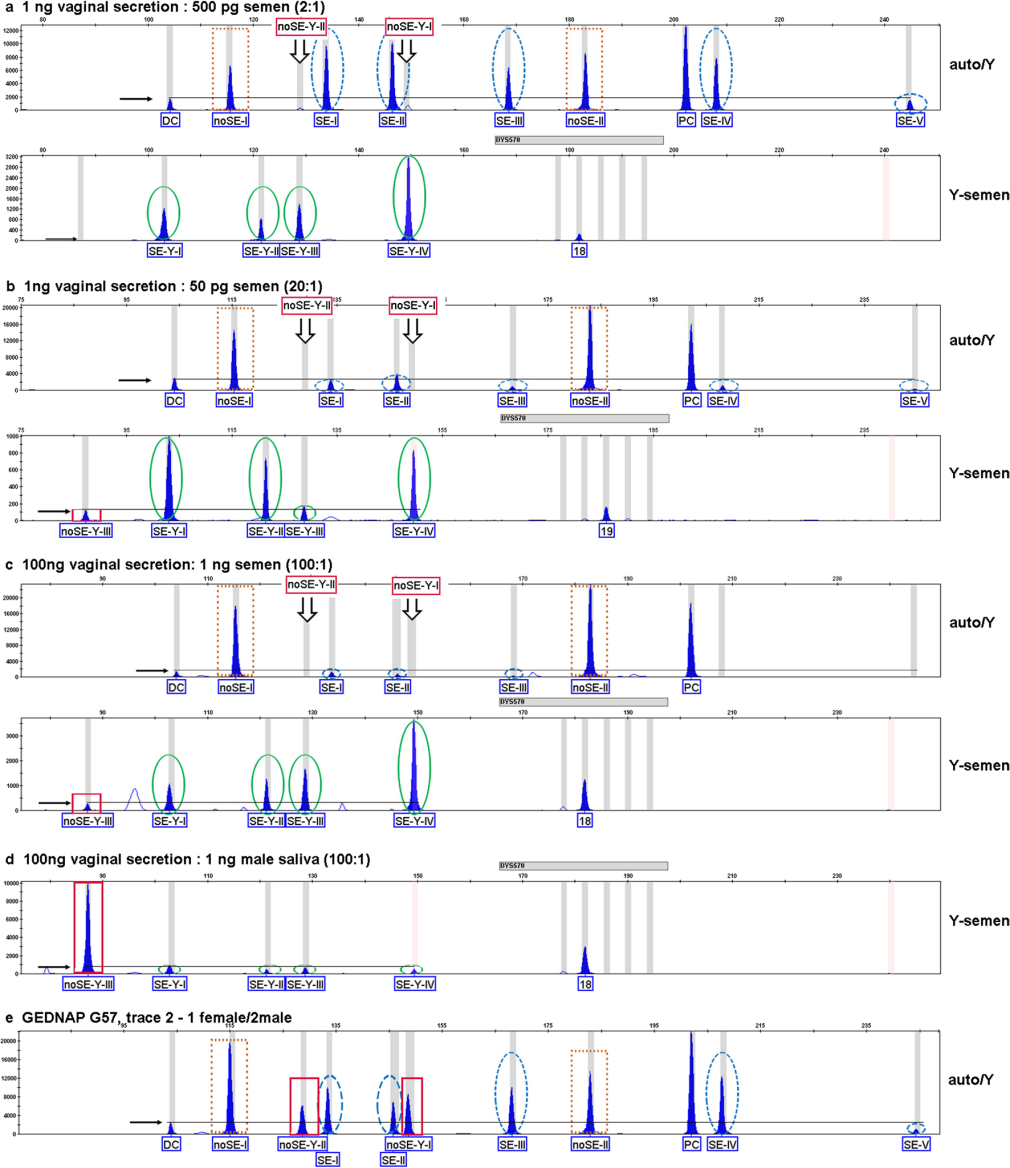
Different tissue-specific methylation patterns of the auto/Y-semen test-set (containing two Y-chromosomal markers) and Y-semen test-sets from mixtures after capillary electrophoresis (Y-axis in RFU, X-axis in bp). Horizontal arrows indicate the peak height of the digestion controls. Only peaks that are higher than the digestion control can be interpreted as positive. Vertical arrows indicate positions of Y-chromosomal markers noSE-Y-I and noSE-Y-II in the auto/Y-semen test-set. In mixtures with female DNA and semen DNA, these markers do not appear and, therefore, indicate the presence of semen. Rectangle = autosomal non-semen markers, dotted rectangle = non-semen Y-chromosomal markers, oval cycles = Y-chromosomal semen markers, dotted oval cycles = autosomal semen markers

Furthermore, we also tested different mixtures for the semen test-sets, with a minor male component (semen) and excessive amounts of female DNA (vaginal secretion), as it is often seen in sexual offenses. The test of a semen/vaginal secretion mixture with a 1:2 ratio showed a mixed TSMP of autosomal non-semen and autosomal semen markers in the auto/Y-semen test-sets (Fig. 5a). The absence of the non-Y-chromosomal semen markers indicates that the non-semen part originated from the female individual. This result was confirmed by the Y-semen test-set, which also showed clear Y-chromosomal semen markers, and a proportional response of the digested Y-chromosomal non-semen marker. When examining male DNA mixed with a greater proportion of female DNA (1:5, 1:20 and 1:50 ratio), the autosomal semen marker could be only detected in 50% of the tested mixed samples, but interpretation in these cases was more difficult because semen markers were often close to the digestion threshold (Fig. 5b). No autosomal semen marker could be detected for semen/vaginal secretion mixture with a ratio of 1:100 (Fig. 5c). Testing the mixtures with the Y-semen test-set yielded improved resolution. Here, we could detect the Y-chromosomal semen markers in all analyzed mixtures, as long as the male component did not fall below 200 pg. For lower DNA amount of semen DNA of 100 pg, Y-semen markers could be detected in 90% and for 50 pg semen DNA input only in 60% of the tested mixed samples (Fig. 5b). However when DNA input was sufficient, the male component could be clearly detected by Y-chromosomal semen and non-semen markers, also for 1:100 male/female mixtures (Fig. 5d).

Among the samples from our study, we tested so far only semen/vaginal fluid mixtures, but also one GEDNAP sample included a mixture of blood and semen from one female and two males (Gednap 57, trace 2, 2018). Hereby, the RSID results can not reveal whether the blood component was only from the female person, or was from the female and one of the male persons. However, the autosomal semen test-set showed the presence of all markers, along with the Y-chromosomal non-semen markers. This indicates the presence of a semen and another male non-semen component. This strongly indicates that the second male sample is a blood sample.

## Discussion

### Loci search

Our loci search was conducted by testing previously published tissue-specific methylation markers, as well as by filtering methylation datasets from the Infinium HumanMethylation450 Beadarray for new markers (all literature in Online Resource 1b and Online Resource 2). Our first intention to validate the two published MSRE assays from Wasserstrom et al. [6] and Lin et al. [7] was only partially successful. In particular, the establishment of markers specific for semen or non-semen was successful, because semen exhibited many CpG sites with high methylation differences from other bfs. Moreover, these discriminating CpG sites showed high methylation beta-values of over 90%, which enabled the design of a marker with several cutting sites, thereby increasing specificity. After *Hha*I digestion, PCR amplification is more efficient when 90% of the input DNA is not cut, which results in clear TSMPs. On the other hand, if markers have a low methylation value, it can reduce the success of PCR after digestion. We think that this explains why we could not detect the markers SA-II, BL-I, and BL-II after digestion with *Hha*I. The beta-values of these markers are reported to be between 16–34%. Therefore, even for the specific bf, the major part of the input DNA is digested after *Hha*I digestion. This was confirmed for the markers BL-I and SA-II, because new variants with fewer cutting sites resulted in successful PCR amplification for the intended bfs after *Hha*I digestion.

It remains unclear why these markers were successful when tested by Lin et al. [7]. A likely explanation is that PCR efficiency strongly influences markers with low beta-values, as well as markers with small methylation differences between the different bfs. Small differences in PCR conditions such as single components, different PCR cyclers can strongly affect the results with such markers. Especially for sequences with high GC content, which are known to be more complicated to amplify, small differences in PCR conditions can result in a different outcome. Notably, in our study, we had to use GC-enhancer in our PCR reaction to obtain proper amplification, which highlights that GC markers are more challenging targets for PCR. We think this may also explain why we observed some inconsistencies between the results of gel electrophoresis (GE) versus capillary electrophoresis (CE). In our loci search, for economic reasons, we often tested new markers first by GE, for which we used same cycling conditions but with four additional cycles because GE is less sensitive than CE. From GE tests, we identified 10 loci with a possible specificity for blood or vaginal secretion. However, these observed results from GE were only confirmed by CE for two markers: VG-7-II and Bl_chr1_III (Table 1). In contrast, for semen-specific markers, we did not observe significant differences between GE and CE; however, markers with smaller methylation differences showed differences between the two methods, as for blood and vaginal secretions. This further confirms that PCR efficiency plays a critical role in MSRE/MDRE assays, and that kits should be designed very carefully and with equally selected PCR components. It also leaves the possibility that markers found not to be bf-specific by GE might be positive in CE, although the stability of these markers could be questioned. Therefore, in Table 1, we indicated markers that showed good beta-values but negative results after GE, and which thus might warrant further testing by CE.

In summary, we tested 33 autosomal loci with additional 34 variants with different number of cutting sites, from which we identified 14 loci as body fluid-specific. Only two of the autosomal specific markers were derived from our database search, in which we focused on blood and vaginal secretion (Table 1). Therefore, it seems that the 450K array has been well-examined for new markers for MSRE using *Hha*I, at least for the body fluids blood and vaginal secretion. We found that it was especially difficult to find specific blood markers, because hypermethylated CpG sites in blood always corresponded with hypermethylated CpG sites in saliva, which may be related to the presence of blood leukocytes in saliva [15]. However, a few markers showed promising beta-values from the 450K array dataset but had no *Hha*I cutting site (Online Resource 2). A total of 17 autosomal markers from our loci search did not exhibit a *Hha*I cutting site and could thus not be used for our MSRE assays. For these markers, it would be interesting to try other methylation-sensitive restriction enzymes, such as *Hpa*II, which has been already successfully tested in an MSRE assay by Senst et al. [16]. Furthermore, our loci search revealed that the CpG sites from the 450K array only cover a small fraction of one CGI and that the beta-values of different CpG sites from the same island can substantially change in MSRE-PCR. Therefore, our loci search may have excluded CGI with candidate CpG sites because of the exclusion of the exemplary chosen CpGs of the 450K array.

To differentiate male/female mixtures containing different bfs, we also searched for Y-chromosomal bf markers. Since this is a new approach and there are no available data in the literature, we could only use the data from the 450K array, which unfortunately only covers 416 Y-chromosomal CpG sites. In contrast to the autosomal markers, we found only Y-chromosomal markers that were hypomethylated in semen and hypermethylated in the other bfs. Therefore, we used the methylation-dependent restriction enzyme *Gla*I for MDRE-PCR, in order to obtain semen-specific markers rather than non-semen-specific markers, as we would obtain using a methylation-sensitive restriction enzyme. Our test confirmed a general hypomethylated Y-chromosome in semen. We looked up 44 Y-chromosomal CpG sites, of which we tested 16 loci with 18 additional variants (Online Resource 2) for different intended bf specificities (e.g., blood, saliva, and semen) or as a Y-chromosomal digestion control (Table 1). However, independent of whether the marker was expected to be specific to blood, saliva, or another bf based on its beta-values, we found only non-semen-specific markers after *Hha*I digestion. On the other hand, these markers were expected to be semen-specific after *Gla*I digestion, and this was confirmed for all markers tested with *Gla*I (Table 2, Online Resource 2). From these initial tests of Y-chromosomal markers, it seems that the data from the 450K array are highly erroneous regarding Y-linked data, since we could confirm almost none of the bf specificities predicted by beta-values. Even a marker that should have been hypermethylated in all bfs was always digested after MSRE with *Hha*I, supporting that CpG sites on the Y-chromosome in semen are generally hypomethylated. Accordingly, previous studies have reported high percentages of hypomethylated CpGs in the sex chromosomes of the sperm methylome, supporting their essential role in spermatogenesis [17]. Our presently observed high error rate for Y-chromosomal CpG sites on the 450K array might be due to cross-reactive probes. Chen et al. reported enrichment of cross-reactive probes co-hybridizing to the sex chromosomes, and resulting in false-positive autosomal sex-associated methylations [18, 19]. It seems likely that Y-linked probes may also show cross-reactivity to other genomic region, e.g., on the highly homologous X-chromosome, leading to false data for Y-chromosomal CpG sites [20]. Nevertheless, it was easy to find Y-chromosomal markers that show different TSMPs between semen and other bfs, as already observed for the autosomal markers. No markers could be obtained for other bfs, or even for a *Gla*I digestion control, which would require a hypermethylation in all bfs, as in semen. In summary, the 450K array chip from Illumina seems to produce only limited data for Y-chromosomal CpG sites, due to the erroneous observed data, but also because the Y-linked CpGs are highly underrepresented with only 416 markers [21]. Although the 450K array provides much better coverage for autosomal CpGs than for Y-chromosomal CpGs, it could be promising to expand the loci search to other databases, for example, from Epic Beads Chip or whole-genome methylation sequencing studies.

### Specificity tests

We tested the markers in our MSRE assays for specificity among 182 bf samples from own collection. Furthermore, we also used 49 GEDNAP samples collected over the last 8 years. We found no significant differences in results from the old GEDNAP samples versus our own sample collection; therefore, we pooled these data together for plotting and evaluation of false-negative and -positive ratios. The specificity of each marker was determined after a digestion score.

The specificity tests revealed that semen was the bf that could be best determined, which is in concordance with all previously published literature, since these markers show the highest differences in methylation level between bfs. Furthermore, the high difference in methylation level between semen and non-semen allows the design of a marker with several cutting sites and, therefore, increased specificity between the different bfs. We tested five autosomal semen markers, of which three performed with high specificity and led to clear TSMPs. Two semen markers in the auto/Y-semen approaches did not perform correctly. First, the marker SE-V exhibited a high false-negative rate, likely due to problems with PCR amplification. Tests with different primer variants for SE-V even yielded non-specific products (Online Resource 2). For the marker SE-II, we observed a high rate of false-positives, especially for buccal swabs. The marker SE-II was first discovered by Wasserstrom et al. (L7), but was reported to be only semen-specific [6]. Here, we used same primer sequences as Wasserstrom et al., but different PCR conditions, which might explain the differences in the obtained results. However, SE-II is designed with only one cutting site, which is unfortunate because this locus showed high beta-values. Therefore, it may be promising to test new variants with more cutting sites.

The saliva markers SA-I, SA-II, and SA-III yielded clear TSMPs for buccal mucosa, with only a low number of false-negatives for SA-I and SA-III. Notably, in the composed interpretation of all three markers, samples from buccal mucosa could be clearly identified. For saliva samples, the specificity of these three SA markers decreased by about 20%. The DNA content of buccal swabs versus saliva are derived in different proportions from buccal cells (epithelial cells), blood leukocytes, and cell-free DNA with a clearly higher proportion of epithelial cells in buccal swabs than in saliva [15, 22]. It is likely that the observed low specificity of the saliva markers is due to the decreased epithelial cell content in saliva and that the SA markers are specific to epithelial cells from buccal mucosa. Our database search results did not enable differentiation between saliva and buccal mucosa, because this detailed information was often unavailable. An exception was the study of Lee and Park et al., which contributed data for 8 vaginal fluid, 9 menstrual blood, 6 blood, and 4 saliva samples to the database. The NCBI database harbors mainly medical studies of human tissues, which makes it more difficult to extract data for forensically relevant bfs. The increasing importance of epigenetic markers supports the necessity of developing a specific forensic methylation database, which could also drive a common nomenclature for the markers [3, 23]. Except for the numbering of the Illumina Chips, there is presently no distinct naming of CpG sites [24].

Vaginal secretion could not be determined in all samples, because VG-I exhibited a false-negative rate of 7%. On the other hand, the newly tested marker VG-II showed no false-negatives, but appeared in 70% of buccal mucosa and 40% of saliva, which is in concordance with our pre-test of VG-II. The increased value for buccal mucosa could again indicate specificity for buccal mucosa cells. Overall, the VG-II marker can roughly be defined as a non-semen, non-blood marker. However, the 30% false-negative reactions in buccal mucosa also lead to uncertain interpretations. Therefore, vaginal secretion identification is insufficient with the current SBV assay and must be improved with further markers for vaginal secretion.

For blood, we tested only one marker, BL-I, which showed low PCR amplification. Therefore, we could identify only 50% of the tested samples as blood. However, we did not observe any false-negative results. In contrast to the findings of Lin et al., we did not detect the BL-I marker in menstrual blood [7]. Therefore, testing menstrual blood samples yielded the same TSMP as for vaginal secretion. The SBV MSRE multiplex must be improved with additional blood markers for more reliable interpretations. Notably, the presently obtained results for the bfs vaginal secretion and menstrual blood are insufficient due to the small sample sizes.

### Challenges of the method

The aim of using MSRE/MDRE-PCR for bf detection is to produce clear TSMPs, which depends on several conditions. The first condition is significant differences between tissues in the methylation states of the CpG sites. The second condition is complete digestion of the input DNA or the target sites. This second condition is highly influenced by the methylation level of the markers. No CpG site is 100% methylated or non-methylated; after digestion, there will always be remaining uncut DNA. With good markers, the amount of leftover uncut DNA will be too small for PCR amplification, such that there is no signal. Therefore, the PCR efficiency of the single markers also plays an important role in MSRE/MDRE-PCR, which means that standardization is required for interlaboratory usage of common assays. However, complete digestion also depends on the input amount of DNA. Therefore, it is important to include digestion controls in the assays, which can be used to judge successful digestions, and to determine a threshold for uncut DNA. In our assay, we used the DC from Wasserstrom et al. [6]. We could not use the published DC from Lin et al., because it hampered the amplification of SA-III in the multiplex PCR (data not shown). Nevertheless, we recommend the use of several DC controls for better estimation of complete digestion. In particular, when PCR design makes it necessary to work with different fluorescent dyes in CE, we recommend a DC for each color. Additionally, the selected restriction enzyme should be able to perform in PCR buffers. Levenson and Melnikow have suggested that a type IIp enzyme should be selected and that type IIe enzymes should be avoided because they need two recognition sites for cutting [25].

The biggest challenge of MSRE/MDRE-PCR are complex bfs that comprise several cell types, such as blood or saliva. Due to the nature of this method, all DNA from different cell types is analyzed for the intended target sequence. Consequently, MSRE/MDRE-PCR reveals the sum of the methylation status of the target loci from all cell types in one bf. This also means that a search for markers for MSRE/MDRE PCR should be conducted from methylation data from precise bfs and not based on known specific gene expressions. The occurrence of a bf-specific mRNA or specific protein does not mean that the expected hypomethylation of this gene can be observed for all of the cell types constituting this bf. Notably, mRNA-based bf detection methods are reportedly difficult for complex bf, especially for the separation of saliva and vaginal secretion [26]. Particularly for MSRE/MDRE-PCR, increasing complexity of the bf composition will mean that it is more complicated to identify highly specific markers as we observed, for example, in the search for blood markers. Here, it was difficult to find CpG sites that showed good differentiation between saliva and blood. The use of a restriction enzyme in MSRE/MDRE-PCR also limits the design of markers. However, in this study, we demonstrate that it is possible to use different restriction enzymes, and even to combine methylation-sensitive and -dependent enzymes in one assay. Importantly, the detection of a specific bf does not depend on the specificity of a single marker, but is rather the result of the combination of different markers in a TSMP. This allows some non-specificity for the single markers, as well as opens the possibility of using markers with different information loads. For example, in this study, we tested autosomal and Y-chromosomal non-semen marker, which do not identify a specific bf but, in combination with other markers, they can increase the information that can be deduced from the TSMP. Therefore, it would be useful to design new markers, such as non-vaginal markers or non-vaginal/non-semen markers. The use of markers with different information loads can increase the specificity, as well as the numbers of possible assay designs.

### Applicability in forensic case work and further perspectives

In forensic case work, DNA analyses are often challenging due to poor quality samples with low DNA concentration and degraded DNA. Previous studies have shown MSRE assays have sensitivity almost equal to common STR analysis. Lin et al. reported robust amplification of their 10 plex MSRE-PCR in combination with STR multiplex PCR Minifiler with DNA input of 250 pg [7], and Wasserstrom et al. reported successful bf detection with their semen markers and DNA input of 148 pg [6]. In our study, we also tested different amounts of input DNA, and found similar results, with clear TSMPs when using as little as 125 pg DNA input. Only with the marker BL-I, we observed also a drop-out at 500 and 250 pg in one dilution series. On the other hand, several markers, especially the semen and non-semen markers, also performed well with lower amounts of DNA than 125 pg.

In addition to issues regarding DNA quality, forensic samples can also appear as complex DNA mixtures of different bfs from one or more persons. The most common scenario involving bf mixtures and their importance on court are mixtures of vaginal secretion/semen traces taken from victims after sexual assaults. These samples are characterized by an extensive amount of female DNA relative to a minor male component. In such cases, the STR profile of the male person is often not detectable or difficult to interpret. In this situation, the male component can be proved by Y-chromosomal STR analyses [27]. Similar to Y-STR typing in male/female mixtures, we designed a novel Y-chromosomal semen assay to avoid competition between PCR components. We tested our semen markers with female/male mixtures composed of an excessive amount of vaginal secretion plus a minor semen component. Our results showed that the autosomal semen markers could be clearly detected up to a mixed ratio of 1:2 (semen/vaginal). For increased ratios up to 1:50 (semen/vaginal) the autosomal semen markers were missing or more difficult to interpret. On the contrary with the Y-chromosomal semen markers from the Y-semen test-set, we could detect semen even for ratios of 1:100 (semen/vaginal). Only when the male component was lower than 100 pg, also the Y-chromosomal markers started to drop out (Fig. 5b SE-Y-III). A resolution of even higher ratios than 1:100 should be possible. In general, the Y-semen markers strengthen the proof of semen even when autosomal markers provide no clear results. Lin et al. reported detection of their autosomal semen marker in the 3-plex assay with ratios of 1:50 for blood and even 1:800 for vaginal secretion [8]. Interestingly, with the 10-plex assay containing two autosomal semen markers they could detect semen with ratios only up to 1:10 [7]. The higher resolution of the semen component in male/female mixtures reported by Lin et al. could be due to a different setting of thresholds. They validated the marker specificity based on their ratio to PC control, and not according to DC as in our study. The way Lin et al. analyze their reaction postulate a complete digest and also that the portion of uncut semen markers will not be amplified. They reported their DC always as complete digested; however, this does not necessarily be the same case for the bf markers. When we tested the DC form Lin et al., we also obtained always a complete digest for this DC, which might be an overestimated digest indicator for the other markers. As already mentioned before, CpG sites are not completely 100% methylated or not methylated. There will be always a portion of uncut DNA, which can lead to unspecific amplification, especially in mixtures with a high DNA input. Extreme high DNA input from female DNA can lead to sufficient remains of uncut DNA, not only because of incomplete digestion but also because of increasing DNA amount of the methylated portions of the loci. Furthermore, Lin et al. announced this risk and stated that their assay could be improved by incorporating Y-chromosomal markers, which they could not find in their studies. Therefore, we think it is better to use digestion controls which reflect better the undigested part of the methylated DNA of the markers. Our approach might be more conservative and therefore our resolution for our autosomal markers is smaller. However, in any case, resolving very unbalanced mixtures of semen/female bf can result in unclear TSMPs. In this context, Y-chromosomal bf markers are very helpful for clear interpretations.

Marker stability is also important for analysis of forensic samples, because traces can be exposed to different environmental conditions for a long time. Notably, CpG methylations appear to be very robust and are detected even in formalin-fixed samples [28]. Forat et al. [29] found that their methylation markers tested using a bisulfite assay were still stable after 6 months of outdoor storage. They reported that all markers remained unchanged under dry conditions. Under humid conditions, they detected changes in methylation, but this could have also been due to DNA degradation and the less sensitive bisulfite treatment [30]. In our study, we tested long-term stored GEDNAP samples from the last 8 years, which were extracted following the same protocol as used for our routine forensic traces. Importantly, the long-term stored samples did not show differences from fresh DNA extracts, supporting that methylation markers are very stable. This is of great interest because in many instances, questions regarding bf type arise late in an investigation, often not until the trial itself. Protein-based methods and RNA analysis require extra material or extra preparation steps for DNA extraction, which means they cannot be applied retrospectively. In these cases, it would be still possible to perform MSRE/MDRE-PCR on stored DNA samples from the traces of interest for court. The fact that MSRE/MDRE can be performed directly from the routinely produced DNA extract also reduces material consumption, and broadens the method to a greater number of crimes (volume crimes).

## Conclusions

In summary, our three MSRE/MDRE assays enabled identification of the bfs semen and buccal mucosa. However, we observed reduced identification power for vaginal secretion, saliva, and blood, which was mainly due to an insufficient number of markers. In further research, we plan to expand our multiplex assays with further markers for blood and vaginal secretion. It would also be desirable to extend these assays with markers for additional bfs, such as menstrual blood or skin, and to make increased efforts towards the validation and standardization of MSRE/MDRE-PCR. As the methylome becomes increasingly important for forensic DNA analysis, it will be important to establish a methylation database for the forensic community, which introduces a common nomenclature and covers bf of forensic interest (e.g., to differentiate between buccal mucosa and saliva). Overall, in our present study we demonstrated the power of MSRE/MDRE-PCR for bf detection, pointed out both the advantages and challenges of this method, and highlighted the best solutions for assay design.

### Disadvantages

- Limited assay design due to the dependence on cutting sites and finding specific markers for complex bfs
- High dependency on PCR efficiency, which hinders method transfer between laboratories

### Solutions

- Expanded loci search of other databases and technical platforms [31].
- Use of combinations of different methylation-sensitive and -dependent enzymes.
- Design of TSMPs with several markers for each bf, and also markers with different information load (e.g., non-blood, non-blood + non-saliva, Y-chromosomal markers).
- High standardization of assays, using the same PCR components and conditions.

### Advantages

- Economical and fast methods, which can be conducted in any standard forensic laboratory, without extra equipment, on the same platform as used for STR typing.
- Simultaneous amplification of bf and STR markers allows direct correlation of the STR profile with the bf.
- High sensitivity of the assay, and high stability of the markers, enables the analysis of samples with low DNA content or degraded DNA, and even long-term stored DNA samples.
- No extra DNA extraction is necessary, and DNA analysis can be performed on long-term stored DNA extracts. Therefore, it can be applied in cases where questions about DNA arise later in court and material of the trace has been saved.
- Combination of autosomal and Y-chromosomal markers allows the resolution of male/female mixtures and can provide more evidence of whether body fluid derives from a male or female person.

### Supplementary Information

Below is the link to the electronic supplementary material.

Supplementary file1 (DOCX 294 KB)

Supplementary file2. marker details. (XLSX 85 KB)

Supplementary file3. Sequences of autosomal markers (PDF 1152 KB)

Supplementary file4. Sequences of Y-chromosomal markers (PDF 823 KB)

## Data Availability

The data presented in this article are available in the article, supplementary material, and from the corresponding author on reasonable request.
