# Supplementary material for: Expanding the scope of methylation-sensitive restriction enzyme (MSRE) PCR for forensic identification of body fluids through the novel use of methylation-dependent restriction enzymes (MDRE) and the combination of autosomal and Y-chromosomal markers
Source: Int J Legal Med. 2023 Oct 24;138(2):375–93. doi: 10.1007/s00414-023-03097-9 (PMC10861701; doi:10.1007/s00414-023-03097-9)
Supplement: Supplementary file 1 — Supplementary file1 (DOCX 294 KB) [file 414_2023_3097_MOESM1_ESM.docx]

Expanding the scope of methylation-sensitive restriction enzyme (MSRE) PCR for forensic identification of body fluids through the novel use of methylation-dependent restriction enzymes (MDRE) and the combination of autosomal and Y-chromosomal markers

**Online Resource 1**

**a. GEDNAP samples**

**b. Search and selection of autosomal and Y-chromosomal CpG loci**

Literature search


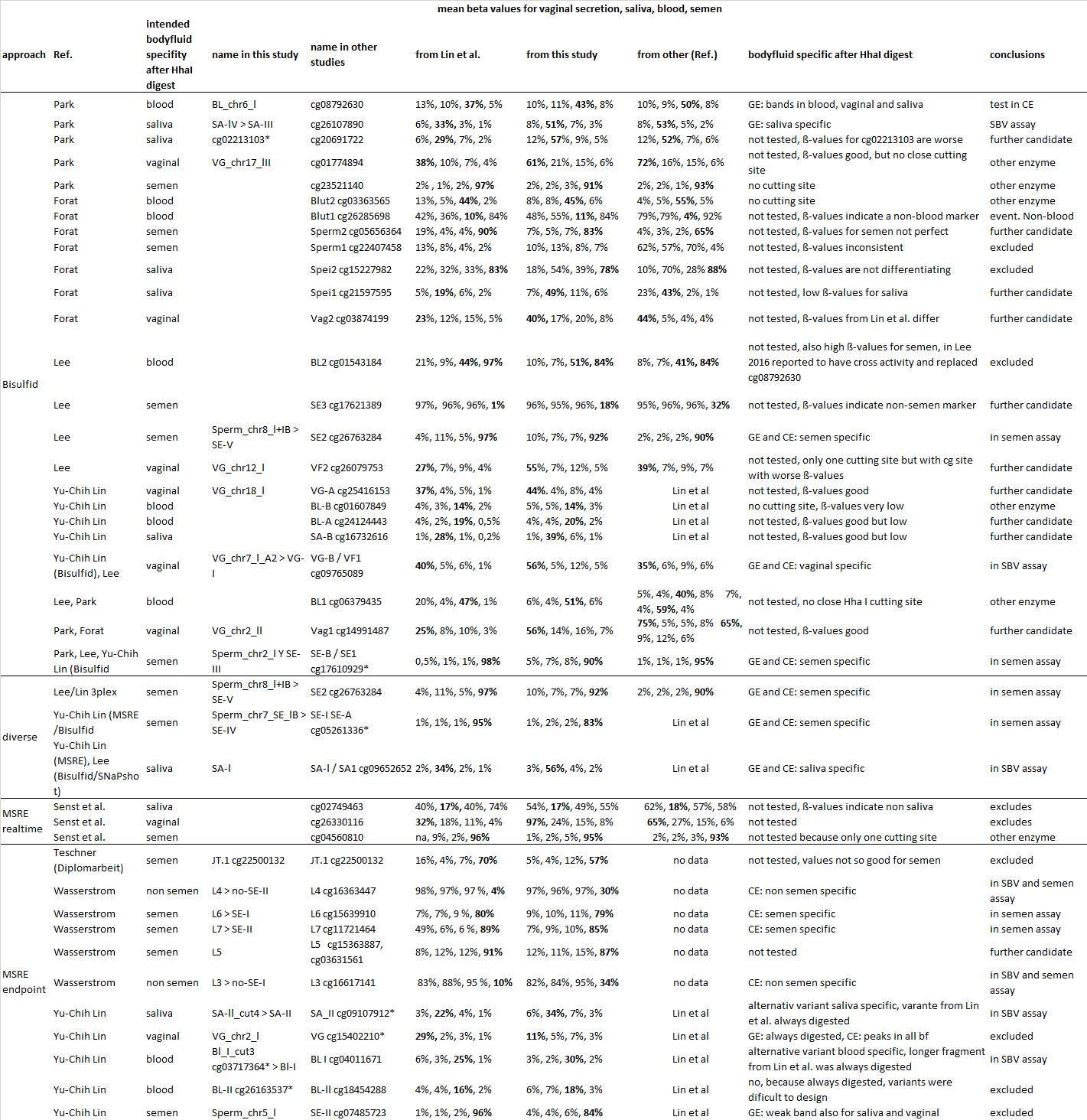


**Table 1 shows a summary of CG sites from forensic literature for bf identification. Given ß-values are from indicated references or one dataset (more information in supplement 2). Values in bold indicate concordant ß-values for differentiating one bf between the datasets. Marker names in bold indicate the marker names which are used for the selected marker in the MSRE/MDRE assay. The original names of the markers in this study were chosen after primer design, the different variants of one locus, the intended tissue specificity and sometimes on the naming from other publication. The original names of the markers for our three multiplex assays were renamed for this publication for easier reading, e.g. the locus gc05261336 was named in Lin et al. SE-I [7] and in Lin et al. SE-A [11]. In our study, we designed new primers for this locus for different variants and named them Sperm_chr7_xxx (x for different variants) and for this paper we renamed it to SE-IV. In this study we use the name SE-I for the marker L6 from Wasserstrom [6] and therefore should be not confused SE-I from Lin et al [7]. GE gel-electrophoresis, CE capillary-electrophoresis, DC digestion control, *CG site is located on a HhaI cutting site. More information about each CG site are given in supplement "autosmal markers".**

Database search

We used the dataset from Lin et al. [7], which comprises five studies with samples from individuals from four different countries in Europe, including vaginal secretion (n = 20), saliva (n = 22), blood (n = 11), and semen (n = 8) (Supplement 2) (Table 2). We also created our own dataset, comprising samples from Korean individuals, including vaginal secretion (n = 9), saliva (n = 16), blood (n = 18), and semen (n = 12) (Table 3). Comparison of the ß-values from the dataset of Lin et al. [7] with our own established dataset revealed similar values for most of the markers, or values that were in concordance with the tissues with the highest values. We found completely different values in only some cases, especially for vaginal secretion and Y-chromosomal markers.

**Table 2: Composition of dataset from Lin et al. 2016 for methylation values from different tissues of NCBI platform GPL13534: GSE numbers are listed under each platform (GPL number). The GSE number is the accession number of the series, i.e. the paper/work that used the respective platform. GSM numbers are listed for each GSE number. The GSM number is an access number for each probe. The individual samples of the respective series can be found under this number.**

**Table 3: Composition of own constructed dataset for methylation values from different tissues of NCBI platform GPL13534: GSE numbers are listed under each platform (GPL number). The GSE number is the accession number of the series, i.e. the paper/work that used the respective platform. GSM numbers are listed for each GSE number. The GSM number is an access number for each probe. The individual samples of the respective series can be found under this number.**

Candidate CpG loci were selected from the 450K array datasets by filtering the ß-values for tissue-specific methylation patterns (TSMPs), depending on the used restriction enzyme. In the case of the methylation-sensitive enzymes *HhaI* and *SmaI*, we filtered for CpG sites that showed high ß-values in one bf and low methylation in all other bfs. In the case of the methylation-dependent enzyme GlaI, we filtered for the opposite conditions. The 450K array datasets were filtered for CpG sites exhibiting the highest possible differences in their ß-values between the body fluid of interest and the other bfs. All potential CpG sites were examined in greater detail by checking about 600–800 bp around the marker for the availability of cutting sites and for additional CpG sites from the 450K array (Supplements 3 and 4). 37 CpG markers had to be excluded because they did not have a sufficiently nearby cutting site for the intended enzyme. In some cases, a CpG site was excluded if other nearby CpG sites exhibited different or worse ß-values. CpG sites were also excluded if the number of cutting sites did not correspond to the methylation values—for example, if the methylation values were about 90% for semen and 20% for other bfs, we would want to include more than one or two cutting sites. On the other hand, we sometimes had to exclude markers because there were too many nearby cutting sites and low ß-values.

**c. More detailed results of loci search**

A loci search for blood markers did not reveal any CpG site having high ß-values (>60%) for blood and low ß-values for other bf. In particular, saliva values were always high. Therefore, we had to widen the search to include ß-values below 60%. For example, we tested the CpG site cg02345886, which showed ß-values of ~59% for blood, but also of ~20% for the other bfs. Our first test of cg02345886 by gel electrophoresis showed additional nonspecific bands for saliva and vaginal secretions, which could have been caused by the moderately high ß-values (~20%) for these bfs. In this case, the ~30% methylation differences were not sufficient for clear bf differentiation. Therefore, we further decreased the ß-values for blood to ~20%, which is similar to the values for markers BL-I and BL-II reported by Lin et al. [7]. This enabled the identification of one additional blood-specific marker, cg19637387. However, we did not include this marker in our final multiplex MSRE-PCR, because its PCR product appeared to be 20 bp longer (checked by Sanger sequencing, data not shown) than predicted based on the NCBI reference sequence, leaving us uncertain about its origin. We also tested new CpG sites for the differentiation of vaginal secretion, which showed bigger methylation differences between the bfs, compared to the CpG sites for blood. However, our pre-testing of potential vaginal secretion markers yielded the identification of only one new vaginal secretion-specific marker, VG chr7_II. However, further validation tests showed that the VG chr7_II marker also appeared for saliva, which can be explained by the moderately increased ß-values (~30%) for saliva. Comparison of the ß-values from the dataset of Lin et al. [7] with our own established dataset revealed similar values for most of the markers, or values that were in concordance with the tissues with the highest values. We found completely different values in only some cases, especially for vaginal secretion and Y-chromosomal markers.

**Table 4: Summary of loci search and tested marker**

**d. false negative/ positive rates between fresh and GEDNAP samples**

fp, false positive; fn, false negative; no, no fp or fn were observed, n.d. no data

**e. Boxplot saliva-blood-vaginal and semen test-sets from tested samples**


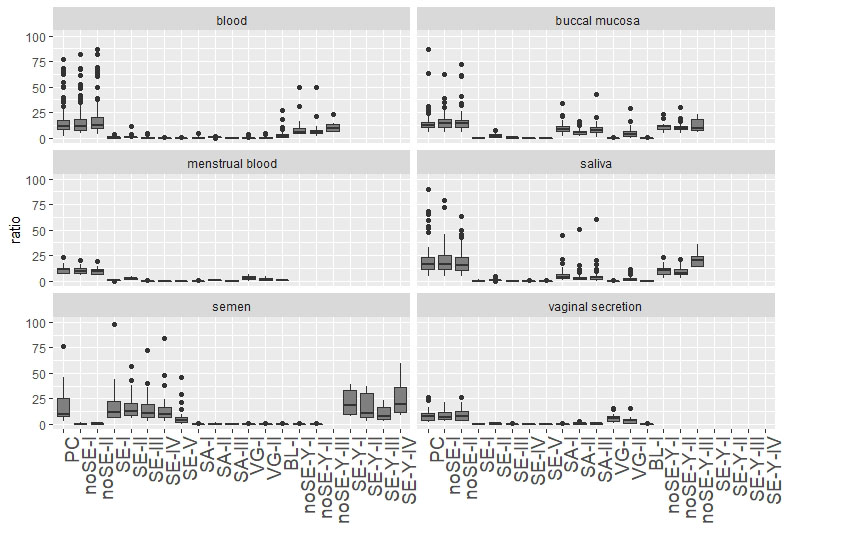


**Boxplot was drawn with ggplot function geom boxplot(). Because of complete digest of DC, for some samples very high ratios over 100 were obtained. For better illustration Y axis was put to 100 and some outliers are not displayed. Number of samples: 10 semen, 49 buccal mucosa, 49 saliva, 46 blood, 7 menstrual blood, and 17 vaginal secretion samples from own sample collection, as well as 38 blood and 10 semen GEDNAP samples. The Y-semen test-set was applied to a smaller assortment of 6 samples from own collection for each body fluid (bf)**
