# Supplementary material for: Expanding the scope of methylation-sensitive restriction enzyme (MSRE) PCR for forensic identification of body fluids through the novel use of methylation-dependent restriction enzymes (MDRE) and the combination of autosomal and Y-chromosomal markers
Source: Int J Legal Med. 2023 Oct 24;138(2):375–93. doi: 10.1007/s00414-023-03097-9 (PMC10861701; doi:10.1007/s00414-023-03097-9)
Supplement: Supplementary file 3 — Supplementary file3. Sequences of autosomal markers (PDF 1152 KB) [file 414_2023_3097_MOESM3_ESM.pdf]

Inhaltsverzeichnis

Vaginal secretion Marker 4

VG_1_l cg13356427.... 4
VG_1_ll cg09809932.... 4
VG_chr2_l cg15402210 (Lin et al. 2016) 4
VG_5_l cg04541368.... 5
VG_7_ll cg18502142 5
VG_10_l Cg26439963 5
VG_chr14_l cg09197895.... 6
VG_17_l cg03656099 6
VG_chr22_l cg14703829.... 7
VG_chr7_l Cg09765089 (Lin et al. 2016).... 7
VG_chr2_II cg14991487 (Park 2014, Forat 2016) not tested.... 8
VG_chr18_I Cg25416153 Lin 2016 not tested 8
VG_chr17_II cg27215100 not tested 8
VG_chr12_I Cg26079753 (Lee et al. 2015) not tested 9
VG_chr17_III Cg01774894 (Park et al. 2014) not tested 9
Cg03874199 (Forat et al. 2016) not tested 10
cg12020396 not tested 10
cg20166714 not tested 10
cg20090283 not tested 10
cg22052566 not tested 10

Saliva Marker.... 10

SA-I cg09652652 (Lin et al. 2016) 10
SA-II cg09107912 (Lin et al. 2016) 11
SA-IV cg26107890 (Park et al. 2014).... 12
SA_chr16_I cg10096948 not tested 12
SA_chr1_I cg06936779 not tested.... 12
SA_chr17_I cg15825321 not tested 13
Cg16732616 (Lin et al. 2016) not tested 13
Cg20691722 cg02213103 (Park et al. 2014) not tested.... 13
Cg15227982 (Forat et al. 2016) not tested 14
Cg21597595 (Forat et al. 2016) not tested 14

Blood Marker.... 14

BL-I Cg04011671 (Lin et al. 2016).... 14
BL_chr1_l cg02431672 14
BL_chr1_lll cg19637387 15
BL_chr2_I cg02345886 15
BL_chr2_ll Cg06645778 15
BL_chr6_l Cg08792630 (Park et al. 2014).... 16
BL_chr11_l cg04434593.... 16
BL_chr12_l cg22249612 16
BL_chr14_l cg23051349 17
BL_chr16_l cg06329093 17
BL_chr17_l cg05164926 17
BL_chr22_l cg19343611 18
BL_II Cg18454288 (Lin et al. 2016) 18

cg00340958 no HhaI cutting site 18
cg06379435 (Lee et al. 2015 / Park etal. 2014) no HhaI cutting site 19
cg01607849 (Lin et al. 2016) not HhaI cutting site 19
Cg03363565 (Forat et al. 2016) no HhaI cutting site 19
cg24595580 no HhaI cutting site 19
cg04658021 no HhaI cutting site 20
cg20155875 no cutting site 20
cg27598107 no HhaI cutting site 20
cg01092213 no HhaI cutting site 20
cg19238415 no HhaI cutting site 20
cg18464274 no HhaI cutting site 21
cg01840419 no HhaI cutting site 21
cg04111435 no HhaI cutting site 21
cg25784219 no HhaI cutting site 21
cg25365958 no HhaI cutting site 21
cg07152894 no HhaI cutting site 22
Cg24124443 Lin et al. 2016 not tested 22
Cg01543184 (Lee 2015) not tested 22
Cg26285698 (Forat et al. 2016) not tested 22
cg19008649 BL_chr2_III not tested 22
cg00664416 BL_Chr1_II not tested 23
cg14179389 BL_chr1_IV not tested 23
cg10573476 BL_chr11_II not tested 23
cg24749947 BL_chr2_IV not tested 24
cg02676865 BL_chr10_I not tested 24
cg20225915 BL_chr11_III not tested 25
cg00113675 not tested 25
cg17156633 not tested 25
cg14263391 not tested 26
cg25467652 not tested 26
cg17452301 not tested 26
cg27518898 not tested 27
cg27424995 not tested 27
cg01022567 not tested 27
cg05127217 not tested 27
cg03784054 not tested 28

Semen marker 28

Sperm_chr2_l cg17610929 (Park, Lee, Lin) 28
Sperm_chr5_l Cg07485723 (Lin 2016) 29
Sperm_chr7_l cg05261336 (Lin 2016) 29
Sperm_chr8_I Cg26763284 (Lee 2015) 29
JT.1 (Muc1.1) (Teschner et al.) 30
L6 (Wasserstrom et al.) 30
L7 (Wasserstrom et al). 30
Cg23521140 (Park et al. 2014) no HhaI cutting site 31
cg23324953 no HhaI cutting site 31
L5 (Wasserstrom et al.) not tested 31
Cg05656364 (Forat 2016) not tested 32
cg14061378 not tested 32
cg02543772 not tested 32

cg04560810 not tested 32
Non Semen Marker 32
Non semen marker L3 (Wasserstrom et al). 32
Non semen marker L4 (Wasserstrom et al). 33
Cg17621389 (Lee et al. 2015) 33
Cg22407458 (Forat et al. 2016)........ 33
Controls........ 33
PCR control 1 (L1) (Wasserstrom et al). 33
PCR control 2 (L8) (Wasserstrom et al.)........ 34
DC (Digestion control L2) (Wasserstrom et al.)........ 34
PC (Lin et al.)........34
DC (Lin et al.)........35

Vaginal secretion marker

VG_1_l cg13356427

CCCCTTGCATGGGTGACCTGGCCTCTGCCTGGTGCCTGGCCCCACCCTTTCTGCCAGCCTGGGTATCTGGCCCCAGACTTCACCGGGTCTGCCCCCCTCCCCACT
GCCTCAGGAAAGAGGAGGAGCGACAGAAGCAGGAGGAGCTGCGGCGGGAGAAGGAACAGTCAGAGAAGCTGCGGACGCTGGGCTACGATGAGAGCAAGC
TGGCGCCCTGGCAGCGACAGGTCATCCTGAAGAAGGGGGACATCGCTAAGTACTAGAGGCCGCAGACTCCTGTCCGCAGCCTCGCAGCTCCGTGGGGCCCTCC
GCCCCAGCCCCAGCCAGCCAGGCCCTGGTGGAAAGGCTGGGAGCCGCACAGCCCTCCCCTCCTGCGCTGGAAACCCTCCCTGACCCCCACCCTGGCCCCCCGTA
TCCCCAGCCCTTGGCAACACTGGAGTGCACACGCCGCCACGGTTGCCCAGAAAAAGTGCCCAAGCTGCTGACGCAAACAACAACAAATGCTGCTTATTTGCATG
CCGACTTACATATATTTGCATGTTCGTTGACTATCAAAGAGTGCAGAGCTCTCCCCAGCCCCGTGGGTGGTGACTTTGTTTTCCTGCGGGGCTCAGCCCCCTCCA
GGATGCAGCCCCCTCCCCCGCACCCCGGAACCGGCGTCGCTGGCGCATCCTGGGTGGAGGCAGGCCCCGAGCTCGGGGAAG

cg13356427 (Blood 2%, saliva10%, sperm1%, cervix 60%)

Vaginal chr1 forward: TM: 64°C, Länge: 20 bp

Vaginal chr1 reverse: TM: 68°C, Länge: 19 bp

VG_1_ll cg09809932

CCGAGCAGCTGGCGCGCCGGCCGCCCCTCTGCACGAAGCTGCGCGGCGTCCAGGACTACCTCGACCTGCGCAAGGAGCGCATCGTTTACCTCTTCCTGGAGCA
CTGGCGCCGCTGGGCCTGCCGCGGACCAGGCCGCCGCGCCCAGGCGCGCCTACGCGGACTGCTGCCCCGCGTGGCGGCTGCCGGTGCTGGCCCGGGGCTGG
AGGCCACGGACGCCCCCCGGCTGCCGGCGAGCAACAGCGAGGCCCACAGCCCCGACGAACGGCTGCGGCAGCTGCTGAGGCAGCGGCAGGCGGTGGGCAA
GCTGCTGGGCCACTGGCGGAGCCTGCTGCGTCGCGTGCCGGCAAGCCCGGGCCTGGCGCACGGCCTGTATTGGCCCCAGCACTTCCTGCCGCCCCTGGACGG
CGGCGCACCCCCGCACTACGACAGCCTCACGCTCGACCTCTTCATGCTCGGCTACTTCCAGCTACTCGAGATGGGCCTGAGCCGCGAGGAGCGCAAGTTCCGCC
ACCTACTGTGCTACGAGATGTTCGACCGACTGGGCAGCCACCCGTGGGAGCGCATCCGCCTCTTCCACCGCGTGGTGCTGGAGGAGGTGGAGGCCGGCCGGC
GCGGCTGGAGCGACGGCTTCGAGGACCTCAGGCACCGGTTCTTCGGAAACGGCCTGGAGGCTGAGCCGGCCCCCGAAGAACAGGCGAAG

cg09809932 (Vaginal 60% +-12, Saliva 4% +-2, Blood 10%+-4, Semen 1 +-0)!!!!!!!!!!!!!!!!
cg00833822 (Vaginal 53%, Saliva 27%, Blood 33%, Semen 10)
cg26419880 (Vaginal 50%, Saliva 5%, Blood 8%, Semen 1)
cg12143651 (Vaginal 40%, Saliva 23%, Blood 27%, Semen 8)

Vaginal_chr1_forA: TM: 64°C, Länge: 18 bp

Vaginal_chr1_revA: TM: 64°C, Länge: 17 bp

Vaginal_chr1_forB: TM: 66°C, Läng:e 19 bp

Vaginal_chr1_revB: TM: 64°C, Länge: 19 bp

VG_chr2_l cg15402210 (Lin 2016)

ATTCGAGGAACCTTCACTCCCGAGGGGCCTCGCCGAAAAGCAGCTCCTTCGCTCCCGAAGCCGCTCCCAATCGGCCAACTGACAACCCTTCCCTCGCCCCAGATT
TGCTAGGATGGTCTGGGACCGCCGGGCGGCCCCTCTACCTGCCCCTTCAGCTTTAGTCCCGAGCCTCTCGGGGTTTCAATTTGGGCTGCTCGGTGGCGCCCGAA
CGCCCCTCAGCGGCGGTGGAGCGTGGACTCCGGCGCCTGGCGGGGTAAGGCCCGCGGCAAGGGCGCTGCATCCGGACATCGCTGCGTGGTGCCCAGACGCT
GGCTCCCGAGAACGGGCACGAGCCCAGCACGCCGGAGGACCCGGAACTCGAAAGCCCGCGCTGCGGGAAAAGAGACTATCGGTCCGAGGGTCGCAGAGGA
GCAGGGGCGTTGGCAGCGGGCTTGCTTGGGCACAGTCTGCGGGCCGGAGCAGAACTTCCGCCCCTGGTCAGATCCCTCGGCCTCAGATCCA

cg15402210 (Vaginal 30%, Sperm 1%, Blood 2%, Saliva 2%)

VG_chr2_I_forw: TM: 66°C, Länge: 19 bp

VG_chr2_I_Rev TM: 66°C, Länge: 20 bp

VG2Icut3var1f TM:66°C, Länge: 21 bp

VG2Icut3var2f: TM: 66°C, Länge: 21 bp

VG_2_I_cut2_f: TM: 64°C, Länge: 18 bp

VG_5_l   cg04541368

GGATGCAGGATATGGAATTAGGGAATTCGGGACGGAAACGTAGCGTTTAGGACAAAGGGGATTCACTGGGGACTTCCGCGGCCTTTGCCCAGATGCGCCTTT
CCACCCTTTCTCACTCCAGTCACTCCCGAGGACTTGGGGCACGACTATCACACGCAACTGGGAGATCCTGGAAGACGGAGGAAAAACGAACAAGGGGACATG
GCCCTCACTGCAGTGACAGGGCTTTCCTTCAGTCAGTGGCCACAATAAATTTAACCAAGGCTAAAGGAGATTAATTTCCCAGCATAATCCAATTAAAAGATTTCT
AAAGTAATCTTTTGCGAAAATGAAAAGTGCGCGACTAAAGAGGGGACTGGTTTTGATGACAGTGATTTATCGCCTCAGCACAGCACGCACGGGACGCTGTCTC
TCCAAGCGATTTGACCAGAGCATCCCGTCCTCGCCTCCTGTCCAAACCTTCCTCTTCCTGAAAGACAGCCATCTATCACGCCAACCTGGGCAGGAGAGAATGTGC
AAGGGGCTGGGGCGGCTTACAAGCACCACAGACCTTTTTAAGTGCTATTAGATTTATGGTCTCTTTTTCGACACCCATCCAGAGTAATTAGCACATATGTTCTAA
ATAGATGATAGTTTTGTGAGCAATAAAGCAATTACCCATCGTTGGAGCTGACAGTTCCTCCAACTAAACTCCAACCAGC

cg04541368 (Blood 3%, saliva8%, sperm1%, cervix 66%)
cg06668065 (Vaginal 56%, Sperm 4%, Blood 6%, Saliva7 %)
cg15143788 (Vaginal 56%, Sperm 6%, Blood 14%, Saliva 19%)

Vaginal chr5 forward: TM: 66°C, Länge: 21 bp

Vaginal chr5 reverse: TM: 66°C, Länge: 19 bp

VG_7_ll   cg18502142

AAGATGAAGTACGGGTGGTGGCTGGAGAGCTGGGACTGGAAGGTGGGGCTGGGGGAGACGGGTAAGCAGCTTGCTTTGGTGAGGAGCTCCCACGCTGAGA
ACGGTGGGAGGAGGGAATGATGGGGAAACCTTTGTTTAATGAGGAAACCTAATTATTGCGCATCTGGAAGTCTTAGTCCCTACTACCAGACGAAGGCGGGTAC
CTGAGGGATGCAGTGCGCATGCCCGAGCCGTCGGATTTGCATGATAAGGCTCGCACGTGAGCCAACTCCGGCGCAGCCCAGGCTGTGCCGCGGCCGGCCGCC
AATGGAATCTTGCTCCTACTGCGCCTCCAGGTCACATTGACATCTCGGTTTCCCCACAATAGGCCTTTAAAACCCACTTTAAAAATAAAAAAGTGTTTTAGTATTG
ATTCTCGCCTAGACACGGAAGATGTCTGTAGGAAACTGACACGGTTTAGAACTACAACTACATTACTACAAGCAAGGGTTAAAGTATTCAGAATTTAAGAAAAA
GTTAGAAGGCCGGAGAGAGGGGGCTGTTTAAAGAGCTCTGGCGGGAAGTCAAGGAATCCTGGGACTGCTGCCTTCGGAGCCCTGAG

cg18502142 Vaginal 75% (19%, min bei 18%), Saliva 28% (14%, max bei 82%), Blood 14% (4%, max bei 21%), Semen 2% (1%)
cg09803262 Vaginal 68% (15%), Saliva 32% (7%), Blood 27% (5%), Semen 2% (1%)

VG_chr7_ll_for TM: 64°C, GC%: 60%, Länge: 20 bp

VG_chr7_ll_rev TM: 62°C, GC%:55%, Länge 20 bp

VG_chr7_ll_for_alte TM:62°C, GC%: 50%, Länge 20 bp

VG_10_l   Cg26439963

GTCCGCCCTGGCTTGCAGGCGTCTGATCCCCACTCCCCGACCCTCCCGCAGGGAGCGAGTTCTCCGGCAACCCGTACAGCCACCCCCAGTACACGGCCTACAAC
GAGGCTTGGAGATTCAGCAACCCCGCCTTACTAAGTGAGTACGCCACCTGGCTGGCCGGCGGCTCAGCGCGGCCGCGCGGCTTCTGGGCACGGTCCCACTCCC
GGCGACCCGACCTCTGGGGACCCGGCCGGGCCAGGGGGACAGGCTTGTTAGTGCAGCACTGAGGCCCGGGGACCCCTCGAGGACGCCCGCACCTCCTGCCCT
CCCGTGCCCTCCCTGGTCGCTCGAAGGCTCTGCGCCGCCCTCGCCCTCGGATCCCCTGGAGGGTGCGCAGCCGGGCGTCACCAGATCGGGTCCCTCTCATGCCC
CGTGAACGCAACTATTCTCCGGGGCAACTGGCTCCACTGCCCAGCCAAGGTCTCCCAGTCCGGATCCCGCTGGACCCCAGCCAGGGGAGGTCTTTTCTGTGCTT
TTCTTTCCTTTTTTGTTCTCCTGTTTGTCCTCTCACCCAGCCCATTCTTCTCCTGTGTTAACTTCCAGGTTCCCCTTATTATTATAGTGCCGCCCCCCGGGGCTCCGCC
CCTGCCGCTGCTGCCGCTGCCTATGACCGCCACTAGTTACCGCGGGGACCACATCAAGCTTCAGGCCGACAGCTTCGGCCTCCACATCGTCCCCGTCTGACCCCA
CCCCGGAGGGAGGGAGGACC

Cg26439963 (Blood 3%, saliva3%, sperm2%, cervix 60%)
cg25531836 (Vaginal 38%, Sperm 7,6%, Blood 9%, Saliva 7%)
cg04943368 (Vaginal 53%, Sperm 10%, Blood 14%, Saliva 14%)

Vaginal chr10 forward TM: 66°C, Länge: 19 bp

Vaginal chr10l reverse TM: 66°C, Länge: 24 bp

VG_chr14_l cg09197895

AAGGATGTGCAAAATGTTCCGCCTGCGAAGGTTCCGCGGTGGCGGGGACACTGCTCCGGCTCCGCTCCCGCCCGCCCGAGCGCTCGGATGGGGCCGCCTCTGC
ACTGCGTGGCCACAGGCGCGGCCCGGCTGCCCACGGGCGCCCTTTGCAGCTGCTGCCCCCTGGCGGCCGCGGGCGGCTACTAGCGGGAAAGCGAAACCCGCC
CGGTCCATTCAAGCCCCGCTGCCTGGCGCCCTCTAGGGTCGTTCTTGGGAACGGGCGGACCTTTCGTCAACACTTTGCCTGCAAGATCCCCCATTGGGGGAACC
GAGGAGGAAGTTAAAGGAAGATGTGTGTTTTTGAGCGCTGCTTTGTGCCAGGCTCATCTTAGGTGTGGGACGTGTACTATCTGAATTAATACCCCACCAGGCCT
GTGGGACAGTCACTGTCACCATTCGCAAATTATGGATGAAGAAAGGAGGTACCAAGTGGTGGTATCACCTGTCCATAGTGAGCTGTCCCTCAGGAGGGTGGCC
GCCCCACGCGACCATTCAGTGTGCTCTCATTCACACGTTCATTACCCAAGAGCACTGCTAGCACTGGGGGCTGGCTCACTCCTGC

cg08727193 Vaginal 46% (21%, min: 5%), Saliva 4% (2%, max: 8%), Blood 5% (2%, max: 7%), Semen 3% (1%)

cg09197895 Vaginal 30% (15%, min: 1%, max: 49%), Saliva 1% (1%, max: 2%), Blood 1% (1%, max:2%), Semen 0,4% (0,3%, max: 1%)

cg18985581 kein Vaginal?, Saliva 1% (1%), Blood 1% (1%), Semen 0,3% (0,2%)

cg22796749 Vaginal 45% (18%), Saliva 1% (1%), Blood 2% (1%), Semen 1% (0,4%)

VG_chr14_l_for TM: 64°C, Länge: 21 bp

VG_chr14_l_rev TM: 64°C, Länge: 21 bp

VG_chr14_l_for_alter:
ACTTTGCCTGCAAGATCCCC
VG_chr14_l_rev_alter
ACAGGCCTGGTGGGGTATTA

VG_17_l cg03656099

CGTGTGTGCGAGCCCGTGAGTGTGATTTGGCTTTGTGTGTACCTGCAGCCTTGCGTGTGAACTGCAGTCTCTGTTCGCGCCTGCTCGGGTGCGGAGTCGTTTAA
CTTGCGCAAGCCCGCGGGCGGGTGTGCATGCTTGGGCTGGTTCCGACACTGTGGCACTGCTTTAGGCCTGTGTGGGGCTGTCTAGGCCTGTCTAGGGATAATT
CCTCGAATGAAGCTTTTGGAGGACAGACCCCGTCGCTTTCCGCGAGGCCCTCCCACCGGCCTCAGCTCGGGGCGTCCCATCCCAGGCGGGTGGGTACCAAGTG
GACGCTCCCCGGGTCTTCCCTCTGCGGCCAGCACCCGCTGACCTCGGGGTGCCACCGACCACGCTGGAAGAGCCACGGCCTGACTTAGCGCCGCCCCCTTGGTC
CCCGCAGATCACACAGCTGAAGATCGACAACAACCCGTTTGCCAAGGGCTTCCGGGACACCGGGAACGGCCGGCGGGAGAAAAGGTGAGAGGCCGAGGACA
GCAGCCCTGTGGCGGGTGCCCGCGGGAGCAGCGAGCAGGAGGGATGGAGGGATGGGCTCCCCGCTGACCTGGTTCATCCGCTCATCCCCAGGAAGCAGCTG
ACGCTGCCGTCTCTACGCTTGTACGAGGAGCACTGCAAACCCGAGCGCGATGGCGCGGAGTCAGACGCCTCGTCGTGCGACCCTCCCCCCGCGCGGGAACCAC
CCACCTCCCCGGGCGCAGCGCCCAGTCCGCTGCGCCTGCACCGGGCCCGAGGTGAGGGTCGGACCGGAGGAGGGACAGGGAGGTGGCGGCGGGGGGTCCT
CAGGTCGCTGGGCTGGTCTTTTGCTGAGCCACCCGCTAACCTGAAAGGCCAGGAAGGAAACGTCGGCGAGTGTCTGGGATGGGGTTTCCGTCCCGGGACTCCC
CTACGAGGGCGGTCCCCGGTAGCCAGAAGATCCGGCCGGACTCCGAGCCTGGCCCCTTGGGCGCCGTGTAATTCTATAGCTGTAGGGTTTCCTCTCCAGGGCT
GGGTTCCTTCCACTGTAAATCTGGGGTCTTGGATTAGGTGACCTGCAGCTGCCCTTTGCCCGACTCCAGCAGCTCCTCCGACTCGGCCTCCCCGAGAGCAGCCCT
TCCCGAGTGTCCCTGATCCTGCTCGTCGGCCTCCGCCCAGGCCCCTGTAATCCGCGCGCCCTCTCCCCGCAGCTGAGGAGAAGTCGTGCGCCGCGGACAGCGAC
CCGGAGCCTGAGCGGTTGAGCGAGGAGCGTGCGGGGGCGCCGCTAGGCCGCAGCCCGGCTCCAGACAGCGCCAGCCCCACTCGCTTGACCGAACCCGAGCG
CGCCCGGGAGCGGCGTAGTCCCGAGAGGGGCAAGGAGCCGGCCGAGAGCGGCGGGGACGGCCCGTTCGGCCTGAGGAGCCTGGAGAAGGAGCGCGCCGA
AGCTCGGAGGAAGGACGAGGGGCGCAAGGAGGCGGCCGAGGGCAAGGAGCAGGGCCTGGCGCCGCT

cg03656099 (Vaginal 52% +-13, Saliva 3% +-2, Blood 3%+-1, Semen 1 +-1)

cg22675937 (Vaginal 27%, Saliva 10%, Blood 16%, Semen 2)
cg12999453 (Vaginal 58%, Saliva 8%, Blood 8%, Semen 3)

cg21201099 (Vaginal 48%, Saliva 18%, Blood 15%, Semen 11)

Vaginal c17_I_cut1+2_for Tm 64°C, 20 bp

Vaginal c17_I_cut1+2_rev Tm 66°C, 19 bp

Vaginal_chr17_cut2_for Tm 62, 20 bp

Vaginal_chr17_cut2_rev Tm 64, 20 bp

VG_chr22_l cg14703829

GGACCGCCGGCGTCCGTGGGCTCCAAGAGGCCGGGGCCGCGGCCCCGCGCTCGGGCCCTGGGCGGCGAGGGCGCAGCGAGGGCTGTCGCCTCCTCGGGCA
GCCCGGGGGGCCGCGGCGTTGGGTCGCGGGTCCGGGGGCTGCCGTGTTCGACCGTATGCGCCGATTTGTCGCTGGGCGTCCGTTTCCTCTTGCTGGGGCTGG
GCGCGGCCTCGGAGCCTGGCGGCGGTGGTGAGGGCGCACGGCCAGCTGCAGCGGCGCTGTGCTTGCCGTGGATCCAGGACACCTTAGGCTTGGTGGCGGGG
TCAGGTGGCGGCGGTTTCCTGCGCTCGGGCGATGGCGACAGCGACAGGCCCCCAATCTCGCCCCGGGCCCGGGCCGGCCGGGCCTCGCGTCGGGCCACGCGC
GCGTACAGAGCCCCTCCGGGCCCCTCCACGCTGGACGCCGACCGCTCGCTGTCGGAGGACGCGGGGAGGGGTATCGCCTCCTCGGCGGAGGCTGGCGTGGTC
AAGGGCACAGGGGACGGCGCCGGCGCCTCGGCAGGGACAGTGGGGACTTCGGGGTCCCGGCTCTCCGCTGGTGCCTCTGGCAAGGGAAGAGCAG

cg14703829 Vaginal 5% (1%), Saliva 5% (2%), Blood 7% (2%), Semen 87% (3%)

cg22091609 Vaginal 57% (16%), Saliva 30% (8%), Blood 33% (4%), Semen 2% (1%)

VG_chr22_l_for TM: 66°C, Länge: 21 bp

VG_chr22_l_rev TM: 64°C, Länge: 19 bp

VG_chr22_l_rev_alter TM: 66°C, Länge: 19 bp

VG_chr7_I Cg09765089 (Lin 2016)

TGTAAAATGGAAAACAAATGCCGGGGTTGGTAACAAATGGGATCAAAAGCAGCCATTCCAATCTGGCTCCCCGAGGGAAAGGAGCGGGCGTGGCCCTGTAG
GATTAGGGGCGCTTCTGACCTCCCCAAGACCCAGGTGAGGCCGAGGGTCCCCGGCGCGCCAGCCTGCGCCGTGGCTGTGGCTGCGGTGGCGACTCGGGCCG
GGCCTCGCTCTCGCCGGCTTCAGGTTCCCGCCTCCCTCGCGCAGGCAGCGGGCGCGTGTGGCCCGGGCTGGGCAAGCCGAGGAACAGCGAGCCCCCGGACGC
TGACTGCAGGACGTCCCAGTTTGTGCCCGGGTCTCCGTCCCTCCCCGTACGGGGCTCGTACCCCCGGGCCTGGGTCTGACCCACAGGGCGCTGAGGCCTTTGT
AGCTGAAGTCGGAAGGCCTCGTTGCGAGCGCGGCACAGGTTGCTGGTAGCTTCTGGACTCTGGAGGCTTGGCCTTCCTTCTAAGCCGATGGCGG

Cg09765089 (Vaginal 40%, Sperm 1%, Blood 5%, Saliva 5%)
cg22158650 (Vaginal 33%, Sperm 1%, Blood 1%, Saliva 1%)
cg02456087 (Vaginal keine daten???%, Sperm 1%, Blood 11%, Saliva 1%)

Primer Paar 1 (3 Schnittstellen, Blast Prima)
VG_chr7_I_A_forw (60°C)
TGTAGGATTAGGGGCGCTTC
VG_chr7_I_A_rev (56°C)
AACCTGAAGCCGGCGAG

VG_c7_IA.2_for

CTGTAGGATTAGGGGCGCTTC

VG_c7_IA.2_rev FAM (zweiter umrandeter Primer)
AACCTGAAGCCGGCGAGAGC

Primer Paar 2 (allerdings auch mögliche andere Produkte)
VG_chr7_I_B_forw (60°C)
CTGCAGGACGTCCCAGTTT
VG_chr7_I_B_rev (62°C)
GCCTTCCGACTTCAGCTACA
Produkt: 117bp

VG_c7_IB.2_for (dritter umrandeter Primer)
GACTGCAGGACGTCCCAGTTT

VG_c7_IB.2_rev FAM (vierter umrandeter Primer)

GGCCTTCCGACTTCAGCTACA

VG_chr7_I_C_forw
TGTGGCTGCGGTGGCGACT
VG_chr7_I_C_forw
GTACGGGGAGGGACGGAGA

VG_chr2_II cg14991487 (Park 2014, Forat 2016) not tested
AGAACAGCTCCCCTCGGGCGCTAGGGGCCGCCCCGAGGGCCCGCCTGCCTCGGGCGACACCGGCCTGGCGCCCCCGCGGCCGCTCCGTGTGCCCTGGACTCG
CCGCCCGCGGCTCGGAAGCTGGAGAGTCAGCGACGGGGCCCGACTGCGGGACCGAGGGCTGCAAGAAGAAGCGAACAAATAGTCCCCAGCGCCTCCTCTGG
ATGCGGTCGCGTCTGTGGTCCTGGCAGCCGCTGGGCGGGCCAGGCCAGGTCGGGCCGGGCCGAGCCGGGCACATGGACCTGGGCCTGCGGGCTCTAATTGC
GGCGCTTATGTTGATGATTTTTTTTTTAATCACAGCAGCCCCCAGTTTAGCGGACTGATTTACTCCCGGTATTGGTAAATATGATCACGTGGGCCGCGCGACCAA
TGGTGGAGGCTGCAGCCTGCGAACTAGTCGGTGGCTCGGGCGCCGGCGGGGAGCTGCTCGGCGGCGGACAGTGTAATGTTGGGTGGGAGTGCGGGACGCCT
CAAAATGTCTTCCAGTGGCACCCTCAGCAACTACTACGTGGACTCGCTTATAGGCCATGAGGGCGACGAGGTGTTCGCGGCGCGCTTCGG

cg14991487 (Vaginal 25%, Blood 10%, Semen 3%, Saliva 8%)
cg09968620 (Vaginal 20%, Blood 1%, Semen 1%, Saliva 8%)
cg09578028 (Vaginal 31%, Blood 14%, Semen 1%, Saliva 13%)

VA_chr2_II_Forw
CAGCAGCCCCCAGTTTAGCG

VA_chr2_II_rev
CGCACTCCCACCCAACATTAC

PCR Produkt_ 166bp

Primer Blast prima aber Werte überzeugen nicht so sehr

VG_chr18_I Cg25416153 Lin 2016 not tested
Chr.18
GAAGGACAATGTAGATGGAAGACCCATGAGCAATATACTGAATCAATTAAAGAGACAGGGCATGACATTGTTCAGGCAGCTGTTAATAATAATAATAAAATTA
GTTGCTATTATTAAGCCTGTCTTAAACTGCAGAGAACAAGACAGACACAGGGGATGACCTACAAGCTTCAAGACTCAGAGAATTCAACAACCTTAAGAAATACC
TCCTGGGCAGAATGCTGTATTTGGAAGGTTGGAAGACTCTGAGCTTGAGAACCCTGAAGAAGGCGGAACTGTTGGGTTGCCGGCAGCCGGGAAGGGTTTGCC
TGACACTCTTGGCTGGGCGGGAAGAAATGGGGGGAAGGAGAAGGGCGGCGCACTGGGCTGCTGGAAGCTGGGTTCCTTCCGAAGGGACGTTACGCTACCGT
CCGTAATATCACCCCAAGCCCTAGCATTCTCAGGAAAGTCGCTTATGAAGCTATGACTCAAGTGCCGCAGTGCTTGGGCCCACGCTTCGCAGCGTGGAATCAGC
GGCGGGGCGCGGCGGCACGAGGAAGCTGTGCAAAGCCGAAGGGGCAGCCTCAGACCAGGAACCTTACCCGTCTGGCGGGTCTTATGGCCGAGTCACCGTGG
CGGCGCGGCAGCTCGGGCCCGGTTCTTCCGGGCGGGCCGGGGTGGTCACCTCTGCAAAGCAGGGTTTCTGGGCCGTGATCGAGTCTTGCTCACGGGCATGTC
GGGAAACGCCTGCTGCGCTCCCTGCCTGAAAATCCACTCTTGAAAATGCACTCCCTTAGGTTAAAACGCTGCCTTTCCTAAGGA

VA_chr18_l_forw
AATACCTCCTGGGCAGAATGC

VA_chr18_l_rev
ACGGTGACTCGGCCATAAGA

Cg25416153 Vaginal 36%, Saliva 4%, Blood 5%, Semen 1%
cg18088442 (Vaginal 34%, Saliva 10%, Blood 11%, Semen 7%)
cg21691166 Vaginal 31%, Saliva 9%, Blood 15%, Semen8%

VG_chr17_II cg27215100 not tested
Chromosom 17; CYBC1

TGCAGCCCCAGACACCATCAGCCAGTCTCACCTGGTCTCTGTGGTTGGGCACGTCACTGGATCCCCCAGAGACCCCGTTTCCTGTGTAAGGAAGGCCCTTTTCCG
TTCACAGCGACACCGACTGCGCGGGGGCTGTTTCCCGCTCAGACTCACGAGCTACAGCTGCATCACCACCTGCCCCGTCCTGTCTGGAAACCTTATGTCCTGAG
GCCGCTAGCCCCCAGCAGCCTTTAAGCTTCTAGCATCTGTGGGCACCCCCTCTCCCCTGTGCTGGCCTCCACCAAGAAAGGAACTGAAAGTCCCATCATGGCTTC

CGGCCGTACTGGCTCTCGCACTGGCATGGACCAACGATCTCAGCTCTCTCTCTGCCCTCCTCCACCTGCTGAAAGTTCAGACTGCACAGTAGAGGGTCCCAAAG CGGCCAGAACCGACGAGTCACCAAAGGGGCTAATCTTCCACAGCGGAGTTCCGCACGGGCGCAGCAGCCCTGCCACTTCACCGTACGCTCCCAACACTGGCCC CAGGGACCCCGTTCTGGTAGTTTCTCAGGGCTTTCCCTGTGAAGTCAAATCAAATTTCTGACATTAAGTAGGGGGTTCAG

cg27215100 Vaginal 71% (24%, min bei 12%), Saliva 29% (18%, max bei 97%), Blood 3% (2%), Semen 1% (1%);
cg09419005 Vaginal 59% (10%), Saliva 42% (11%), Blood 11% (8%), Semen 7% (1%)
cg27381584 Vaginal 66% (16%), Saliva 38% (12%), Blood 17% (7%), Semen 6% (1%)
cg08040471 Vaginal 65% (20%), Saliva 32% (14%), Blood 12% (5%), Semen 3% (1%)

Produktlänge: 144 bp, keine Nebenprodukte
VG_chr17_II_A_for TM: 64°C, Länge: 20 bp
CAGTAGAGGGTCCCAAAGCG

VG_chr17_II__A_rev TM: 62°C, Länge: 20 bp
AAACTACCAGAACGGGGTCC

Produktlänge: 328 bp, keine Nebenprodukte
VG_chr17_II_Bfor TM: 62°C, Länge: 20 bp
GGAAGGCCCTTTTCCGTTCA

VG_chr17_II_B_rev TM: 62°C, Länge: 20 bp
TTTGGGACCCTCTACTGTGC

VG_chr17_II_B_for + VG_chr17_II_A_rev 455 bp lang und ein Nebenprudukt über1000 bp
product length = 455

Nebenprodukte!!!!!!!!!!

VG_chr12_I Cg26079753 Lee 2015 not tested
(Chromosom 12)
GCCGGCAGCTCTGGTCTCCTCCGGAGGGCTGCTGTGCCCCCGCGAAGGACGAGATGACCCCTGCTGGCCGGAGCTGTGGAGGTTGCCTCGCCGAGACCCGGA TCTGTCCAGTCGCTCGTCCCTGAGCACCCTAGGCGTTGGGGGGACGCCCTGTGAGTGTGAGAGACCTCCAAGAGCGGCTGGAGTCTGAGGCGCTGGCCGGGC TTCGTGAATTAGACCTTTATCCTAAGCTCCTTCCCTCTGCCGGGCACTCGGTGTCCCCACAGCAGCTGACCGCCCTCCTGCCCGGCTTCTCATCGCTAGAGGCTCT GCCCTGGAGGCTCCTAAGACCTGTGCCGCCCGCTGGAGCCTGGCATAGGAGCCAAGAAACTGGGCCTCGACAAGACCAAACACAGCTGGACCTTTAGCCAATC TAAACCAGCCCGTTGCCAGCAGGTGATTTCTCTTCTTCCAGCCAGAGCCACTTAGGAGGCAAGTCTTCACTTTGTCAGTTTACCTGGC

Cg26079753 Vaginal 27% (21%, min 7%), Saliva 7% (1%, max: 10%), Blood 9% (2%, max: 14%), Semen 4% (1%, max: 7%)
cg19188855 Vaginal 31% (15%, min: 17%), Saliva 20% (2%, max: 26%), Blood 24% (7%, max: 40%), Semen 4% (1%, max: 5%)
cg01524278 Vaginal 34% (15%), Saliva 22% (3%), Blood 28% (7%), Semen 4% (1%)

Produktlänge 206 bp
VG_chr12_I_for TM:60°C (NCBI), TM:64 (Oligoanalyser), GC%=60%, Länge:20 bp
GATCTGTCCAGTCGCTCGTC

VG_chr12_I_rev TM:60°C (NCBI), TM:64 (Oligoanalyser), GC%=60%, Länge: 20 bp
CCTCTAGCGATGAGAAGCCG
keine anderen Produkte

VG_chr17_III Cg01774894 Park 2014 not tested
LOC404266 (HOXB6)
CCTGCGGTCACCGGGCCCAGGACCCCCTCCCCTAGTCGACCCTCGAACACAGACTCCAGCCAGTACCGGGATGCCCTCTATTCTGCCGGCTCCCTTCCCCCGTTT CGCACTCCTCCAGCGCCCCCTCCAGATTCCCTCCTAGTCTCCTAGGCCTGTGCCGTCTGTCTAGACTCTAGATGGGGGAGGGGAGGAGCAGTTTGAACTCCCAC CTGAGCCTGGGGGGAGGGGCTGGTCAGGTGTGTCTCTTCCTAGTTGCATCTTGTCTTTCCCTCCCTTTCCATCCATCTTGTTCCCACCCCCCGTCATCCCCCCAAC CCAATGATAAATCCAGGCCGTTAATCCGTAATGACGTAGATCGATCCATAGTCCACATTAACGGCTCCTCACTTTCGAGTCCGGCTAATGGACATCAGTTGGGAC TTAAGGCCAACAAATAATCCAACCTGAGACCCCGCGCCTGTTTCTCCCTCTCTCGCTCCGCCTGCTCCCTCTCTCTTCCTTCCCCTTATTCCTCCTCCCTCCTCTGAC TACCACCCCTTTCCTGGTGCCTCATCTTGCCCCAGCTCCCCACCCACAGGGAAACACAGTCCCAGACAGACTC

Cg01774894 (Vaginal 38%, Saliva 9%, Blood 7%, Semen 4%) (Chromosom 17)
cg26279336 (Vaginal 40% (16%), Saliva 14% (4%), Blood 9% (2%), Semen 4% (1%))

cg11352083 (Vaginal 28% (12%), Saliva 11% (2%), Blood14% (2%), Semen 8% (1%))

Produktlänge: 312
VG_chr17_III_for TM:60°C (NCBI), TM: 62°C (Oligoanalyser), GC%=55%, Länge:20 bp
TCTATTCTGCCGGCTCCCTT

VG_chr17_III_rev TM:60°C (NCBI), TM: 64°C (Oligoanalyser), GC%=52%, Länge: 21 bp
AGTGAGGAGCCGTTAATGTGG

Keine anderen Produkte

Cg03874199 Forat 2016 not tested

Vaginal 22%, Saliva 11%, Blood 14%, Semen 5%

cg12020396 not tested

Chromosom 19, EPS8L1
GTAAGGGGCACCCTGGCGTGGGATCTGAACCCCCTCCCGATCTCTTCCAAATGTCCCCGCTCTCCCCAGGCTCTCCCCTCCCGCCACTTGCCAGGGCTGACCTCA
CCGCCATCTTAACCGGGTGTCCACCTCTCTCTGCCTGCCTGGTGCTGGCCCCGCGTCCCCATCGCCGCGCCCGTCTGCTCCCCTCAGAGGGCTTGCTGACGCTGC
GGGCCAAGCCGCCCTCGGAGGCCGAGTACACCGACGTGCTGCAGAAGATCAAGTACGCCTTCAGCCTGCTGGTGAGGACGCGCCCGCCCCTGGGCCGGGGCG
CGGGCACGACGAACCTGTCCCGTCCCCGCACCCACGCCAACCACCTCCCTCCCCACGCCCCAGGCCCGGCTGCGCGGCAACATCGCCGACCCCTCCTCTCCGGA
GCTGTTGCACTTCCTTTTCGGGCCTCTGCAGATGGTGAGACCCGCCCCAGGCCCTCGGGCCCCCCTGCAGCGGGAGGAATCGGGTTCGACTTGTAGAAGGTGT
GGCGGCACAGCCTGCCCCTCCTGCTCCCCTGACAGATTGTGAACACGTCGGGGGGGCCGGAGTTCGCGAGCAGTGTGCGGCG

cg12020396 Vaginal 49% (20%, min: 2%), Saliva 3% (2%, max:7%), Blood 3% (2%, max: 8%), Semen 1% (1%);

cg20166714 not tested

Chromosom 12
AAGTCACTTTCCAGAAAGATTATTCCAATAATAAGGAACGCTGGCGGCCAGGTGGGATGGAGAGGGTAAGGCACTGCAGGCCCATGTGAGTCCCTTCCCCGCC
CCCGACTCTAGTTCCAAGAGGTTCCGGAGCTGGTGGCGCGCTTCGACGGCCTGGCCGAGACGCAGGCGGCGCTGAGGCTCAGGGAGCGCGAGCAGCTCGCG
GAGCTGGAGGCGGCGCGAGCGCGGCTGCAGCAGCTGCGGGACGCCTGGCCGGACGAGGTGCTCGCACAGGGCCAGCGGCGGGCACAGCTGCAGGAGCGCC
TGGAGGCTGCCAGGGAGCGTACGCTGCAGTGGGTACGACCCGCCCTAGGTGGGGGCGCTCCGGACCCCAGGCTTCCACAGCCGGGCGGGGAGGAACGCCA
GGGCTGATGAGGACCGATGGGGCAATGCTTACCCCAGAGGGTCCGCTGCACTAAGGAGGGGAATCTCAGGCATCACCGAGCCGTTTCGGGCATGAGGCCCCG
CCCCATCCGCCTGCACAAGGCTCCTGCACCCCACTCCCGAGAGCACCTGACTGCCTGACTGGCAGAGGTGGATGGGAAGAATGCCGGGCGCGGT

cg20166714 Vaginal 42% (21%, min: 0,4%), Saliva 1% (1%, max: 4%), Blood 2% (1%, max: 3%), Semen 1% (0,5%)
cg19897003 Vaginal 46% (21%), Saliva 7% (2%), Blood 7% (1%), Semen 1% (1%)
cg03669590 Vaginal 41% (15%), Saliva 13% (2%), Blood 16% (3%), Semen 5% (1%)
cg27127017 Vaginal 36% (19%, min: 0,5%), Saliva 2% (1%, max: 4%), Blood 2% (1%, max: 3%), Semen 1% (1%)

cg20090283 not tested

Vaginal 53% (min. 8%), Saliva 6% (max. 11%), blood 4% (max. 9%), semen 1% (20%, 2%, 2%, 1%)

cg22052566 not tested

vaginal 55% (min. 8%), saliva 6% (max. 16%), blood 6% (max. 9%), semen 2% (max. 17%) (19%, 4%, 2%, 3%)

Saliva Marker

SA-I cg09652652 Lin et al. 2016

GGCGCTAGGAGGCGGCGGGGGCTCCCTGGGCCCGGGGGCCGGGCCGCCGCCTCTGCTGCTGGGCAGCGCCTCCGACATGAAGGCTGAGCTGTCGCAACTTA
TTAGCGACCTGGGCGAGCTCAGCTTCGGCAACGACGTGCGCACCCTGCAGGCCGACTTGCGGGTGACGCGCCTGCTGTCAGGCGACAGCACGGGCAGCGAGA
GCTCCATCGAGGGCGGGGGCCCTGACGCCACCTCCGCCACCGCCGGGGACTCGTCCCGCCAGGCCGACGGCGCCAGTGCAGACGAGCCCCACTCGGGCTGAG
CTCCTCCGCGCGTCGCCGGCGCTCCACCGTGGCTACCCATCCGTGGTCCCGACAACCTCCCTGTCCCTTGCCCGCCCCCAGGAAGGGGGAAATGGGGCATTTGG

GGCCCAGACCTACACTTGGAGCCCAGGTCCAGCGTTCCCCCGACCGCTTTCCCCTACCTCCCGGCCCCCGCTCCCGCCCCAGCACTTTTGGCTCTGTTGCGCGTG
GGGATGCGGGGAGATTTGAGAGGGGAAAACCCCGCCAGGAGGGAGAGAGAGGCACCCCTCTGGGATGCGGGTGAGGGAAGGTTGGCT

cg09652652 (Blood2%, Saliva 34% (max80%), Vaginal 2%, Semen 1%)

cg16578537 (Blood 3%, Saliva 14%, Vaginal 2%, Semen 2%)

cg00352417 (Blood 11%, Saliva 33%, Vaginal 14%, Semen 2%)

cg10623840 (Blood 8%, Saliva 22%, Vaginal 9%, Semen 5%)

SA-I for: TM: 60°C, Länge: 18 bp

SA-I rev: TM: 58°C, Länge: 20 bp

SA-ll cg09107912 Lin et al. 2016

CCTTCCTAGAGCTTAGGTCACTGTAGCCTCCAGGGGCGCTCCGAGTCCATCTGGAGGGCCGCGAGGCTTCCAGGCTGGAAGGAGGAATAGGGAGAAGGAGG
AATAGGGAGACCTGGGGCAGTGTTACAATTACAGAAAAGGGAGAGGCGAGGTCCGCTGAGTCCTTGGCCTGGGCAACAAGGCACACTGAAAACTGGGTTCCT
TTTCGACCCGCATCGTGCGCGCCCTAGAAATGACAGCCAGACGGAGCAGGGTCTAAGGACGCTGAAAACCCCTGACGTGGGCGCGCCGGGTGCGGGTAGGG
ACGTGGAAGGACTGGGCTAGCCACAGGAACTACAGCGCTGCGGACCGGGTGAGGGGTCCCGGCCCGAGTCCCCACTTGGGGCGCAGAGGTGTTTCTGTAAG
GGGACAAAGGGCACTCCTCGGCGCGATGGGCGACTTCCCCAGCCAGAAAATGAGAGGGTTAGACGAGGTGTTTAACTTCCTCAGCCACTTGGCCCGGCACAG
CGACCGCCCCAAGTTCAGAACCGCTAAGTCAGTCCGGGGCGCTCCGGCCCCTTCGTCCCGCGCCCCGCCCGCCTCCAGCCCGGGCCCCGCCGCC

cg08397344 (Blood30%, Saliva 40%, Vaginal 20%, Sperm1%)

cg09375620 (Blood1%, Saliva 16%, Vaginal 1%, Sperm1%)

cg09107912 (Blood 3%, Saliva 22%, Vaginal 3%, Sperm 1%)

SA-II_For: TM:64°C, Länge: 20 bp

SA-II_Rev: TM: 64°C, Länge: 21 bp

SA_II_cut2_forw: TM: 64°C, Länge: 20 bp

SA_II_cut2_rev: TM: 58°C, Länge: 17 bp

SA_II_cut4_forw: TM: 62°C, Länge: 20 bp

SA-II-for-alter: TM: 64°C, Länge: 18 bp

More primer design on SA-II

CCTTCCTAGAGCTTAGGTCACTGTAGCCTCCAGGGGCGCTCCGAGTCCATCTGGAGGGCCGCGAGGCTTCCAGGCTGGAAGGAGGAA
TAGGGAGAAGGAGGAATAGGGAGACCTGGGGCAGTGTTACAATTACAGAAAAGGGAGAGGCGAGGTCCGCTGAGTCCTTGGCCTGGG
CAACAAGGCACACTGAAAACTGGGTTCCTTTTCGACCCGCATCGTGCGCGCCCTAGAAATGACAGCCAGACGGAGCAGGGTCTAAGG
ACGCTGAAAACCCCTGACGTGGGCGCGCCGGGTGCGGGTAGGGACGTGGAAGGACTGGGCTAGCCACAGGAACTACAGCGCTGCGGA
CCGGGTGAGGGGTCCCGGCCCGAGTCCCCACTTGGGGCGCAGAGGTGTTTCTGTAAGGGGACAAAGGGCACTCCTCGGCGCGATGGG
CGACTTCCCCAGCCAGAAAATGAGAGGGTTAGACGAGGGGAGGAACTTCCTCAGCCACTTGGCCCGGCACAGCGACCGCCCCAAGTT
CAGAACCGCTAAGTCAGTCCGGGGCGCTCCGGCCCCTTCGTCCCGCGCCCCGCCCGCCTCCAGCCCGGGCCCCGCCGCC

SA II cut 4 148 for, 66°C 22nt
GAGGTGTTTCTGTAAGGGGACA
SA II cut 4 148 rev 66°C 22nt
GACTTAGCGGTTCTGAACTTGG
Produkt: 148bp

SA II cut 4 148 for, 66°C 22nt

GAGGTGTTTCTGTAAGGGGACA
SA II cut 4 159 rev
CGCCCCGGACTGACTTAGC
Produkt: 159bp

SA II cut 4 148 for, 66°C 22nt
GAGGTGTTTCTGTAAGGGGACA
SA II cut 4 196 rev , Tm 68 °C 18nt
CTGGAGGCGGGCGGGGCG
Produkt: 196bp

SA-IV cg26107890 (Park 2014)

CCTGGGTCAGGCAGTTTCGGATTGGCTGCAGATCCCCGTCTCCGTGAGGCCGCGTCTGGCGAGGTACAGGTGCTGAGAGGCGCTGGGCTGTCCAGAGGCGGC
GCGCGAGGCCGGGTGCGAGGTCTTGCGTGCGCGCCGCGCCCACTTCCTTTGCTGAGGAGCCTCGCGCAGTCTCCGGGGCCTACCACTTCCCCCGACGCCCTTC
GGGCGTCGTCGTGCGGGTCCCTTCCCAGAAGCGGCCTCTCCTTTCCCTTTCCTCGCAGAGCCGCTCTTCCTGCGCCCTCCGAGCCCCACCCTCCTAGCTCCGTCCG
GAGCCCTTCCTCCGCGCTGGCTATTCTCGGTTGCGCCAGCCTCTCCCATTGTCCATTCGCAGGAAAGGGCTGTCTGGCTGGTCGTCGGTCGCTGGTCCAGGGGT
GGGGGACCCAGAAGAGAGCGCCGCCGGAAGCCCCCGCCGCGGCAGCAGCCCGCAGCCCGCAGCCCGCAGCCCGCAGCCTGCAGCCCCAGCATCATTTTTAGA
AATCTCGCTTTCTAAGTTAGTCAACAGTGCAACCCGACGCCCACAGGGAGGGGTATGACTTCCCGACTCCCTCTTCTAATTTCA

cg26107890 Vaginal 6%, Saliva 33%, Blood 3%, Semen 1%
cg12788878 Vaginal 1%, Saliva 25%, Blood 1%, Semen 1%
cg26125625 Vaginal 1%, Saliva 26%, Blood 1%, Semen 1%

SA IV Forward: TM: 64°C, Länge: 20 bp

SA IV Reverse: TM: 64°C, Länge: 20 bp

SA IV_rev_cut2_3: TM: 64°C, Länge: 19 bp

SA_chr16_I cg10096948 not tested

ZDHHC1 (TPPP3)
CAGGGCAGCCTGGGTGGCTACCTACTATACAGGGACCTCCTCCCCGCCCCCGGTCCACTGGCCAGGGTCGGGCTTCTGGATGGCCCGTGGCCCCTGCCATTAA
GAAAGTAAGCGCGACGTCCGCAAAAGCATAAAAAAGTTTATTGGAGCATCACAGCCGGTGGGGCGGGTATTGCTGAGTCGTGGGGGAGGGAGGCCGATCCC
GCCGGCCGTAGGGGCCCCTAAAGTGCACTCGGTGCGGCAAGGATGGGGTGTTGCATAGAGAGTCAGGCCGGCCGCTCTAACTCGGCCCTCCGGCCTCTCGGC
CCAGCTAAGCCAGACCAGCCTCCCGGCCGCCCGGCGCCCTGGGCTCGCCGCTGCTCGGGCTCACGAAAACGGCGGGCGCACGCGCCTGCCGCGCCAGCGTGG
GCTGTCGGCCCCGGCCCCGCGGGGCGGCCAGAGCGGCGCTGCCCAGGCGCGTCTGCGCCACTGGAATCTCGTCCACGGACTCTGCCGACGCCGAGTGGTAGG
CGCCGGCAGGGCCAGCGGCGTGCACAGGGCTGGCGTCCGCGGAATCCGTCGACGAGCTGGAGCGGCGGCTGGGGGCCCTAGGCCCTGCGCA

cg10096948 Vaginal 21% (8%), Saliva 53% (12%), Blood 19% (4%), Semen 3% (1%); Chromosom 16
cg01755541 Vaginal 30% (7%), Saliva 51% (13%), Blood 37% (6%), Semen 4% (1%)
cg10298187 Vaginal 21% (5%), Saliva 46% (13%), Blood 25% (6%), Semen 4% (1%)

Produktlänge: 178 bp, keine Nebenprodukte

SA_chr16_I_for TM: 62°C, Länge: 19 bp
GCCTGGGTGGCTACCTACT

SA_chr16_I_rev TM: 64°C, Länge: 20 bp
CACGACTCAGCAATACCCGC

SA_chr1_I cg06936779 not tested

ACAGGCCGTGGTTAGGAAGGACGGAGAAGGGGCGTTCGCTCCTTTGGGACTTTTCATGCCTCGTTTTTTTTTCAGATGTGGCTTGGTCTGGGCGCAAGGTCCCA
GCAGCCAGCTTAAGCTTACTCTTCTGTGAAAGGGGAAAGTATCCCCTGTGGAAAGCGGTTAAACTTGTGGAGGGGGTGCGGGACGTGAGTTCTTCCCCATGCC
AGGCGAATGGTGTGGCCTTGAGCTGGTCCAGGAGCCGGCTCGACGTGTCTGAGGGAGGCCCCGGAGGGGGCGGGGAGGTGGCCCACAGAACGCGGGTTCT
GTAAAGAGACGTTGGGAAGATTCGATTCCGAGAAGAGGAAGAACCGGATTGAAAGAGAGCCAGGCCGCTGAGGGGGAGGGGGCTGCTAAGATGGCGTCGG

CCTCCTCCGGGCCGTCGTCTTCGGTCGGTTTTTCATCCTTTGATCCCGCGGTCCCTTCCTGTACCTTGTCCTCAGGTAAGCCCGGCAGGGCGTGGGTAGGGAGCT
GGTGAGGAATATGCGATGGGAGGAAGAGCGAGACCTAAGAGGGTACCTGTAGCTGGGAATGTCCTACGTGTTACCGGTAAGTGGGCGCG

cg06936779 Vaginal 20% (5%), Saliva 52% (10%), Blood 25% (5%), Semen 1% (1%)
cg02491717 Vaginal 8% (5%), Saliva 7% (5%), Blood 8% (3%), Semen 4% (1%)

Produktlänge: 527 bp, keine Nebenprodukte

SA_chr1_I_for TM: 64°C, Länge: 22 bp
TCGCTCCTTTGGGACTTTTCAT

SA_chr1_I_rev TM: 64°C, Länge: 21 bp
ACAGGTACCCTCTTAGGTCTC

SA_chr17_I cg15825321 not tested
GCGGGGGCCACAGCGCCGGGGACTCAGGAGCGGCCGCAGGGGCCGCTGCCTGGCGCCGTCGCCCCCGAGGCCCCCGGACGCCTCAGTCCCCCGGCGGCGCC
GGAGGAGCGGCCGGCCGCGGACACGCGCTCCATTGTGTCGGGCTACTCCACCCTGTCCACCATGGACCGCAGCGTGTGCTCGGGCGCTAGCGGTCGGCGGGC
AGGGGCGGGGGATGAGGCGGACGACGAGCGTAGCGAGCTGAGCCACGTGGAGACGGACACTGAGGGCGCGGCGGGCGCGGGGCCTGGGGGGCGCCTGAC
ACGCCGGCCGTCCTTCAGCTCGCACCACCTCATGCCCTGCGACACTCTGGCGCGCCGCCGCCTGGCCCGGGGCCGCCCAGACGGCGAGGGCGCGGGCCGGGG
CGGTCCCCGCGCCCCGGAGCCGCCCGGCTCGGCGTCGTCCAGCAGCCAGGAGTCGCTGCGGCCCCCGGCGGCGGCGCTGGCCTCCCGGCCCTCGCGCATGGA
GGCGCTGCGTCTAAGGCTCCGCGGCACGGCGGACGACATGCTCGCCGTGCGCCTGCGGCGGCCGCTGTCGCCCGAGACCCGGCGGCGCCGGAGCA

cg15825321 Vaginal 26% (11%), Saliva 52% (10%), Blood 15% (8%), Semen 1% (1%)
cg07118262 Vaginal 35% (7%), Saliva 53% (10%), Blood 33% (6%), Semen 5% (1%)

Produktlänge: 117 bp, mehrere Nebenprodukte

SA_chr17_I_for TM: 60 °C, Länge: 18 bp
GATGAGGCGGACGACGAG

SA_chr17_I_rev TM: 62°C, Länge: 19 bp
GTGGTGCGAGCTGAAGGAC

Nebenprodukte!!!!

Cg16732616 Lin 2016 not tested
Vaginal 1%, saliva 28%, blood 1%, semen 0,2% (2%, 10%, 1%, 0,3%)

Cg20691722 cg02213103 Park 2014 not tested
AATAGGGAAATGTGGGTTTGCTTAATTGTTTAAAATCAAATAGAAATAATTACTGTGCTAAATGAACAAGATGTTTTAAGATTTACA
TCTTCAGGGTTATGGTTATTGATTTTAACTTTACCCTTAAGAAGTTTAGAGATCGCGTTTTTAAAGTTTGCTTATTGACGGAAATCG
GCTTAATTTTTCATAAAAGAATGGCTTTTTAAAATATTGAATACTATGGCTATGCAAGGTTTATGATACCTTTATGTTTATTCTTCC
CACCTCCCAAAATTAGCGAATTCGAGATAACATTTCAAGGGGAATTGCTATTTAGTGCGATGAGAGATGACTTCTCCATCTGGTTCC
AGAATTAGATCTCGGCTTAGGTTTTTTAGTAAGTAGGTTCAGATGCAATGGAAGGAGAGAAGAATCAAGCCTTTTGATAAAATCTCT
ACCCCGAATTCTATGGAAAAGACAGGCAAGCGGGATTCCAGCCACAGCACCCCCACCTTTTGCTCTACCAAAAGGCTTGTCCCCATT
ACAGAAATAATATAGTCGGTGCTAGTTTTGTGAACCCGCGCCAACCGATAAGGTAAGAAACGGTGCGCTGACAGCCGAGGTCCGAAG
GCTGCTCAGTGGTTCCCGCAGCCCGTGGGGTAGGGAGGCCAAAGCCAGCAAAATGTTGGAGGCGACGCCAGAAAGAGAGTATATGCC
TGCTATTCGGAAGTGATGTCGAACGTGCAATAGCAGAGTCCTGGAGATTTCCACCCCGTTCCGCACCCAGGGATCCCAGCCTGTGGG
GTAGCAGGTTGTTGGGTG

Cg20691722 Vaginal 6%, saliva 29%, blood 7%, semen 2% (2%, 15%, 3%, 1%)
cg02213103 Vaginal 16%, saliva 40%, blood 21%, semen 4%
cg16276063 Vaginal 16%, saliva 42%, blood 12%, semen 1%

Cg15227982 Forat 2016   not tested
Vagina 22%, Saliva 32%, Blood 32%, Semen 82%

Cg21597595 Forat 2016 not tested
Vagina 5%, Saliva 19% (min 13) , Blood 5% (max 10), Semen 1%

Blood Marker

BL-l   Cg04011671   Lin et al. 2016)

CGGAACTGGCAGTCGTGGGGAGGCCCCCAGGACGCTGTGGCCACGGGGGCGGCTGGAGGAGAGGAGGCAGGGGGCTGGGGAGTGAGCCAGTAGGGAG
CCGGGCAGCACACTGGCCACCTGAGAGGTGTGATGGGGGAGACAAGAGGCGGTGATTCCTGGGGTGGGAGTTACCCCCTCCCCAATTCTCAAACCCCCGAC
AGGAGTGAGCTGCGCTGCCCACTCCCCGCAGCAGGTGGCGCCGTCTTGCTGGGATTGGACTTGCGGGCCTGGCTGGGAAGCGCTCTGTGGAGAGGGACGCG
ACTGGAGCGGCTGGGGGGCAGCCGTCCTGCGCCTGGGACCCTCACGGGGCCTCCCTGCTCCAGTCACTCCCGCCCGCCTGCCAGCCTGTCCCCAGCTCTCCTGC
GTCTCCACTCTGTCCCTGTTGCCCTTCCTGTCTTCCTCCAGCCCTCCAGTCCTGCTTTCGGCTGGGGTCCCCGGACAGGGTCTGGTCAGCTGGTTCACTCCGAAGC
ATCCCTACCGGATAAGGGAACTCTCAGCCCAGCCCAGCGGGCGTTTGGCCATGGCGTGGCACCGTGGGGGGCCTCTCTAAGCAGCCCCG

Cg04011671 (Blood 25% (max 39), Sperm1%, vaginal 6%, Saliva 3%)
cg03717364 (Blood 34% , Sperm1%, vaginal 10%, Saliva 3%)

BL-I for: TM: 60°C, Länge: 18 bp FAM

BL-I rev: TM: 60°C, Länge: 18 bp

BL-I_cut1+2_rev: TM: 60°C, Länge: 18 bp

BL-I_cut3_forw: TM: 68°C, Länge: 22 bp (erster umrandete Primer) FAM

BL-I_cut3_rev: TM: 56°C. Länge: 16 bp

BL-I_cut4_forw: TM: 60°C, Länge: 18 bp (zweiter umrandeter Primer)

BL-I_cut4_rev: TM: 62°C, Länge: 20 bp

BL-I_cut3_alter = BL-I_cut3_forw + BL-I rev

BL-I_cut3+4 = BL-I_cut3_forw + BL-I_cut4_rev

BL_chr1_l   cg02431672

GCAGTCCCCTCACCGGGCGCTCCCAAACAGCCCGCGGGGCGCTGGGCGCGGGGCCAGGAAATCCGGCGCCGGCCCTTGAAGCGCACCCGGGCCGGGAGGGG
GCGGCGGGGGCGGCGGGGGCGGGCTGGGCGCTGGCACCACGACCGCCCGCTGAGCAACGTGACTGCGCGGGCTCGGGTCCCCACCAGGACAAGGCGGCCT
CTGTGCCGACCCAGCTGATTCCTCTCGGGCTGCAAATCCCTTCCGGCTGACAGCCCTGCCGGCTGGGGCCAGGCAAGGGAACGGGAAGCGGGGTTGGGGGCG
AGGTGGGCGCTGAGGATTGGAGCCCATTGGCGGGAGGCCCCCAGGAAGGCCGTCTGCTCCGTACATCCAGGGTAGCAAAGGCTACCGTTCCCGGACAGAGA
AGCCGAGGCTAGGAGAGGTCCAATTACTAGCCCAAGTCGCACAACTTGTTTCTTGGTTAAAGCCAGAGTTCTAAATGACGCCCCATGATAAACTCTGTATTTGTT
GTTGAGAGTTAGGTGGCGTTATTAGTCTCAGTTTATAAATGAAGGAATGTGAGGCTCAGAGTTGAAGCCGCCAGCCCAACGTCCTAAGACAAGAAGGGAGTG
AGGCTGGGACTGGAGCCCAGGACTGCCTGCTCCAAAACATGGGGGCCAGGCAGTCCCAGCAGACCCCTGACCCCAGGTAATCACTGACAA

cg02431672 Vaginal 8% (3%, max: 14%), Saliva 8% (2%, max: 15%), Blood 26% (4%, min: 18%), Semen 3% (1%, max: 4%)
cg14780649 Vaginal 17% (2), Saliva 19% (3), Blood 21% (2), Semen 17% (2)
cg18153322 Vaginal 6% (1), Saliva 8% (1), Blood 9% (1), Semen 6% (1)

BL_chr1_l_for TM: 62°C, Länge: 20 bp

BL_chr1_l_rev TM: 64°C, Länge: 21 bp

BL_chr1_lll   cg19637387

AGGTAGCTGGGGTGGGATTGTCAGCATTTTGCAGATGAAGGTCACAGGGGTAGTGTTAGGAGTGAAGGAGAGTCAGGACCAGCGTCTCCTGATGTCCAGCAG
GGAGGGCTCCTAAAGATCCTCTTTACTAATGTGGAAGTTGAGACCCAGTGGGGGTAATGGCCCGTCCTGGGCACTCAGTGCATGAATGCTGGAAGCCAAGAGT
CCCCTGCCCAGCTCGGGGCTGGCCGGGGCCTCCACATCTGCGTTCCTGCAGGGCGCTGAGGGAATGCCCCTGGCTGGGCGGCCCCGCCCCTGCTGGGATCCAG
GCCAACGCTCCACAAATGAGAGAGCTTGAGCGCGCGGCGGGCGCCGCTGGGAGAGGCCACACGGTGGCGCTAGCGCCCAGCCTGGCCAGCACCATGGGCAG
GGCCGGGGCGCCTGGCTCCTTCCAGGCAGTTCTCCCACGCGCCACCTCGCGTCCCACAGACACGAACTCCTGCCGCTCCCCCCGGCATCTGTCTCGTCGCGGGA
AGTGGAAACTCCATCCTTCAGTCTCTTGGGCCGTAATCCTTGGAGTCCTCCATGGCTCCTTTTCGTTCTTCCCCCTCTCTTCTGCCCC

cg19637387 Vaginal 8% (2%), Saliva 8% (2%), Blood 22% (4%), Semen 6% (1%)
cg15482882 Vaginal 8% (3%), Saliva 7% (2%), Blood 23% (4%), Semen 5% (1%)
cg09160123 Vaginal 26% (6%), Saliva 34% (5%), Blood 50% (7%), Semen 7% (1%)
cg05388840 Vaginal keine Angaben, Saliva 8% (2%), Blood 27% (3%), Semen 6% (1%)
cg12034678 Vaginal keine Angaben, Saliva 1% (1%), Blood 7% (3%), Semen 1% (1%)
cg11177980 Vaginal 20% (8%), Saliva 8% (3%), Blood 34% (3%), Semen 4% (1%)

BL_ch1_lll_for TM: 64°C, Länge: 21 bp

BL_ch1_lll_rev TM: 64°C, Länge: 21 bp

Bl_1_lll_new_r: GGCATTCCCTCAGCGCCCT

BL_chr2_I   cg02345886

CGTTAGAGGACCTGTTGACTTAAGGACCTGCATATCGCGGCTATGGTTTGCCTGTGGCATATGACTCAAAGGTTCAAAAAAACATTGTTTCAGGATCCTGCCAC
GTCTAAGCTTGGGTGTGCCAGGCCCAGATTCCTTTCCCTGCCTAATTAGGCTTTTCTGAAGCCTAAGAATTGAGGGTGAAACTGGCGCTTACCTTAGACTTTTAT
TTCCTTCTCTTTCCAACTCATCAAAGAAACTATTTCTGTTTTAAGAACCCCATCGAGTTACCAATATTTCCGATTCTTGAAAACGTTAACATAACTTGCTATAGAAG
TTACACTACATAATGAAGACATTTACAGTCAAATTCTATAGCTAAAAAATATTAAAGTGACAGCATTTTAATTAGAAAATCTCTG

cg02345886 blood 50%, saliva16%, sperm3%, cervix 28%)

Blood chr2 I forward: TM: 66°C, Länge: 23 bp

Blood chr2 I reverse: TM: 64°C, Länge: 24 bp

BL_chr2_ll   Cg06645778

GGGGTCCCTGGACTCTCTCGAGCAGGAGTCAAAGCCTTACCAAATCAAAGTGGGCGCAGTCACTGTCCTCAGAGAGCCTCCATTGAGCCCTGTTTTTAACAAGT
CACTTGCCCAGTCTGGGGTAGTCAGCTTCTCACTGAAAATCTCCTCATACCCTTTGTGGAGTACTTCCCAGGGGATACACACCTCTTTTATTCAAGCCAGAAATTC
CTTTATGGCATTTCCACACTAGGACGATTATGTAACTTCTCAGATAAAGCTGTATCAACATATCCTCTATTAGGTGACATCCTTGGCTGAAACATTCCATTGCATG
AATCACCGGGGCGTTCTATGCTAATAAGCAGGGAGCATGTGTGACCTACTGCAGTCCCAGAGGGCCCCTCAGAGGCTCCTTTTTTTTTTTTTTTTTTTTTTTTTAA
ATTGAGACGGAGTCTCCCTCTGTGGCCCAGGCTGGAGTGCAGTGGCGCGATCTCAGCTCACTGCAACCTTCGCCGCCCGGGGAGGCTTCTTTCTTCTAGTCACT
GCAATTGGCTGTGTGGCCTAGAACATCAATGTGGCTTTTGTGTCTTAGTTTTCCCTTTATGGACCAGAGCTAATG

Cg06645778 **(Blood 50%, saliva9%, sperm3%, cervix 14%)**

Blood chr2 II forward: TM: 66°C, Länge: 19 bp

Blood chr2 II reverse: TM: 66°C, Länge: 24 bp

Neue mögliche Primer
Bl_2_II_for_neu
ATCACCGGGGCGTTCTATG
Bl_2_II_rev_neu
ACATTGATGTTCTAGGCCACAC
Produkt 241bp

BL_chr6_l Cg08792630 (Park et al. 2014)

CGTCACTAGCTGAATGAGAACCTTCGGGTCCAAGTTTCAGCTTGTGGGTGTTAACACCTACAGGCACATCGATCCGATTAGAAAAAGCAGTGGTTGCAAACCTT
TTCCTGGACGGCTTCCTTTCCTTGCCTATATTGATACCTTTTCTTCTCGGAGATGTCGCTCCAGTAAACCTGCTTCTGACTAGCTGCTTCTGAAATGTTCTGGGGCC
TCGAACCGGCCGGTCTGGCCACCTCAATCCAGACTGGCTGCACCCGCTGCTCCCGCCGAGGCCTGGATTCAGGCACTGTTTGAGGGAAGGGCTCTGGTGTGGG
GGACGCGGGGCCTGCGCTTCATTCACTCTGGCGTTTGGGAGGAGGTTTGGGGGCTGGGCCGGGCCGTAATGGCTTCTACCTGTAAATGAACCCGAAGAGAGA
CTCTCTACCGTTCCTTATCATCCTCTTGGGGCACGGGACACGTGGTAGGGAGAGAGGTGTGGAAAGGCCTATGATGTAATGACTTCCTGCAGTTGGGTATCCAA
AGAGATTAACTCCTCTGCCCCTGCTGGGCTGCTGATATCCAGCTGCTTTCCTCTTCCTAGGCACCTCCCTGGCCCCTTCCCCCAAGACAGTGTGTGTCAGTACCAG
GCCAAAGAGTCCTGTGCGATTGGAGATATACATCTCTTATCAGCTGCCCATATTTGCAATTTAGCTTAGATTTTT

Cg08792630 Vaginal 13% (6, max: 26%), Saliva 10% (3, max: 19%), Blood 37% (6, min: 26%), Semen 5% (1, max: 7%)

BL_chr6_l_forA TM: 62°C, Länge: 20 bp

BL_chr6_l_revA TM: 64°C, Länge: 20 bp

BL_chr6_l_forB TM: 62°C Länge:20 bp

BL_chr6_l_revB TM: 64°C, Länge: 21 bp

BL_chr11_l cg04434593

GGGCAGTGGTGGGTGAATTGGAAAGGGTGTTGTTATCAAGAGGGGCGTGGGGGGGGAGGAGAGTGCAGGAGCCAGGTGTGCACAAGAGGTGGGACGTGG
GGAGGAATGTGAAGTGTCAGTGAGCCAGTGGCTGGTGTGCAGGGGTGGCGTGCAGGGGTGGTGTGCAGAAGTGTGACGGACAAGAGTGTGGGTCTGTGAA
TGAAGGGGACAGGGAGCCATTGCACCCATCAGCAGTGTCACTTCTGGGGCTGCCGGGTGACAATTGCCATCTCTGCTTCCTCCCCGGTGGAGCGTGTGGCTCC
AGGGCAGCGAGGGTGTCTGAGCTGGCCAGGAGCGCGTCTGGTCTGTTAGGGGAATATGTACTCGCATCCTGTCCGGTGTGAGGGGCAACGTGTGCTCCCGAG
CTGTGGCGGAGGTCAGTCGGGGTCAGTGAAAGCACAGAGGTGTCCCTGGGTTAGGGTCAGAGAAGGAGGACCCAGTAGAGCAGCGTGCCCAGTGCCCCCCA
CCCCTAGCCTCTCTCCAAGGCTTAGCCTCCACCCGCCCGTCTCCCCTCCTCTCCTGGTAGCCCCTCACACCCCGGGGGGAGGGCCAGGCTGGGAGCTGGGGGAA
CAGGGGTCCTCTGGGATTGGGAGGGTCTGTGGGGAGGGCTCCAGAAGCCCAGCACGGAGTTGTGGAAAGGAAGGCGGGTGGGGAGAGCAAGC

cg04434593 Vaginal 7% (3%, max: 14%), Saliva 8% (2%, max: 12%), Blood 22% (4%, min: 15%), Semen 2% (1%, max: 4%)
cg14588642 Vaginal 11% (3), Saliva 10% (4), Blood 17% (3), Semen 36% (5)

BL_chr11_l_for TM: 64°C, Länge: 19 bp

BL_chr11_l_rev TM: 64°C, Länge: 21 bp

BL_chr12_l cg22249612

GTGCCCCGACACCCACGGCAATCAGTCCCACTGCACAGGCCTAAGAGAAAATCAGGTGAGGATTAGGCCATATCCACAACACAGTGGCCCTCCATGGTGGAGG
GCAGGGGGTACACCTAGGACACAGACTTGACCCCATCCGGAAGAGAGATTCTCACACCTCTAGGGAATTTCTTTGGCTGTGGGGTAGGGGGATGACCCATCCT
TGCCTGCCCCTACCTCCCCTGATCCCCTCCCGGCTCTTTAATCAGGGACATGACCAAATTCAAAAACCTCCCAGAAAGCCTTGACCTCAAGACACGTATTTCTGAC
CTAGAATAATGCTTTCTACAGGCCTCCAGGGCTCCCAGCTCAATTCTCTCCCAGAACTGCCCCCTCACTATCCTCCAGTGCACATCCCACGCGCGCATCAGCTGTG
ACTAAGGTTCCTCTGGCCAGTTAGGCCAGGAGAAGGGAAGAGGAAAAGGGAGCAAGAGTGATGTGTCCCTCCCTAGACAAACAGTAGCCTCTCAAGCCAGGA
GCCCAGGGCAGGAGCAAATTGAAGTGGGCAGAGCCCTTCCTCACCCCCAAGCGCTGGGAGGAGGGAAAGGGGGAGAGGGGCTGGACAGGGATGAGAGGG
TTGGGGAGGGAGAGAGGGGAGAAGGGAGAGCGAAGGGAAGCCCCATGGCGATAGCATCAGGCGGTTGGCTGGTTGCCCCACT

cg22249612 Vaginal 7% (4%, max: 15%), Saliva 7% (3%, max: 16%), Blood 35% (5%, min: 28%), Semen 1% (0,5%, max: 2%)

BL_chr12_l_for TM: 64°C, Länge: 20 bp

BL_chr12_l_rev TM: 64°C, Länge: 20 bp

BL_chr14_l cg23051349

TAGGAGCTCCCCCTTTGTCTTTATAAATGAGATAAAAGTAACCAGACCTCCCTGGAAAAACCGAGGAGCACTTGCTTCTCTGGTGACTGCGGGGCCTAACTGCA
GGGTTCACAAAGGGCTGCTGGTGCTGGGTGATAACATCGGAAAGGAACAGCAGTTCACAAGATGGGGCTGGGGGACTGGCCCAGGAACCCCTCCAGGTTAA
ATGATGCCTCCGGGCCCCGCACTTGGGACGTCTGCAGGCACTGGGAGGGTTTTTCCCAAGCCGCTGGCAGCAGCGAGCGGAGCCTCGCCCATTGAATCAGCAT
GCCCTCCCGGCTGGCTGCTGAGTCAGGCCTGCGGAGCCGGCGAAGCCGCAGGACTTGACCCAGGCGCGCTCCGCTGCCCTTCAGGCCGGGCTGGAGGTCGAG
GCCCTGCAGCCAGGGAGATGCGCCGGTGGCTGCGTGGGGACAGAGGGGGGTTGTCTCCCACCCTCACAGTCTACACCCCGGCCCTTCCCCTTTGCCCGTCTGA
GGCGCAGTTTGTGTCGAGGGGGAGCGCCTGGAAAGTTTCCTCGTACAGATACGAGGGTCCAAAAGCCCCGAGGAGAGGGAATAATGAGAAGGGGAGGGCG
CAGGGTTAGAGGTCAGGGTCTCGACCAAAGGCTGTCGGTTTTCCTGGAGGAGGGAGAAAGGCCTGGCCGGGATCATCTGATTCCGGGGCCTCCCGAAGGCCT
CCTAGCTGGGTTTTGGGGGGACCGAAACGAGCGCATGAAGCTCGCGGGCCTGGGAGTGGGGTGGGGCGGGAGCCCAGTGGTGGCGG

cg23051349 Vaginal 8% (4%, max: 17%), Saliva 4% (2%, max: 10%), Blood 27% (4%, min: 20%), Semen 2% (1%, max: 3%)
cg23090046 Vaginal 27% (7), Saliva 28% (8), Blood 54% (7), Semen 7% (1)
cg05321721 Vaginal 11% (3), Saliva 12% (2), Blood 16% (2), Semen 9% (2)

BL_chr14_l_for TM: 64°C, Länge: 18 bp

BL_chr14_l_rev TM: 64°C, Länge: 20 bp (zweiter umrandete Primer)

BL14_l_rev_alt TM: 62°C, Länge: 18 bp (erster umrandete Primer)

BL_chr16_l cg06329093

TATAAAACTTAGCCGGGCGTGGCGGCGGGCGCCTGTAATCCCAGCTACTCCAGAGGCTGAGGCAGCAGAATGGCTTGAACCCGGGAGGCGGAGGTTGCAATG
ACCCGAGTCGTGCCACTGCGCTCCATCCAGCCTGGGTGACAAGAGCGAAACTCCGTCTCAAAGAAAAAACTAAAACTCTTCCCATGGTTTCCTTTTAAAACAGA
CCCTCCATGACCAACCCCTCCATTTCAATACCAGAGAGCCTAACCCGACCTGAAATCACTTTAGTCATGTATGACATCCGCTTCCCAGCCCCCTTCCTTAATGCTCT
GGGTTCTGGAGACAGACCACGTCTGTTGGGAGTCCTCTGTGCCCACGCCTGGCACAGAGCCTGGCAAAGAGTAAGCGCTCAAGGAAGATCTGTGAACTTGATG
CATGCTAAAAGGAAGCCCCGCGCTCTGCCGGGGCCCTGGAACTCAGGCCACCGGCTCCAGGCGGAAGCGAGCCCAAAGCCGGCCAGGCGGCAGCATCCGCTA
CCCCGTGGCTCAAGCCAGGCTCCGAGGTGAGAAACCGGGCGGGGACTCCTCACCTGCGCGCCGCGGAGGCCGTGCCAGATGGG

cg06329093 Vaginal 8% (2%, max: 13%), Saliva 9% (2%, max: 13%), Blood 22% (4%, min: 15%), Semen 5% (1%, max: 7%)
cg11657615 Vaginal 12% (3%), Saliva 14% (3%), Blood 31% (6%), Semen 7% (1%)
cg00420361 Vaginal keine Daten, Saliva 7% (2%), Blood 9% (3%), Semen 5% (1%)

BL_chr16_l_for TM: 62°C, Länge: 20 bp

BL_chr16_l_rev TM: 64°C, Länge: 23 bp

BL_chr17_l cg05164926

CCAGCGTCTGCCAGTCCAGCCAGTCCGCCCAGTCTCTCGCGTCCGAGACTCGCCTCCAGCCTCCCACCTCCGCCCGGGCCGCGCGAGCCTCGCGGGGGCGGGG
GCGGGGCGCCAAGGGGCGGGGCTGTCTCTTAAAGGGCCCCGGGCCGCTGCCCTTAGGCCACTTCCTGGGGGCGGAGAGGACCTCAGCGGCTGCGGCGACAC
CCAGGGAAGGCGGCGCGGCCGGGTCCCGAAACTCCTGGCTGTTTCCATCAGAGCCCTCGGACACTCCCAGCCCGGGCTGAGCACGCATCGTCGCTCCCCGGCG
GATACAAGGGGGCTCCGCCATCCGCTCCCGTCAGTTCGGCCTCCATCTCCTGGGACCCGCGCCGGCAGCCAGGCCAGGCCTCTGAGTGGCCCCAGAGCCCTGG
CTGGACTCGTCCACGGCGGCAGCGATCTGCCCGGGGTCTCGGAGGCCATCCCTTCAGAGTCGGCCCTGTGCTCGCCACCGTCACCTGCTGGTTGGATTCCGGA
AACCCACTGTCTGAAGACCACAGAGGGGTGTCGCTGACCACCCCAAATCGGATACGTCCAGACCTCAAGCTCCCTTCCCCTCTCTGGCTGCCCTCTGCTCTTTTC
ATCTCTTCTCTCAACCTTTTGGGGATTTCTGTG

cg05164926 Vaginal 7% (5%, max: 17%), Saliva 6% (4%, max: 18%), Blood 29% (6%, min: 20%), Semen 1% (0,4%, max: 2%)
cg02550691 Vaginal 9% (3%, max: 14%), Saliva 9% (3%, max:18%), Blood 21% (4%, min: 14%), Semen 5% (1%, max: 6%)
cg03982355 Vaginal 7% (2), Saliva 10% (2), Blood 11% (2), Semen 7% (1)
cg03407547 Vaginal 1% (0), Saliva 3% (1), Blood 3% (1), Semen 2% (1)

BL_chr17_l_for TM: 66°C, Länge: 21 bp

BL_chr17_l_rev TM: 64°C, Länge: 21 bp

BL_chr22_l cg19343611

AGAAAGCCGGAGGGGCTGAGAGAGACCCTTGGAGACCGGGGGGGGGCGGGGGGAGCCCGTCCCCGCCCCCCTGCCCCAGGCCTGCTCCACGGTCCTGGAGA
CCCCCTGACTTCCGGGGGCTGCGCTCTCATCGCACGGCGGCCGGCGGCGTGGTCAGCACCCGTCAGGCTGATTTACTGGCGCTCGCCGCCCGCCGCGCGGCGG
CAATCAGCCGCCGCCGCCAGATCTATTATTGATGAACTTTCCGAGGCCCGCTAATTAACTGCGCGCACCCACGGCGGGCGCCGTGGCGGTGGCGGCTGAAATA
CGGCCTGCATGTCCCAATGTCACGGGCGCCGCGCCCGCGGGATGGTCCCGAGAAGAGGGACGGGATCTCAGCCGCCGGCTCAGTCCCAGCACCGCCTCCCCC
ACCACCCGACCCCGGGGTGGGAGCCCAGCGGGCTCTAGGAGGGCCCCCTAGCCACCCCATGCGTGGCCTTGAGCAAGTGGCTTAATCTCTCTGGGCCTCAGGC
ACAGATCGTCCCTGCTCCCTGCAAAGCCCTGCGCACTCCCTGCAAACCTTGGCAGGCAGCGGGTGGCAGAAACCTCCAGTGTCTGGCCCATCCACGGAGCTGG
ATTCCAGTCTCGATTCTGCCACTTCCTGGCTGTGTAGGAATTTGACCTCTAGGAGCTCCAGTTTCCTCATCTAGAAAACTGGAATA

cg19343611 Vaginal 8% (4%, max: 16%), Saliva 8% (4%, max: 18%), Blood 23% (5%, min: 16%), Semen 1% (0,4%, max: 1%)
cg14757543 Vaginal 21% (6), Saliva 23% (6), Blood 36% (5), Semen 11% (1)
cg18009127 Vaginal 14% (2), Saliva 17% (2), Blood 20% (2), Semen 15% (2)
cg27004669 Vaginal 25% (6), Saliva 37% (5), Blood 47% (8), Semen 6% (1)

BL_chr22_l_for TM: 66°C, Länge: 20 bp

BL_chr22_l_rev TM: 66°C, Länge: 20 bp

BL_II Cg18454288 cg26163537 (Lin 2016)

CCCAGGACACCAACCCTCAGGCGGGCGTTATTAGCCCATTGTAAAGATAAGGACACCAGGCTTGGCAGCATTAAGAAAAAATGAAGGGCCTCTCACTCCACCC
GCACTTCGATACTCATACGCAGATGGGGCAAACTGAGGCTCAAAGAGGTGAGGCCCCAGACCTGGCTGGCTGTGAGCAAACCCAGGTTTCCTTTTCCCAGGCC
GGGTGCCTTCAGCCCACGACAGAGTACGGGGCAGCCCCAGCTCTTCCGTCCCGTCCTGCTGCGCCCAGGATAAAGGATCTGGACTGCGCAGTTAATACTTTGTG
CGTTTCAAACAGTTCGCTTATAAATTGTCTTTCGGGACAGGTCCAATTTGTCCTTTCACTGGCGAAGCCTCCTGAGCCGCGGCGGTCGTGGTCCCGCGCCCGAGT
CCCGAGGACAGCAGGAGGGGGAACCCGCTCAGCA
GCCCGGGGCAGGAACCTCCGGGAGCTTCTGGCCCCCCGGCCGTCCGGCCCG

Cg18454288 Blood 15% (max 25% große Abweichung), Sperm 2%, vaginal 4%, Saliva 4%
cg22281380 (alles nicht methyliert) (Vaginal 3%, Saliva 4%, Blood 4%, Semen 1%)
cg15774495 Blood 10%, Sperm 1%, vaginal 1%, Saliva 1%)
cg26163537 (Blood 23% (große Abweichung, Sperm 1%, vaginal 10%, Saliva 1%)
cg21517389 (Blood 18%, Sperm 1%, vaginal 1%, Saliva 1%)

Nur zwei Schnittstellen, aber trotzdem nicht spezfisch, evtl weil nur wenig methyliert

BL-II rev: GACCTGGCTGGCTGTGAG
BL-ll for: 6FAM-TCGCCAGTGAAAGGACAAAT

cg00340958 no HhaI cutting site

chromosome 20

atattatgccttcctagaggaacacttttcgtgatgacactttgtaaacaaggcaatctgatgctgcctgttctatcttcccagatctagttgatagttcaaaacatcaacattaaccacaagtaaaatatgcttaaa
aaataatcaggagacgatctcctgctctatctggaaatttctctattcagctagttctgcgttgacaggtcaacagagaatggcactagttattgctacaatcctaaaaggcatttccctacagctaaaccatcaac
cctttcctaaaatccatataatccaaaatttttctagcagaatttactctccctgcccctaccccagcagcatcttctgatcatttttcaccataaactgaggtttcctcatagtgatgccaagtcaggcaaacacatt
ccttaagcgttccacctaactcccttcttcctggaagttacatgtaatagctgcctttatttacttatttttaaaactcttcaagcaccaggtacagtggtaagtgcttttaaagaacatgtatctacttccatccttaac
actgtatgagattgggactcacattcccattttacagaggagaaatgcaaaggcttagtaacctgcccaaggtcatacagggcagagattggaatccaagtctgacctctgtgtcctccgctcttaaccttcatgc
tgtctttcct

cg00340958 (Blood 50%, saliva10%, sperm12%, cervix 15%) on chromosome 20

cg06379435 Lee etal 2015 / Park etal. 2014 no HhaI cutting site

CGGCATCGCGGTTACTGTTCAAAGTTCCCTTTTAGCAGAAGAGAATAAAGGCTCAGAGAGGGGCAGGTCTAGCGCAGGGTCACACAGCACAGTGGGGTGGAG
CCACCTTCTGTGCTGGGCATGACTCCGGGCACAGCCGAGCCGAGGTGGGGGCCAACAACCCCTACCATGCCAGGGTTCCAGGAACTGCCAGCAGGGGCCCAG
GCCATGCCACTGTTGCAAAATCTGAGGGCGGCATACCGGCAAGCCTGAGCAGTTGCCACGGCCTCAGCCCCACGGGCACCGCCAGGAAAGAAAAATGCAACT
TACTCCTGGGCACCGGGACAACCGGTGGAACCTCAGGCGTGGGACGGCTGCCGGAAAGCAGGGCCCAATCAGAGACAGCTGCCTTGCTGTGTGGCCTATCAG
AAGATGGCTGCATATGTCCTGGCCAATGAGAATGCCCCAGTGAGGAAGGCCACGCCCCCCCAAGTGAAGCCCATTGGCCCCATGAAAGGGCTTGAGGTGGGT
CCCTTTGGTCCTAAAATTTCTTCTTCACTCTGTTGGTTGCAGACGCAGGTCAGCTGAGGCTTCCTGATGTGTGGGCACTGAGGGTCGATCTAAAGAAAAATGCA
ACTTACTCCTGGGCACCGGGACAACCGGTGGAACCTCAGGCGTGGGA[CG]GCTGCCGGAAAGCAGGGCCCAATCAGAGACAGCTGCCTTGCTGTGTGGCCTA
TC

cg06379435 (Blood 47%, Vaginal 20%, Saliva 4%, Semen 1% nach LINs Chip nicht Blood spezf.) nach Lees Exp. (Blood 40%, Vaginal 5%, Saliva 4%, Semen 8%

HhaI Schnittstelle relativ weit entfernt

cg01607849 Lin etal 2016 not HhaI cutting site

CCTCCCTGCCAAAGTCAAGTCTCCACTTTTCACTGGTTCTAGAGCTCTAGGAAAATTGGGGTTGGGTGGGGAGGTGGAGTAGAGTGACTAAATGCCGACACAA
AGCCAAGGAAAGATGGAGTGAAGAACCCTTCCCTCTCTTTATTCACACAGGAGTGGAGGATTTCCCAAATGTCCCTAACTGGCTAGCTGGCTTCAGGCTGGGAC
TCAGTCCCTGCAGTTCCTGCCAGGCCTTGCCAGCCGGGGCGAGGGTTGGGATGATCCTGGCGGCCTATGCCTGTGTGGGCTGCCCCTCCCGCTGTGAACCCTGC
ATTTGTCCCGCAAGTTTTCACTCAGGTAGACTCCCTGGGTACAAGGGTGCCTGCTCAGCAGTCGGGCATGAGCTGCTCCGATGGGCGAAGGAGGTTGTCTATCC
CACAGTTGGAGAGGGGCCCTCTCTGCCCCAGTGGGCGATCTGGGCTACGGCCAAGTTGCCACCAGCTAGTTCCGCTTGAAAACCAC

cg01607849 vaginal 4%, saliva 3%, blood 14%, semen 2% (2%, 1%, 2%, 0,4%)

Cg03363565 (Forat 2016) no HhaI cutting site

CGCCCACACTAACACCCCTATTTAGAAAGGTTGACACATCACTATCACTGCTTCTGAAAATTTTATCCGGCCGAAGGCATCAGCCCAAATTTGAATTCTTGCCAAA
GCTGCTGGTGATTTCGTTCTTTGAATGCAGTATCTTCCTCATGAACACGTACAGCTCTTACAAGAAAATGAGAATACGGTTTGGGGAGGAGGATCCCTCACATTC
CTTTTCCTAATGCCAGCCTCGCGAAGTCTGGTCCCATGTACCCTGGGATTCTGCCACGGAAGATGACTCACTTAATTAACACGCTGCTCCTGACCGTACACTTGG
AAGCAGAGGTTCCGTTCCTTTGGTTCTTGCCAAAGCTGCTTACGAAACCATATCTTGTGTTGGTGCCCATGGTGTGTAAAAATACAAGTTCATCATTTCCCTGATA
CAAATTATGACAGGCTAAATTTAGTGCCTCACCTGTCCTAACTAGCTTTCCCACCATCATGCCTGCCGCCGGGCTGTGGTCCTTTCTGAGGTCCCGTGTCCCTCTG
AGGACAGGGACACCCAGACACAGAGGTCAGTTGTCAGACGCGGCCACGGTGCTCGGGAGGCTGGCTGCAGACGGGCGATTCACCCTGGGTGTCTTCTTGAGT
CTTATTTTTTGTAGAGACGAG

Cg03363565 Vaginal 13% (6), Saliva 5% (4), Blood 44%(6%), Semen 2% (0)
cg09555736 Vaginal 20% , Saliva 26%, Blood 56%, Semen 6%
cg02102075 Vaginal 19% , Saliva 17%, Blood 52%, Semen 4%
cg06502279 Vaginal 18% (6%), Saliva 14% (5%), Blood 51% (8%), Semen 4% (1%), Chromosom 16

cg11682724 Vaginal 58% , Saliva 38%, Blood 36%, Semen 89%
not found, should be here after information from beatchip, false position on chip information ??
cg10716524 Vaginal 82% , Saliva 73%, Blood 78%, Semen 85%
not found, should be here after information from beatchip, false position on chip information ??
cg17645074 Vaginal 82% , Saliva 74%, Blood 78%, Semen 85%
not found, should be here after information from beatchip, false position on chip information ??
cg18493147 Vaginal 83% , Saliva 77%, Blood 76%, Semen 92%
not found, should be here after information from beatchip, false position on chip information ??
cg00814898 Vaginal 72% , Saliva 67%, Blood 81%, Semen 85%
not found, should be here after information from beatchip, false position on chip information ??

cg24595580 no HhaI cutting site

GCATCCCACCCCGTCTCGTCCCCTGAGAACAGCTGTGAGAAGCCGCCTATTTTTCATCGTTCTGCAACACACCTTTCCCTTCCTTGGGATATTTCTCCTCTTTCAGC
ATTGCCGCATCAGGGTTCCAAAGCCCCGTTTCTCTCCTAGTTCTTTCTTTGCTCAAGAAAAGCAGCTTGTCTGATAAACTAAGCTATGTATGATTCGCTGCCAGTA
CAGACTGAAAAACAAAATAAACTCCCAATTCACAGTCCTCCGTTGCGTAACAGTGAGCAGACGTTCTGAGAAATGCATCGTCAGGCAATTGCATCATTGTGCAA
ACAATATAGAGTGTACTTACCCAAACCTAAACAGCACAGCCTACTACACACCCAGGCTGTAGGCTGTGCTTCTAGACTACAAACCTGTACAGTGTGGTACTGAA
CACTGTAGGCAACTGTAACACAATGGTAAGTATTTGTGTATCTAAACATAGAAAAGATACAGTAAAAATATGGTATGAAAGATTGAAACTGGTGCACCTGCCTA
GGGCACCTACCGTAAGCTTGGAGCTTACGGGACTGAAAGTTGCTCTGGGTGAGTCAGTAAGTGAGTGGTGAGTAAA

cg24595580 Vaginal 14%, saliva6%, blood 46%, semen5% (6%, 3%, 8%, 1%)

cg04658021 no HhaI cutting site

Vaginal 12% (5%), Saliva 13% (6%), Blood 40% (6%), Semen 6% (2%); Chromosom 17, PER1

AGGGCTTCATATTTCCACTGCTTGGAAAAGAGAAAGCCAAACTCCAGGGGCCCAGACGGAACGTCATCTGTCTCATTCACTGTAGTCTCCAGAGCCCAGGACAG
GACCTGACACATGGCAAGTGATCGATAAACATGATTTTTTTTTTTTTAAACAAGGATAGGTTGGGGAGGTGGGAAAAGAGTGTCAACAGGAGAAAGGGGGCT
GGGAATTCTGATTCTCTAAAAGAGATGAGTAGTCCTAGAAAGAGAACAGGGGCACGATTTGCAGGCAATAAAATGTTAGTCTGGGCTCAGGAGAGCTGCAGT
TACACAGCCCCCTCCATCCAGGACCGTGATGAGTAGTTCCTCGCAGGGCTCCCTGGAAACGTGGGTAGTAGATGAACAGGCCTCCAAGCAGCTGCTGACATAA
TCATTGTGGGGGCACTCCCCTCACTGTCACGTGGCCTAAGCTTTCCTGAACAATGAGGGAAGGGTGAAGGGGAGGGCTCTGTGGAAAAGCTGAGACAAGATC
GTGTTATAAATTAACCCAGAAAAATCGAAACAGGAAAACAAAAGATTCAGGAGCCTGGAGGCCGGGCGCGGTGGCCTGTAATCCCAGC

cg04658021 Vaginal 12% (5%), Saliva 13% (6%), Blood 40% (6%), Semen 6% (2%); Chromosom 17, PER1
cg10708955 Vaginal 10% (5%), Saliva 8% (3%), Blood 24% (4%), Semen 3% (1%)
cg13759852 Vaginal 7% (2%), Saliva 8% (2%), Blood 19% (4%), Semen 6% (1%)
cg13433366 Vaginal 6% (2%), Saliva 6% (2%), Blood 12% (3%), Semen 5% (1%)

cg20155875 no cutting site

GTGTTATGGGTATTGGGTGATGTTGCAATACATATTTCGTATTGTGGATTGTGGTCAAGAAAAACTTGAAAGTCACTCAAATAACCAAGCTGTAGAGCTACTCG
ATTACATGATTACACATATGGGTTTTTCAAACAAAGGCATATTGTTCAGCACCGGGTGTTTTTAAACAGAAACATAAAGGGAATGGAAGCCAGGGTGGCTAATG
GTACATAATGGCCTTAATTACACCACTGAAGGATCAATATTAGCGTAGCTCAGTCACCAGCAGCCAAGGCACAGAAACTGGTACAGCAGGAAGTGGGAAACTG
GGAATCGGTCATCCCCGCCTGATGCTCAGGGTGCACATTCCAAAAAGATGATTCCATATGGTCCCCAGGGCTCGCTTCCATGAAAATAGGGAAAGAAACAGGC
GTTTGCCTGTAAGTCACAGAATCCTGCAGATGCCTGAAACACACTTCAGCCCAGCTCCCTCTCAAAGCCCAAATGTGAACATCGAAGTAAAACATTAAAAATCCA
CTCAAAAACAACAGGGTGGAGCTCCTGTTTCCTGGAGAGAAAGCAGTCTGCGCCCGGGAAAAGAGTCTTGGACTCCGAGAAG

cg20155875 Vaginal 21% (7%), Saliva 26% (7%), Blood 53% (5%), Semen 6% (1%), Chromosom 17, PRKAR1A (WIPI1)
cg25161129 kein Vaginal, Saliva 6% (6%), Blood 43% (7%), Semen 20% (6%)

cg27598107 no HhaI cutting site

CCTCCACGGCCGTCCGTCCAGGTACGGGGAAGCTGGAGCATCCGGGGACTAGGTAGGGGTGAGACGGACCACAGCCTTGACACCTTCGAGTCCCCCGCTCTG
CAGGACGCCGTGAGCGTCCTCCCTAAGTTGCACTTGGTTGTCAGAGTGTGTTGTGTAAGAAGGGGATGGCGGTGAGGTCTGCGTGTGTGTGTTTCTTGTGTGG
AGCAAGAGTTAACGTTGTTCAGGTCGTTTGAAGGAACTTGCCAGTTGTTCTGGAGGGGGGTTCTAGGTAGGTGTGTGACTTCCCGTGTGAATGGCTTTCAGGTT
TTCTATCCGGCTACGTTCTACAGGTTCAGGAGCGTCCCTCTTAGAGGTTAGGCTCAGGACCGTCAGTCTCCTTAACAAGGTAAGGGCTGAGGCCTTAACCACAC
TGGGGAGGGTAACTTGTTGCCCCTCCAGATAAAGGATGGTGCTAGGGAATGTAGCTACAAGCAAGTAGGCGCTGAGAAGACACTCATTATTTACAACAGTCAT
GCCCAGCACTTTACAGTTTATAAGCCCTTGACAGCAACTGGTCTTTTACCCTCCCTACAGTCTTGTGAGGAGGGTGCTGTCATCA

cg27598107 Vaginal 15% (6%), Saliva 10% (6%), Blood 42% (6%), Semen 4% (1
cg06880612 Vaginal 11% (4%), Saliva 7% (2%), Blood 24% (3%), Semen 5% (1%)

cg01092213 no HhaI cutting site

GCGCCTGGCCGTACTTTCATTTTCAATAAAACCCTTAATTCCTTCCTTGATTTGTTTGTGTGTTTCACTCTTGTCACCCAGTATGGAGTGCAATGGCGCGATCTCG
GCTCACTGCATCCTTCGCTGGGTTCAAGCAATTCTCCTGCCTCGGCCTGCCTAGTACCTGGGATTACAGGCACGCGACACCACGTCCAGCTAATTTTTTGTATTTT
TAGTAGTGACGGGGTTTCACCATGTTGGGCAGGCTGGTCTCGAACTCCTGACCTCAGGTGATCCATCCGCCTCGGCCTTCCAAAGTGCTGGGATTACAGGCGTG
AGCCACCACTCCCGGCCCAACCCCATGTTTTCTCAGGAACTGGAGTTCCCTTTAGGAAGCAAGTAACAGAGGTGCTGGGCTGCCCGGGCCTCTAGGTCGTCTTC
TTTCCCAGGCTCTGTGCTCTCACCAGAGTGCGCGTTCGGGAAGCAGGGAGAGGACCAGGAAAGACGCCTGAGACGGAGAGTGGGCACCGGCCGGGGGCAGG
CAGGTCCGGCCCCTGGGGGTCAGAGTGCAGCGCCGGTTTGAGGCAGTTCGGCCACTTAGCTGCGACTCGAAGTGAATGAG

cg01092213 Vaginal 16% (6%), Saliva 11% (4%), Blood 40% (7%), Semen 4% (1%),
cg02703728 Vaginal 14% (5%), Saliva 12% (3%), Blood 31% (7%), Semen 7% (1%)
cg02006203 Vaginal 4% (2%), Saliva 4% (1%), Blood 5% (2%), Semen 4% (1%)

cg19238415 no HhaI cutting site

GAAGGCGTGGTCTGGTTTGGAGAATTGGTGAGCGGGGTGTTTTAAAGCCACCTCTCCCGAACAATACAGAATCTGGTAGGTGCAGCTTCTGATTACATGGACC
AGGGAATCCTGGGTTGCTAATGACTTCATCCTCAGCTACCAACAGGAAGCTTCGTGGCTTTATGATCTCATCAGATTTCTTCATTATTTTCACGACAGAAGTGA
ACTTTTGTTTTAAGTTTATACATTACCGTGTTCCAGGTTATCTTCTTTGTTTTTACGAAAGTTCTACGTTCATGATTGAGCAAATAAGTGTGAAGGGTAGCTGGTCT
TTGTCTTAGATTGTCCATTGGGACGATTGTGAAAGAATGAGAAAAATTTGCTTCAAGAATAGGAATATAAGCTTTCTCAGAAAATTTTAGGCCCTTTGTTTTCAA

GACTTGGGAGTTAACAAAAATAATGTGATGTGCCAATTTGGAGCTAAGTAATTAAATGCTGAAACCTTTCAGAAATAGCCATAATTTTGTATTACATACAAGTAA
GCCATTTTAAGGAGTTTCATGAAAGACGTTTGGCACTTCACCCATTATTTCTTGCTGATGGTAATTTGACTATCTT

cg19238415 Vaginal 15% (6%), Saliva 10% (4%), Blood 40% (6%), Semen 4% (1%), Chromosom 11, QSER1

cg18464274 no HhaI cutting site

ATCTATACTCCCTCTTCCGACAAACCCTATACTTCTCTATATCAAAGCAGCATTTGCCAAGTCTTGGTAGGGAGCACTTCAGGTCATTCCCAGAGAGATGCCCATA
CATAAGGTGGAAGTGTCCATGTTGCAAGGCAGTGCAAACGCTCAGATTTCAAGCATTCATAATGCGTTGACAGAAACATTTTTTTTCTATTTAAATCTCATGTTG
ATTCCCACTCTGGCAGTGATTACGTCCTAAGTCATTACGTCGCTTTTTAAATACCAATTTGTCAAGAAAGGTAACTCCATCCCATCCAACAACAGACTTGAGCACG
CCAAGTATCGCTCATTTTTCTTAATTTGCTCTAAGTTTCTTAATAAACTGAGTGTTTCCAAAAGAAAATTTCACAACCCGCACAAATGCCATTTCCAAATTTAACTA
ACCATGGCTAATACTTAGAAGTGTAAGCGTTAACACAGGAAAATCAGCATGAGGGACAAAATACACACACATATACACTCACACACACACACACAATCCTTAAA
CACTGAACTAGGGCGCCAGCCTCATGTTTAAATATCTCAACTTGAACTCCACTGAAACAAGAGGAAATTCTCA

cg18464274 Vaginal 18% (6%), Saliva 14% (4%), Blood 40% (6%), Semen 5% (1%), Chromosom 12, GXYLT1

cg01840419 no HhaI cutting site

GGAGACCAGAAGCAGCATCTGACGAGGAGACAGCACTGGCGGTCCCGACACAACTGAATCGGCAGGATGAGGGCCCTTAGTGTTAGGCACCGGCGGAGCTG
CATCCCACCCCGTCTCGTCCCCTGAGAACAGCTGTGAGAAGCCGCCTATTTTTCATCGTTCTGCAACACACCTTTCCCTTCCTTGGGATATTTCTCCTCTTTCAGCA
TTGCCGCATCAGGGTTCCAAAGCCCCGTTTCTCTCCTAGTTCTTTCTTTGCTCAAGAAAAGCAGCTTGTCTGATAAACTAAGCTATGTATGATTCGCTGCCAGTAC
AGACTGAAAAACAAAATAAACTCCCAATTCACAGTCCTCCGTTGCGTAACAGTGAGCAGACGTTCTGAGAAATGCATCGTCAGGCAATTGCATCATTGTGCAAA
CAATATAGAGTGTACTTACCCAAACCTAAACAGCACAGCCTACTACACACCCAGGCTGTAGGCTGTGCTTCTAGACTACAAACCTGTACAGTGTGGTACTGAAC
ACTGTAGGCAACTGTAACACAATGGTAAGTATTTGTGTATCTAAACATAGAAAAGATACAGTAAAAATATGGTATGAAAGATTGAAACTGGTGCACCTGCCTAG
GGCACCTACCGTAAGCTTGGAGCTTACGGGACTGAAAGTTGCTCTGGGTGAGTCAGTAAGTGAGTGGTGAGTAAA

cg01840419 Vaginal 8% (3), Saliva 5% (1), Blood 29% (5), Semen 4% (1), Chromosom 15, FLJ42289

cg04111435 no HhaI cutting site

GGCTCAAGTGATCCTCCCTTCTCTGCCGTAGCAGAGACTACGGGCCTCTGCCACTGACTGCGGCCTTATTCTGAATTTTTAATATCTAATTTTATCATCCCCCATA
ATCACAGCCAACATTTTCTTATCTCCTGGCTCTTCTTCATCAGCTCAGCATTTTTTTTCCCTCTCTAAAATATGTCGTCTCTATCTGACAGTCTATTCGGAGGAAAAG
AGAACCTTAAGACCCAAGGTGGACCCTTCTCTGGCAACAGTAACGAGATAAGAATACGAAGCAGGTTCTGCGACTGCATTGCAATGTGATTGGAATGAGTAAT
CGTTTTTCATTCTGTATAGCATCTTTCTCCACCTACCAAATGGGGGGCCGTGCCATCTCTGCCTCTCGGTGAGGAGATATTACGACACAGCCAGTGAGATTCACA
AAGCACCTTGCAGAGTGGGCCTACTCTACACTCTGGTTTTCAGTTTTTATTGGAGTCACAGGTGGGAATGCATCAAACAAAAGATATAGGCTCTGGAATGTACG
GGAAAGAGAGTTTCCATAATGGAAGCCCAAAGACATTTCTACCGTTAAAATACATAATCTGCCGACCCTAAAACATTCACATAAAGAGGCAGGAGGGAATACT
ATCCCGCAGCTCCACGATTAGCCGCAGTCGTGGGCGGGGATTGGGGCTTGGATACGCCTTTTGCGTCTGGTC

cg04111435 Vaginal 7% (3), Saliva 7% (2), Blood 27% (7), Semen 5% (1), Chromosom 11, RTN3

cg25784219 no HhaI cutting site

AGTAGAGACAGCCTAGTCTGTTCATTGAGGGTGCAGTCAATGCACCCTTCGTAATGAGCACCAAGGTGATGAGTTTAAAATCCGAAAAGTTGTTGATTATGTTT
GCTTCCCAGCCCCGCTAGTGGTTTGTGTTGAATGTAAACATAATGAGGGAATAGGTTGAAGTCTGTACCCACTCCCTCCGTCTCCCGTTCGACAAACCCCAAACG
CCTGTCTGTCTCCTCCTCCCTGAAATCAGGAAGGGATCTTTTGATTTCTTCTCTGTTCTTTTCCCTAATGTGTCTTTAGCAGTGATCATTGGAGTATTACAATCTCA
CTGCCTGGTTTAGCATAGACCCTGGGGCCTGGAGGGAAGTCAGACCGGACATCAGTGCCCCAACTGAATAATCTGTGTCACTAGGGCTTTGAGCCCAGGATGT
GAGCGGAGGCAATTCCCGGTTTTGGAAGAAAATCAGACAGGAAACCAGTGCTAGGAAGAAGGAAGAGAACTCAGTCCAGCAGAGTGTAATAGGGCCTCATTA
CCGAAGCAAAAAGCAGCTGTTGGCTAATTCCTAGCATGCTGTAACACATGAGTATTTTGCAATTAGCAATAACCAGTGCACGGGGTTTCGCATAGACGCCTGCC
CATTCGGCCCGGCAGTGTGTGAGGCGCCTTTCAAACCGGAGGGATTGGCAAGGTAATGCCACTTGAGACAAGTTTC

cg25784219 Vaginal 7% (3), Saliva 5% (2), Blood 27% (4), Semen 6% (1), Chromosom 2, FOSL2

cg25365958 no HhaI cutting site

TGCACACGCAACGCGGAGGTTGAGTAATTTTAGGAACCAAGAGTAGTGTGTGTGCGTGTCTTAAGTGTGCGAGGATAAGGAGGAAGAGACCAGGGGGCTTTG
GATTTGTTCCCTCAAACTCCCTCAACTTCCTTCTCTCTTCCGATTCAAACAGAACCGTCTCACATCCCTCCAGGGATTCCACCCCGGAATCCCCCTCTCACTCAGAG
CTCTGGAAGCCTGTGTGGGCTGCCTTTTCCCTGGCGTCGAATTTTGACTCTCCACGCCCTGCCTCGATATTTGCTACTCCTTCTTGGTTGACATTGTGAGCCAGTC
TGAAAAGCCCCGTCCGAAATTTCTGGCCTCACCCATGTTCCGGTCAACGTGTTCGGATGCTGTTTTCCCAGGGACTCTCCGAGTCTGCTCAGGCCGGCCCTCTGC
CTGTTGAGAGCCGCTAGGTCAGGTGCCAGGGTTGGCTGTCCTTTTTCTTTTCTAGTATATACGCTCTCAGCTCCTTTGCCCAGAGTACCTCCTTTGCCCAGAGTAC
GTTTCTCGGCCTGATTCCTGACCTTTCAGAAGCGTCCTTCGACTCTGATCCCTCTGCATCTGCAGCTGTCTTTGCTTTCGTGAATTTGAACATGTCGTCAGGTTGCC
CATCTGCTCTTGTTTCTTCCATTGTGGTTATCACAGGGGCTTAAGCTAGTCTCCAAATTAAAAATACA

cg25365958 Vaginal 7% (3), Saliva 7% (2), Blood 26% (4), Semen 1% (1), Chromosom 13, DGKH

cg07152894 no HhaI cutting site
AGGAGTTTGGCTCAGATTTAATAGAGGACGTGTGCAGTCTTTCAGTGTTCCTCGGCCCACAAGCTTTAACCACTGTACAAACTGATTTAGAACACTAAAGCTCCA
CTGTTTGTTTAGTGACTTTATTGACTCAATTCCCGCCTCTCAGGCAGGTGGCCCCGGCAGGCAGCCCCGTCATTGTTCTGACTGGGTTCTCCTCAGTGACACCGT
GGCCTTCCTGCTGAGAGTTCCTTTCTTTATTTCATTTTTTCTGGTCCTTAAAAAGTTGGGCAATCAAGTCACAGTTACAGGTGTAGTGTTTCATTTTGAAAGAGGT
TTAATTCACTCCAGTCAGCATTTGCAGCGGTCAAATTGGGTTTGTGGTGTAATACGGACTTTTCCACTTTCCCCTTGGCTAAAGCGAAGGGCTGATTTCCTGGGT
GACCGTGAGGAGTGCCGTTACTATTGTGGCGTATTAACTCTTTCTGAACCTCGGGCCTCATTTGTCAGTGGCTTTCAAGGTGCCCTAATGTATAATTAAGATATA
CAGTAAGACAGATGCAAAGCGTTTGCAAACTTCCTGTGGTGCCTGGCTCCACTATTGATTTGGCCCAAAACAAAAAATATTTTCATTGTTCATTGCAAGAACAAC
ATCATTTATTAAACATACACTGTGCAAAGTACATTGTCTAGTGAGGTTTTTCCTTAGATGCATTGGCTTCTTTGAGCATCACCATAAACTTTTGTGCTGGGTATTA
CTGCTGTCTTTTATCAGACAGGGACAGTGAGGACCGGAAAGGTGAAGTGGCTTGCCCACAGTCC

cg07152894 Vaginal 6% (5), Saliva 5% (4), Blood 26% (5), Semen 1% (1), Chromosom 8,

Cg24124443 Lin etal 2016 not tested
GCCTGGGTGGGTGGGGACAGACAAGTAGATGACACGATGCTATTTGACACAGGAATGCAGCTAAGCCATGCATATATATATTATATCTTTCTTTAGTCAGGAAA
GAAGATTCTCCTTTGAGCAAATTTACTCCTTTTAAGAAGGCCGAGCTATTTGGAAAGATGCCCACCAGGATGGCAAAGTGGTGCTTGAGCCCTCCCTGCAGCAC
ACGCCCAGGACTTTGAAGCAGGGACAGGGTCCTCCTGGCTACTGAAAGGAATCGAGTTAACGCGCCCTAGTGGCAGGTGGCGAATGGCCGGAGATCCGGCCT
CCCAGTGGTGTCCACCGCCACGGGCCTCCGCTCGACAGGCTTATTTCCCTCCATGCTGGAGCAAACCCCGGCTGAAAGAGGGCCGCACTAATTGGTTTGCGTAC
CCGAAGTGGGAATAGAGAGGGAGGAAAACCATGTTT
CAGGTGTTACGGGGGGAAAAAAGTGAAATGCGAACCGCAGGGGAGAGAGCG

Cg24124443 **(Blood 19%, Semen 1%, Vaginal 4%, Saliva2%)**

Blood nur wenig methyliert

Cg01543184 (Lee 2015) not tested
Vaginal 21%, Saliva 9%, Blood 44%, Semen96% >> eher nicht Saliva Marker

Cg26285698 (Forat 2016) not tested
Vaginal 42%, Saliva 36%, Blood 10%(minimum 6%), Semen 84% eher nicht Blood Marker

cg19008649 BL_chr2_III not tested
AGGGTGGGAGAAAATGTTGAAATCAAGAAATTAAAAAACAAAAACAAAAACAAAAAAACCCCAAACCCTAACACCTCTTTTCTCCCACTTTGCCACCTCCTCTTC
GAAATTCGCAGGTTCTACGCGAAGTCCGGAGAAGGGTGAAAACGGAGGAGGGGTAATGAAAAGGAGCAAAAAAGGGAAAAAGCCCACACTGCTTTGCAGCT
CTTTCCTAGCTCTTTTCCCCTGCAGAAGTTTCCAAAGAGACTACGGGGCTCCGGGAGAGCAGGCGCTTTTAAATAGCCGGCCCCTGGCTGCCAGCCAGTTTGTA
GCTGCAATTTGAGCTTCCCAACACCCAACTCAGGCAAGGATGCCAAGGAGCTTTGCAGTACAAACTCACACGGGGTGGGGGTGGGGAGAGGCCTTCTAGACA
CAAGGGGGCTCCCCTTCGCTCCCTGAACAGCGCCCTCCTCCCCTCTGAACAGCGCCCTCCTCCCCCCGGAATGTAAGAAAGGGGCAAGGGGGACAGGCTGGGG
CTACCGAGAATGGGGAGGAGGTGGAGGGGCCAGTGGAAGACAGAGTTCTTCGGAGCAGGGTGAACACAATGAGGAGAGTAACTTCG

cg19008649Vaginal 7% (2%, max: 13%), Saliva 9% (2%, max: 16%), Blood 23% (4%, min:18%), Semen 3% (1%, max: 5%)
cg21032628 (Vaginal 5% (2), Saliva 6% (1), Blood 13% (2), Semen 4% (1))
cg20952768 (Vaginal 5% (1), Saliva 7% (1), Blood 8% (1), Semen 5% (1))
cg20419545 (Vaginal 7% (1), Saliva 10% (2), Blood 19% (4), Semen 4% (0))
cg19675223 (Vaginal 6% (1), Saliva 8% (2), Blood 15% (3), Semen 5% (1))

Produktlänge: 120 bp, 1 Nebenprodukt, 1 Schnittstelle

BL_chr2_III_for TM: 64°C, Länge: 20 bp
CCAAAGAGACTACGGGGCTC

BL_chr2_III_rev TM: 64°C, Länge: 21 bp
CTTGGCATCCTTGCCTGAGTT
Unspezifische Nebenprodukte!!!!

cg00664416 BL_Chr1_II not tested

CCTCTGCTTTCATGGCAGGTACAGGGTAGAGCAGCAGCCGTTCCTGCTTTTGCTGGCTGCAGAGAGGAAAGTGTGCCCGTGTTCCATGAAGGCCTCTGCTCTAT
TTGCAGTTGGAGGTAGTGTTGGGGGTTTAGAGGCTTTTCATCAAGCACCCAGCTGGAGGGAAGGTCCCAGGGCGCCTCCTTCGTAGGCTATGGAGGGGAGGC
TCACTTATTTCGGATTTCGTCACTGAATTTTGCTGGATTTTACAGATGGCCTCTGCTCAGGGCTCTTCTCCATGCGGGACGTTAGGGTTAGGGGTGCTTCTCTTCT
TCAGAGGTAGGCCTGTGGCTTCCAGGTGGGTGTGGCACTGTCCTCCCATTCCCTTTGTTCTACTACTTGCTGAGGCAGTCTAAAGCCTGGCTCCCTTCCGGCCTG
GCGCGCTACGGTTCCCGCAGGCGCTCCGCTTCAGCACCGCGGGCGTGGACAGCTCCGGGGCGCGAGGCTCTGGGACTCATCTGCAGGAAGTGAACCTGGCAG
GAGGCTTGCTTCAGGTTTTTAGGAACCATTTTTGCAATTGGAGATGTTTGTGAGGAAAAAAATCTCTGTTGAGGGGGGTACTGTGTGTCTCTGAAGTGGCCTCT
GTGTTGCGTCTCAGGCGTCCTGGTCCTGTCCTGGTACCCACCTGGCATGGCTGATGATAACGGGGAGCCCTCAGATGA

cg00664416 (Vaginal 7% (4%, max: 17%), Saliva 8% (3%, max: 17%), Blood 24% (5%, min: 14%), Semen 1% (1%, max: 2%)
cg24433287 (Vaginal 14% (4), Saliva 19% (5), Blood 27% (4), Semen 3% (1))
cg04219099 (Vaginal 22% (6), Saliva 31% (7), Blood 39% (5), Semen 6% (1))
cg02851558 (Vaginal 45% (3), Saliva 46% (5), Blood 50% (7), Semen 4% (2))

Produktlänge: 155 bp, 1 Nebenprodukt, 1 Schnittstelle

BL_chr1_II_for TM: 64°C, Länge: 20 bp
GACGTTAGGGTTAGGGGTGC

BL_chr1_II_rev TM: 62°C, Länge: 18 bp
CCTGCGGGAACCGTAGCG
Unspezifische Nebenprodukte!!!

BL_chr1_II_for TM: 64°C, Länge: 21 bp
TTGAGACCCAGTGGGGGTAAT

BL_chr1_II_rev TM: 64°C, Länge: 21 bp
TCATTTGTGGAGCGTTGGCCT

Bl_chr1_II_rev_new, TM64°C, 19nt
GGCATTCCCTCAGCGCCCT

PCR Product: 134 bp

cg14179389 BL_chr1_IV not tested

(Vaginal 6% (2%), Saliva 8% (4%), Blood 22% (4%), Semen 1% (0,3%), Chromosom 1, GFI1)
ACTTGCTGGAGCTATGGTTTTCGCCAAGTCAACTCACTGATTGTGGGACGGGTGGTGGTATCTTCTAAAACTAGCAAACATATTCTGGATATAACCGGCCCTTG
ACAGAATCCGGCTGGTCTGCTCATTCCTTCCCCCGGCCGGGACTCGAGACGCCCCCACCCAACGTGTGGTCACTTAGTTTGCCTGAAGCTGGATTGCTCGCTGG
AAATGAAACCCAGAGAGCAGGCCCCTGAGGCTAGGTTAACCCGGCAAAACGAAAAATGAAACAGGCCCCCAATCTGCGCCTGTGAAGACCAAGCCGCGGGCT
GAGATTTTCGTCCTGCCCCCTCCCTGCCGCCCAGCGCCTAGCTCCTCTGCCTGGCCCGTTCTGCCCTGTTCTCTGTTTGAGGTTTGGGGGTCTAGATTGCAGGAT
CCTAGCACTTTATAAAACAGTCAGGTCTGGTCAGAAAATGAAAGGTCAGCGTTGCCGCCAGTCGAAACGACTGGTTTGTTTTCCGTCACTGGCCAGCTCCGCGC
AAACGAATCTAAGCGACAAGCAGCTAGGGTCCTGCCGCCTGGGATGGAGGCAGCAGCCCCAGCGGGCAAGCTGTCCAAGTCC

cg14179389 (Vaginal 6% (2%), Saliva 8% (4%), Blood 22% (4%), Semen 1% (0,3%), Chromosom 1, GFI1)

Produktlänge: 167 bp, keine Nebenprodukte, 1 Schnittstelle

BL_chr1_IV_for TM: 64°C, Länge: 20 bp
GCGGGCTGAGATTTTCGTCC

BL_chr1_IV_rev TM: 64°C, Länge: 22 bp
GCAACGCTGACCTTTCATTTTC

cg10573476 BL_chr11_II not tested

Vaginal 8% (3%, max: 15%), Saliva 12% (3%, max: 18%), Blood 21% (3%, min 18%), Semen 4% (1%, max: 6%), Chromosom 11, BSCL2 (GNG3)
CGGCTCCCGGGGAAGTCCCGCCCCTCTCCGCCGGCCGCTTTTGTAGCCGTGGGAGGCGGGGCTTCAGCCCAGGGCGGGGTCCAGCACGGCGGGGCGGGGCG
GGACGGGGCGGAGCCTTCCCGAGCTGCGAGGTCGCTGTCTCCTTGCGCTCGCCACTGGCTCTCTCTGCGGCAGGTTTCCCTCCGGTTACCGTGACCAAAGGGG

AAAGACGCCTGCCTCCCTGCAGGCCCCAAGATGTGCCCCAGGGGATGCGCTGACTGCAGCCTCCGCTCGGCTCTCCCTTGAGACTGCAATCACAGCCCTCCGTG
CACACCTTCACCACAGGCCATTTCACCCGCGAAGCGGCCGAGTGAGGCGGTCATCCAGCTGGCGCTGATTTTTATCGCCCACAGCTCCTAGGACCTGTAGGCAT
CTAAGGCGGGGGCATTCGCCTAGAGCATGTTGCAGTTTGCGGCAACCTGGGCCTCTTGCGTTGTTGCGCAGGCAGATTAGTGCCCTGTTCCCAGCGTGGTAGC
ATTGTGGACCTCCTCTGACCCTAACTGGATACACTCTCCCTCAGAGTCCCCACAGCACATTATCTAGACTAGCTTTTGGCAGTCTT

cg10573476 Vaginal 8% (3%, max: 15%), Saliva 12% (3%, max: 18%), Blood 21% (3%, min 18%), Semen 4% (1%, max: 6%)
cg16200584 Vaginal 4% (1%), Saliva 6% (1%), Blood 6% (1%), Semen 4% (1%)
cg15878555 Vaginal 5% (1%), Saliva 6% (2%), Blood 7% (2%), Semen 6% (1%)
cg25230157 Vaginal keine Daten, Saliva 3% (2%), Blood 4% (1%), Semen 3% (1%)
cg18522549 Vaginal 1% (1%), Saliva 2% (1%), Blood 2% (0,4%), Semen 1% (0,3%)
cg13885159 Vaginal 4% (1%), Saliva 7% (1%), Blood 6% (1%), Semen 5% (1%)

Produktlänge: 179 bp, keine Nebenprodukte, 1 Schnittstelle
BL_chr11_II_for TM: 64°C, Länge: 20 bp
CTCGGCTCTCCCTTGAGACT

BL_chr11_II_rev TM: 64°C, Länge: 21 bp
ACTGCAACATGCTCTAGGCGA

cg24749947 BL_chr2_IV not tested

CTTATCATACATCCCTCAAAAAAGAAAGAAAAGCCCAATAAAATTGACAAGTCCATTGTATTACCTTGCTGCCTCTCTCCAAAAGCCAAATTCATGACAACTTTTT
AACCAAGACTTTTCTTCTGTTACTAAGTGGAACGTTTTCTTCTCTAAGTAGCTTTTCTCCCATGCAAGCAGCGTTCAAATCTCCAGGGGAAAGGAGGGGTGGGG
AGGGGGTGGGAGACTATCTGGCACAAACTGGACAGAGCCGAGCCAGTCTAGAACCCGACGGAAGTTACTGAATCCAGCGGCCCCAGAGGCCCACATCTCTTC
TTAGTGCGCCCGAGGTTCTAGCCTCACTGTTTTGGTTACAGTGTTGAAGGAAGGGTTTTAAACTTGGCAAAGTACCGTGCAACATCTGTAACCACATTTGCCTTC
TGGGGATCGGAAGATGAATCCCACATTCCTGGCTTTGAAGCTTTTTGAAACTGCAGCTAGGAAGAACCGAGGGAGGGAGGGAGTTGAGCAGAGTTTATTGGC
TGTCTCTCCAGCAGAAAATTCCTCTTGGAAGAGATGGATATTTTGAGGGTGGGGAAATTCGCCTATCTATCTCCTTACTCT

cg24749947 Vaginal 8% (2%, max: 12%), Saliva 8% (2%, max: 13%), Blood 21% (4%, min: 14%), Semen 4% (1%, max: 7%)
cg19286686 Vaginal 4% (2%), Saliva 4% (2%), Blood 9% (2%), Semen 3% (1%)
cg23967461 Vaginal 5% (2%), Saliva 4% (2%), Blood 15% (3%), Semen 2% (1%)
cg01943873 Vaginal 6% (1%), Saliva 5% (2%), Blood 15% (3%), Semen 3% (1%)

Produktlänge: 151 bp, keine Nebenprodukte, 1 Schnittstelle

BL_chr2_IV_for TM: 64°C, Länge: 21 bp
GTCTAGAACCCGACGGAAGTT

BL_chr2_IV_rev TM: 64°C, Länge: 22 bp
TGGTTACAGATGTTGCACGGTA

cg02676865 BL_chr10_I not tested

CCAAAGACTGTGTCATTATCGAGCTGCAGATCGTGTCTGCGGGAATCCGACTTGCAAAGCAGCGCAGGACTCTGGGAAAGAAATCTTTATTTTTCCCGCAGGG
GTTTTTAGAGCCTCTCGGCCGTCTGGGAGTCTATAAAAGATGGGGTGCGGGGTGAGAGTCACCCTTGGGATTGTTGGGATCCTTCCCAGCTTTGGGAGTGAGA
TCGCTCGCTGGTGATCCTCCAGGCCCGCGCAACGCTTTCTCACTTTCATGGTACACCCCGCCTGCCCTGGCATTTCGCCCCGGGAAGAGGTCTGTCGGTCATTTT
TCTGGGTCTTACACTCACTTCCTTCTTTGAGGGTGTTGCAAGGACCCGCTGGGAATAATATCGTAGACTTGAGAGCTCAAACTTGTCTGGATTCCAGATGGACCC
ATCTCCTACTCTGTGTCAAAGCCCAGATCCTGAGAGGTTGAACACCTTGTCCAAAGTCACACAGCTAGTTAGGAGCTCACCTGGGGCTAGAACTCAGCCTCCTCT
CCCACCCCATTGCCACATATCCCCTTATGTAAGAGGAGCCACAGGCAGAGTTGTTAATGAAGTGGAAACTCTAGTTGGAG

cg02676865 Vaginal 7% (3%, max: 13%), Saliva 6% (2%, max: 12%), Blood 21% (4%, min: 13%), Semen 4% (1%, max: 6%),
cg17296078 Vaginal 12% (4%, max 19%), Saliva 8% (3%, max: 23%), Blood 35% (7%, min: 24%), Semen 5% (1%, max: 7%)
cg09773756 Vaginal 0,4% (0,5%), Saliva 1% (1%), Blood 2% (1%), Semen 0,1% (0,2%)

Produktlänge:141 bp, keine Nebenprodukte, 1 Schnittstelle

BL_chr10_I_for TM: 64°C, Länge: 21 bp
GTGAGAGTCACCCTTGGGATT

BL_chr10_I_rev TM: 62°C, Länge: 19 bp

CTCTTCCCGGGGCGAAATG

cg20225915 BL_chr11_III not tested

CGCGACCCGTCCATCTGCCGAGCCGCCCGCGCAGCAGCGCTCGGCTTCCTGTTAGTCCGCGGGGTCGGAGCCGTACAGCCTCCCCCGCCCGGAAGGCCACGAA
ACCCGCCCTTTCCGAGGTTCTCGGGGCCCCGGGCCGCTCCGAGGCAGACCCGGCCCCAGGTGCGTTCCCAGGCCGGGGTGACGGGGGTACTCTTGCCGCTGCC
CCGCACCCACCCCTGCTACAAGCCGGAACAATTGCAGTGTCCTTTCCAAACCAGCCCAAACGGCCTCCTCCGGGAAGCCTGCCAGGACCTCCTCTCTGGGCCTG
CAAAGACCCTTCCTTTCGAGCCATCACTCTGGGCCAGAAGCAGTGAGCCTCCTGGCACTGTCTCCTTCAATCGCGCAGGGGAGAAGGAGGCGTTAAGAGATAA
AATATTGAGCGGGCGCGGTGGCTGACGCCTGTAATCCCAGCACTTTGGGAGGCCGAGGCGGGTGGATCACGAGGTCAGGAGTTTGAGACCAGCCTGGCCAAC
ATGGTGAAACCCCGTCTCTACTAAAAATACAAAAATTAGCCGGGCGTGGTGATGCGCGCCTGTAGTCCCAGCTACTCAGGAGGCTG

cg20225915 Vaginal 6% (2%, max: 10%), Saliva 5% (2%, max: 12%), Blood 21% (6%, min: 12%), Semen 4% (0,5%, max: 5%)
cg19480699 Vaginal 8% (1%), Saliva 9% (2%), Blood 12% (2%), Semen 7% (1%)

Produktlänge: 355 bp, keine Nebenprodukte, 1 Schnittstelle

BL_chr11_III_for TM: 64°C, Länge: 20 bp
CTCGGCTTCCTGTTAGTCCG

BL_chr11_III_rev TM: 64°C, Länge: 20 bp
TCTCCCCTGCGCGATTGAAG

cg00113675 not tested

GAGTCCAGGTGGACAGGGAGTCCAGTTCAGGGACGGAGATTCCGGGATGAAAAGTGAAGGGAGAGGGACAGGGCCCATGCCGAGGGTTTCTTCCTGGTTTC
TCAGACAGCTCCTGGGCCAAGACTCAGGGAAACACTGAGACAGAGCGCTTGGCACAGGAGGAGCGGGGTCAGGGCGAAGTCCCAGGGCCCCAGGCGTGGCT
CTCAGGGTCTCAGGCCCCGAAGGCGGTGTATGGATTGGGGATGCCCCGCCTTAGGGATTCGCCACCTCCGCAGTTTCTCTTCTTCTCACAACCTGCGACGGGTC
CTTCTTCCTTGATACTCACGAAGCGGACACAGTTCTCATTCCCACTAGGTGTCGGGTTTCTAGAGAAGCCAATCGGTGCCGCCGCGGTCCCGGTTCTAAAGTCCC
CACGCACCCACCGGGACTCAGATTCTCCCCAGACGCCGAGGATGGTGCTCATGGCGCCCCGAACCCTCCTCCTGCTGCTCTCAGGGGCCCTGGCCCTGACCCTG
ACCCAGACCTGGGCGCGTGAGTGCAGGGTCTGCAGGGAAATGGTCGGGAGGAGCGAGGGGCCCGCCCGGCGGGGGCGCAGGACCCG

cg00113675 Vaginal 15% (6%), Saliva 7% (3%), Blood 42% (7%), Semen 4% (1%);
cg26375284 Vaginal 3% (2%), Saliva 2% (4%), Blood 6% (2%), Semen 0,4% (0,4%)
cg11246563 Vaginal 8% (3%), Saliva 8% (2%), Blood 18% (3%), Semen 5% (1%)
cg10262387 Vaginal 8% (4%), Saliva 5% (2%), Blood 21% (5%), Semen 5% (1%)
cg23130010 Vaginal 8% (2%), Saliva 9% (4%), Blood 17% (3%), Semen 8% (1%)
cg07017114 Vaginal 8% (4%), Saliva 9% (3%), Blood 16% (6%), Semen 3% (1%)
cg25590181 Vaginal 14% (3%), Saliva 16% (5%), Blood 30% (4%), Semen 7% (1%)
cg00157477 Vaginal NV, Saliva 3% (2%), Blood 11% (5%), Semen 1% (1%)
cg01362455 Vaginal 12% (4%), Saliva 11% (3%), Blood 18% (4%), Semen 5% (1%)
cg01062395 Vaginal NV, Saliva 8% (7%), Blood 7% (2%), Semen 4% (1%)
cg02979711 Vaginal 31% (8%), Saliva 32% (11%), Blood 48% (7%), Semen 15% (4%)
cg03861427 Vaginal 33% (7%), Saliva 38% (7%), Blood 35% (5%), Semen 4% (2%)
cg13516209 Vaginal 11% (5%), Saliva 6% (6%), Blood 25% (8%), Semen 3% (2%)
cg23481825 Vaginal 27% (4%), Saliva 29% (3%), Blood 34% (3%), Semen 5% (1%)
cg23273834 Vaginal 43% (3%), Saliva 47% (4%), Blood 47% (2%), Semen 4% (3%)
cg20198242 Vaginal 15% (4%), Saliva 16% (7%), Blood 36% (6%), Semen 5% (2%)

cg17156633 not tested

GTGAGCCACCGCGCCCGGCCAGGGAGGATCGCTTGAGTCCTAGAGTTCAAGACCAGCCTGGGCAATACAGCGAGCCCCCCGCCGCAAAAAAAATTGTTTTAAC
ATTAGCTGGCTGTGATGGGGCAAGCCTGTACTTCCGGCTACTTGGGAGGCTGAGGTGAGAGGATCGCTAGAGCCTGAGAAGTCGAGGCTGCAGTGAGTTGTG
ATCGCACCACTGCATTCCAGCCTAGGAGAAGGGGCGAGACTGTCTCAAAAAAAAAAAGCAGATTATTAGGAAACGGTAGAAATGCCCAGAGGAACTAAAGCGG
ACCTATGTTTGCTGGCTTTGAATTCACTGCTGGGAACATGTGGAAACTGCTTCAGCAAAGGGGTGTCAGAAAGAATCCCCGGAACAAGTGTCAAAACACCCGT
GGTTCTTTGTGCTTGAGTTGGGGGCCCGGCCTAGAGAGCTAGCCCTAAACTGCTGCTACACTAAAAGCTAATAATCGTTCCTGCGCTACCTAAAGGTCAGGGAA
GGGATCTCAGACAAACGAGGCGACAACAGAGGGACTGGGGACACAGCCTCAGGATTCCCCAGTTGGGGCGGGGGAGGCAGGAGCGGT

cg17156633 Vaginal 15% (3%), Saliva 11% (3%), Blood 43% (9%), Semen 5% (1%)
cg09580965 Vaginal 3% (1%), Saliva 6% (2%), Blood 7% (1%), Semen 4% (1%)

cg01237807 Vaginal 6% (3%), Saliva 6% (2%), Blood 7% (2%), Semen 3% (1%)

cg14263391 not tested

TGACTGCAGATCGAGGGGCAGGCCATGTTGCTGGACACCACCCTGGCTGGCGTCCCTCTCGGCAGGGTGCTCTTTGCCCCATGGGGTGGGATCCAGAGCTGCA
GACAGGCCCCCAGGCTTGGCCAATGAACAGACCAGGTTCGGGGAGGGTGTTGGAAAAGAGTGGATGGGGTGGTTCCCCTTACCTTGCAGCCCCCAGGCCCTC
CCCCCTCCCTCCCAGGTGGTCGGGACTCTTGATCTTCGCTCGTGGTACTGTCTGTTCGGCTGTCTTCCCCGCCTCTCCCCAGGCACCTGCATCCTCCCTTGGCACC
TGCTGCCAGGCTAGGAAGGGCAAAAACAATCCCAGTTGGCGTAGTCAGGGAGTCTCCGCCCTCCTCCCAGGTTTCCTCCTCCCAGGCGCCTCCCCTGGACCCGC
CCCCATCTGCCCAAGATAATTTTAGTTTCCTTGGGCCTGGAATCTGGACACACAGGGCTCCCCCCCGCCTCTGACTTCTCTGTCCGAAGTCGGGACACCCTCCTAC
CACCTGTAGAGAAGCGGGAGTGGATCTGAAATAAAATCCAGGAATCTGGGGGTTCCTAGACGGAGCCAGACTTCGGAACG

cg14263391 Vaginal 27% (8%), Saliva 30% (8%), Blood 52% (4%), Semen 1% (1%)
cg03684977 Vaginal 48% (17%), Saliva 70% (15%), Blood 86% (2%), Semen 29% (5%)
cg08284496 Vaginal 28% (8%), Saliva 33% (8%), Blood 50% (3%), Semen 4% (1%)
cg24967701 Vaginal 14% (5%), Saliva 19% (5%), Blood 33% (5%), Semen 3% (1%)
cg23836594 kein Vaginal, Saliva 14% (3%), Blood 35% (4%), Semen 9% (1%)

cg25467652 not tested

GTTAGCAAGAACTCCCCCATCCTTGATGTCTCCTCCTAGGAATCTTCCATTCCTGCTCCCTCATTCTGCTCTGGCTATAAATCCCTCCTTGGTCCTTGTTGTATTCG
GTGTTGAGCCCAAACTCTCACTCCATGGCAATAACCCTACAGCAAGTCTTGAACAGTACTGGTTTAACAACAGTCCCAATAACGTTTTCTTTAACAACAGCAGTT
AGCTCATTTAATCCTCATAAGGATCATATGAGGTAGGTAGCCTAAATAGCCCATTTACACAGGGAAGTGGGAAGGAGAGAGGGTGCCCCCAAGGATGAGACA
CAAGCTGTTGCCACATCAGCACTTTGCGCCTGGACGGTCCCCCAGTTGTCCTGGCTGCAGGCAGTCCGAACAGTCCGCTTCCTGGAATATTAGCCAGGCAGGAT
ACGCCCTGGGCGACAGAAACTCCAACTTTCGCCAGCGGCCCCCAAGTACTGCCTGGGCGGTGGCGGGAGTCTGGAGCTTGGCTGAAGGCGCGAGCTGCCGAC
GCCGTCTCCTAGCAACCAAGCGGCGGTCACGTGACCCGCTCCAGCCCGTTTCACGTGGTCTTGAGCTGCGCTGGGGTTGGAGTGGCCGCAACGGGCGGGGCG
GGGCGGGGCCGGGCAAGTTTGTTCCCCGAGTTCGGAGCCTAGGAGCCCCCCGCGGCTGCGGCGCAGGTGCCCTCGGCCTGAGTCGGGATGGAGCTGCCTGCT
GTGAACCTGAAGGTGGCGACGGGCTGGGGGGAGGGTCCCCTTTGCGGGAGGAAGTGGGACATTTGCATTTTCCCGGA

cg25467652 Vaginal 8% (3), Saliva 6% (3), Blood 32% (6), Semen 4% (0)
cg17350448 Vaginal 6% (1), Saliva 7% (2), Blood 7% (1), Semen 4% (1)
cg06322619 Vaginal 1% (1), Saliva 1% (1), Blood 1% (1), Semen 6% (0)
cg19251740 Vaginal no, Saliva 2% (1), Blood 2% (1), Semen 1% (1)
cg17324198 Vaginal 1% (0), Saliva 1% (1), Blood 1% (1), Semen 1% (0)
cg13862210 Vaginal 0% (0), Saliva 1% (1), Blood 1% 0), Semen 0% (0)
cg20164720 Vaginal 2% (1), Saliva 4% (2), Blood 6% (2), Semen 3% (1)
cg15449870 Vaginal 10% (2), Saliva 8% (3), Blood 28% (6), Semen 8% (1)
cg15527643 Vaginal 13% (5), Saliva 12% (3), Blood 40% (7), Semen 3% (1)
cg21826784 Vaginal 9% (5), Saliva 4% (2), Blood 35% (3), Semen 3% (1)
cg08945450 Vaginal 11% (5), Saliva 7% (3), Blood 38% (6), Semen 4% (1)
cg08425760 **Vaginal 20% (7)!!!!!,** Saliva 13% (6), Blood 45% (7), Semen 5% (1)
cg04444949 Vaginal no, Saliva 9% (3), Blood 31% (7), Semen 4% (1)

cg17452301 not tested

AAGCAGGGGAGGGTCAGATGCGCCGTGGCCTTGCCCTGGCAGTTCCTCACAGGTGGGGGTGCCCTGTCGAAGGCCACGGGCCCCTGGCTGCTGGGAGTGACT
CCGCGGCTGAGGTTTGGATTCGGGGAGAGCAGTTGCGCCCAGCTGTGCGATTTAAGAACAGTTTCAGGCCAGGGAGCCCTGAGTCATGGGGGCGTGAAGAG
GTACAAGCTGTGGGAGCCGTGGGCACATCCCCCGGGAGCCCTGGCTTCCTGGGTGGGGGGCGCAGGGCAGGGCGGGGCGGTTCCTCCTGAGAGGCACCTGT
GCACGGGCTGCAGGGACCCATGCGGTGCAGATGAGCCTCCTGGACACCGCGTCACATGGCAGGGAGCTGTCGCTGCCAGATCAATGATTAGGCATGAATCAG
GCTGTGGCCTCGCTGCGCTCCTGCAGGGCTTTCTTTTGAAACTGGGAGCAGGGAAAGGCTCCTCTTGTAAAGGCCCAACTTTTTCCATCCGTGGAGCCCAGATT
TTTGCCTGGTGACATGAATTAGCAGAGAGGACCGTGTTCCTTCCACAATTATTTCGCAGACGTTATCTCTTCATTAGATGTGCCAGAAGCCATTTATCCACAAGT
CACGTGATTTAACATATGGCTCCCGTGTGGTTCCTGCCCGCTCCGTGTGGGCTACCGCTGGGAGTGCACGGGCAGGATGGAGGGACACTCGGGGGGTGAGAA
TCGCCACCTGGGAGCTGCCATCCCCTCCACCATCGGCCTCAGCTTCCTCTGGCGGGCAGGGTTGGGCTTGGTCCTGGTCAGCAG

cg17452301 Vaginal 8% (3), Saliva 8% (3)(max über min von Blood), Blood 25% (5), Semen 9% (2), Chromosom 10, PWWP2B
cg02372082 Vaginal no, Saliva 2% (1), Blood 2% (1), Semen 1% (1)
cg24646285 Vaginal 4% (1), Saliva 6% (2), Blood 7% (1), Semen 5% (1)
cg11579421 Vaginal 15% (6), Saliva 10% (3), Blood 37% (6), Semen 41% (5)
cg25303150 Vaginal 11% (6), Saliva 4% (2), Blood 33% (4), Semen 49% (6)
cg24085039 Vaginal 10% (4), Saliva 13% (2), Blood 26% (3), Semen 63% (7)

cg27518898 not tested

CGTACGTGTGCCACAGATTGGTGCACCTGCCGGCATCTCTAGGATTGCACCAACTCCCTCTAGCTCTTCTTGGTGGCCTGCAGGGGCCTAGGGCGCATTCACTTC
CCTCTGCTTGACTTTACGTGGTGTTCTAGCGTCAGACATCAGGCGATGCCGTGCATTACCCAAGACACTGGACAGCTCCGGATCTTCCCGTCTAACCACTAATCA
AGTTAAAAAGAGGAAATTAACGCTTTCTTAGTAGTCTCTGAAAGAAACTAGTTATTTGCGTAAAGATGACTTGGTTTTGTGTTGTTTATCCCTGGGGAGAAGGG
TAAAGAAGACCCTCAAACTGGATTTGCTGCTGAAGTGCTTTTAACTCACCTGTGCGGAGAGCGGACTTCAAGAGAGGGCGTGCCGGTGCTCCGGACAAAACCT
GGGGAATACTGAAAGCCACCACCACCACCGTCATTCCAGCATGCAGATCTCAGTCCTGGGTTGGTGTTGTAGGGCATTTATTTATTTGACTTGACGGCGCCTTTC
AAAGCTTGCGAGGATCCCTTCCTCTTCCTCGTGTGCATCTGCCAAGCCTGTGCCCCGCGCCTCGCGGGTGGACCCGGGCTGGTGGGCGGGGCCTGCAGCGGGG
TGGGAGCCCGGTCGCCCGGCCCCTCCCCACCCCGCCCCGCCCATCTCCGCTGGTTCCCGGAAGCCGCCGCGGACAAGCTCTCCCGGGCGCGGGCGGGGGTCGT
GTGCTTGGAGGAAGCCGCGGAACCCCCAGCGTCCGTCCATGGCGTGGAGCCTTGGGAGCTGGCTGGGTGGCTG

cg27518898 Vaginal 7% (3), Saliva 6% (2), Blood 24% (5), Semen 4% (1), Chromosom 21, IL10RB
cg01928078 Vaginal 8% (2), Saliva 9% (3), Blood 21% (4), Semen 5% (1)
cg12829325 Vaginal 4% (2), Saliva 3% (1), Blood 7% (2), Semen 2% (1)
cg10683055 Vaginal 9% (2), Saliva 6% (3), Blood 18% (5), Semen 6% (1)
cg14607407 Vaginal 4% (1), Saliva 5% (1), Blood 6% (1), Semen 4% (1)
cg00765428 Vaginal 1% (1), Saliva 3% (1), Blood 2% (1), Semen 1% (1)
cg07325233 Vaginal 1% (1), Saliva 5% (2), Blood 3% (1), Semen 1% (0)

cg27424995 not tested

GGCCGGGTAGCCACGTCTGGCCGGGCGGGGGCCAAGCCGGCCGCCCCTTCCCGCGCCCCGGGCCCGGCGGAATGCGTGGCAGCCCCGACCGGGGGCTATTTT
GAGCGGGAGGGGGAGCGGCCCGGCCTCGGTGTTTTCGGCTTTTTCCTGGCCCCCGGCCCGCCAGGCCGGGCCCTCTGCTGCCCGCTGAATGGGAGGGGGGGGCG
GGGTCACGTGGCGGGGGGAGGGGAGGGCCGTCGCGATCCGGGTCGGGCTCGGGCTCTGCCCCGGCCCGGGGACTGGAGGCGCCCGGCAGCGGTCCGGACC
TGCGCTCTCGGACGCGGCTGAGTGGCGGGGTTATTAATAGCGTGCCTGGCTCGGGCAGTGGCTCCAGGGCGCCCGCTACCTGCACGCCCCTCCTGCAGAGAGC
ACTGGCGGCCCGGCGCTCCCTCCGCAGCGGCGGACTGAGGCCTAACTGGATAGCGCCACCGTGAACCGGCGGCCAATCACAGGCCTCGATTTCCTTACCTGTA
AATTGGGGTTGAGCTACCCGGCTTTATAGGGTAGGTGCCATATAAAGGTTTTGTTTGCTGTGTGCCTCTCCTTGGGCAAAACGCTGTCACAATTGGGGGCCTCC
GTTTTCCTTATTAAAACGAAGGGGTCTGAAAAGGCCCTCTGCAGGGAAAGCGTCCGGACATCTGAACAATGCGTTTTCACTGACCAC

cg27424995 Vaginal 7% (5), Saliva 1% (1), Blood 23% (7), Semen 1% (1), Chromosom 1, AHDC1
cg27543538 Vaginal 20% (6), Saliva 13% (3), Blood 39% (5), Semen 6% (1)
cg15072832 Vaginal 13% (3), Saliva 13% (3), Blood 25% (4), Semen 8% (1)
cg11657615 Vaginal 12% (3%), Saliva 14% (3%), Blood 31% (6%), Semen 7% (1%)
cg00420361 Vaginal keine Daten, Saliva 7% (2%), Blood 9% (3%), Semen 5% (1%)

cg01022567 not tested

GCACGAAGCACGTACGCCCCCTGGTGGCCGTGGCGGGGACTGCCGCGCGGACTCCGCAGCCCCGCCCCACCCCGCCCGCCCAGCCCAATCCCACCCGCTCCGC
CCTGCTCGCCCCTGCCTGCTCACCTACTGGGCTGCGCTGTCCACGCCTGGAGCCCGGGCAGCTTCAGTTCCGCGGAGGACAGGTGTAACCCACAGAGTGATTCC
TTTACCGGGGCCTGCTAGGCTCCGGCCCACGAGGCAGGACTGGCGGCGTTCTCACGTCCTGGAAGTCAAGGCCCCAAGGGGACCGAGGCGCTGCCCAGGCTC
CCCTGACTGCCAGCTCCGGCCTGGCCACCCAGTCCGCCCCCAGGGACCGGACTGGGTGCGCCGTCCACCCTTCCAGTACCTGGAACATTCTCCGCGGATTGGGA
GGTTCTGGGTTCTGCCCTAGATTCCCAAGCAAAGTTGCTCCTTTGCTCTTGTCACTTGCCAGCTTCGTGGTTTTTGTTTTTTGTTTTTGAGACGGAGTCTCGCTTTG
TCGCCCAGCCTGGAGTGCTGTGGCGCAATCTCGGCTCACTGCAACCTCTACCTCCCCAGGTTCAGGCGATTCTCCTGACTC

cg01022567 Vaginal 8% (4%), Saliva 11% (3%), Blood 21% (2%), Semen 2% (0,4%), Chromosom 7
cg23616524 Vaginal 28% (7%), Saliva 41% (6%), Blood 51% (5%), Semen 9% (1%)

cg05127217 not tested

TGCTGGGCAACAGTGCGTGCGGCCTGGCCTCTTTGCGCCCGGCGGGCAGCGCGCGTCTGGGGCAGGCTCCGCGCGGGGCTCGGGCACAGGTCCCCGCTGCCG
CAGCCCTACCGAGTGAAGCACTGAGCGCGGCGCCGCTTCGCCATCACCTGGCTCTGGCCCTCTCACCCTAGCGTAGGGGGGCACTGGCCAGGCAGTGAGGACC
CTGAGATCCATGCCCTGGGAGACAGAGGTTGTCTCCTGTCCATGCACCCGCGCGCCTGCCTTCTAACCTTCATACCGTTGGGCAGATTAATTCACTGGACTAGGC
CTCTGTTCCGTGGGAGTTGAAAAGATTTGACCTGGAAGAAACCCTAGCGATTATTCAGTTAAGCCGCAACCCCACCCCCTGTTTTATATAGGTAAGGGTGCTTCA
CGGTGAGGACTCGTCGCGTGCCTGGTGCTTTACATCAGTCCCCAGAATAGTCCTCGTAGTTTCTCTCTTACTGTTCCCATAGTATGGGTGAGGACTCTGAGGCTC
GGAGAGGTTGTGAATTTAGCCAGAATCACACAGCTTGTAACTGGTGGAGATGAGGACACTGAGACTCGATTAAATCACTCG

cg05127217 Vaginal 7% (2%), Saliva 11% (3%), Blood 21% (3%), Semen 4% (1%), Chromosom 11, TTC12

cg13356117 Vaginal 1% (0,4%), Saliva 1% (1%), Blood 2% (1%), Semen 1% (0,3%)
cg16302655 Vaginal 32% (19%), Saliva 48% (13%), Blood 66% (6%), Semen 1% (1%)

cg03784054 not tested

AGCGAGGATTAGATCTGAGTCCTTGCCCCTGCGACACCCACCTCCGTGGTATTGGAGGGAGGAGAGGAATGATAATTGGGGGCTGAGGTACAAAATACTCCA
GCGGGACAAGGAGGACTGGCATAGCTCCTTCTTCCACCGCCCTCTCCGGCTTGGGTCCTGTGACTGCGGCGGGAGAGAGGAGCAAGGTGAGCCGCCCCAGGG
GTCTGGGTTTGGGTGCGGGTGAGGGGAGGACTCAGGACTCTGGGCTGGGTCCCGCTTGAGAGAAGCGGGCGGGGAGGGCGCGGGTGAGTCACGGCGGCCC
CTCTGCGTCGCCGCTCGGACTCAGCCCTGGCCAGGCCCGGCCCGGGGGAGGCCGCCTGGCTCCCAGCGCGCGGCCAGGGAATGTAACCAGCTGACCAGGGAG
GGGGCCGGCAGGAAGTGAGGCCACCCGACAGCCTCCGCCCGGTTCCGGGGTGGGGGGTCCGGGGCTGTGCCGCTCCCCCTCCCTCCTATACGCTTGGGCAGC
TGCGAGATGACGTCAGCCGCCGGGTTTGGGGGCAGTTGCCATGGAGACAAGGACAGGCTCAGAAGGGACCCCCTTCTCCCTTGCAGCCGCCCC

cg03784054 Vaginal 7% (5%), Saliva 4% (3%), Blood 20% (5%), Semen 1% (1%), Chromosom 16, FAM65A (RIPOR1)

Semen marker

Sperm_chr2_I cg17610929 (Park, Lee, Lin)

ATCAGGTGGTGGCTGGGTGCTGGAGTCAGGAGTCTCAGAGGCCAGGAGGTGGGGGGATAGAGTTGGCTGACCCTTCATGGGGTGTGGGCCAGGAAAGTGG
CAAAGAATGAGCTGAGGACCCTGGGCACGGCACCCTTCCCTGGGTGGCCAGGAAGTGGGGAGATCTGGGGACCCAGTGAGCGGCTAGGGTGCAGCAGGAGT
TTGGGGGATAGCCCCAGTCTTGGGATCTCTGTCCTGGGCTGGGGACTGCCCCCTCCCCTGGCCTGGCTCCTGACGCCCGTGCTGCCGGTGAAACGCTGTTGACA
TGTCCTGAATTATTAAGCGTGGGGAGGGCTCCGGAGCACATGCTGAGCGGAGCGGCTGGGGCTGCGCGGCGTGGCGGAGCAGCGCTCGCTCCCTCGCTCACT
CGCTCGCTCGCAGGGACACACGCAGGGGCTGACAGCTGTGCTGGTGCTGATAAGGGAAGCCACAAGGAGACGATCGAGGAGAGAGACAAGCGGCAGCAGA
GGCAGCAGCGGCAGAGGCAGCACCAGGGCTGCGGAGCTGCTGGGAGTGGGAGTGACTCCCCCACCTCGGGCCCCCACCCTGTCCCTGTCCTCTT

cg24743778 kein Vaginal, Saliva 3% (2%), Blood 3% (1%), Semen 97% (1%)
cg17610929 Vaginal 0,5% (1%), Saliva 1% (1%), Blood 1% (1%), Semen 98% (1%)
cg09726982 Vaginal 1% (1%), Saliva 1% (1%), Blood 2% (1%), Semen 93% (4%)
cg18844301 Vaginal 5% (2%), Saliva 7% (1%), Blood 8% (1%), Semen 94% (4%)
cg10515731 Vaginal 1% (0,4%), Saliva 1% (0,4%), Blood 1% (0,4%), Semen 95% (5%)
cg19210770 Vaginal 4% (2%), Saliva 8% (3%), Blood 6% (2%), Semen 97% (2%)
cg18395327 Vaginal 2% (1%), Saliva 2% (2%), Blood 3% (1%), Semen 97% (2%)
cg12046414 Vaginal 3% (1%), Saliva 6% (2%), Blood 5% (2%), Semen 84% (2%)
cg21767952 Vaginal 23% (7%), Saliva 26% (5%), Blood 16% (3%), Semen 78% (3%)

Sp_chr2_I_forw: TM: 68°C, Länge: 20 bp

Sp_chr2_I_rev: TM: 66°C, Länge: 20 bp

Alternative Primer
Sp_chr2_I_forw_varia
GCGGCGTGGCGGAGCAGC

Sp_chr2_I_rev FAM
TGCTGCCTCTGCTGCCGCTT

PCR Produkt 144bp
Products on target templates

Sp_chr2_I_forw
GCACATGCTGAGCGGAGCGG
PCR Produkt 174bp
Sp_chr2_I_rev FAM
TGCTGCCTCTGCTGCCGCTT

Sperm_chr5_I Cg07485723 (Lin 2016)

GACCCCCACCAGGCCAGGTCTGGGTAACGGGAGCCCCGCCTCCTAGCCCTGGCCACGAGGCATAGTGAAGCTGGAATTTGTGGGAGTTTTTTGTTCTGTCTGCC
ACTCAACCACAGGCTCCAAGTGGGGAGGGACCCCTGGATGTGTTTCCTGCTACCGTATCCCAAATGCCGAAGCGCCTCGTACATAACGAGAACACAGTAAATA
CCCGCTGAATGGATCAACAACGGTGAAGGGAACGATTCTCTGAATGAACGTCCATCCTGCTGGCTCTCGTGCCCGGCCAGGGAACACTAGAGGGCGCGCTTGA
CCTGCCGAAGGCTGCCTCCAGCCCTGGCGGACTCTTGCGCTGGACTCCCGCCCTCGGGCCACAGCCTGGGCGATCAGCCAGGACGCTGGCCATTGAGGATGCA
GGCCCAAAAGGGGCGCCTTCAGCCTCACAGGCTTCCCTGGCTGGCATTGGTAATGGACAGAGCAGTGGGAACGGTCCCTGAACCCAGT

Cg07485723 (Vagina1% Saliva 1% Blood 2% Semen 96%)
cg06449486 (Vagina1% Saliva 1% Blood 2% Semen 90%)
cg05975527 (Vagina1% Saliva 1% Blood 2% Semen 87%)

Sperm_chr5_I_forw: TM: 64°C, Länge: 21 bp

Sperm_chr5_I_rev: TM: 64°C, Länge: 21 bp

Sperm_chr7_I cg05261336 (Lin 2016)

CAAAGTCCTAGGGTGTCTGAGGTCAGAGTGCATCGGGTAGCTCCTGCTGCATAACAAGCGGGGGAGGGGGTGGCCCATGGCTCTGGACTGCTGGGCCACAG
GGGTGATGAGGTGAGGCTTGGAGGAGTCTGTAGGATCCTAACTTTGATGACTATTCAGGGTTTGAGGCTGCACTCAGCTTTATCGCCTGTGAAACCCACGCAG
CCAGCAGGGGTACGAGTGCTTCTGAGGTCCCAGTGGGCGGGGGGTGGCCTGACCCAGACAGGGCAGGGGGACGGCAGCTGGAGCGCGTGGAGAGAGCGCCC
CGTTCCCTGGGAGCCACTGGAGGGCGCATGGCACACGGCGCCCGGCCAGGGCCTGACTGTGACTGGAGCAAGAGCCGAGGGACTGGACGGACGGCAGGTG
GCGCCACCTGATCTGTGCAGGGACAGGCAGGGACAGGCAGGCAGAGAAAGAGCCCAGCTGGAGGCTGCCAGCGCTCCAGGGAGAGGAGTGGGAGCGGAC
AGCGCCGGCCCGGGCTGGAAGGTCAGCTTGAGGCTCTGGGATGTGAACACTGGGCACCGAGTCATCCAGAAATAAATCCCAGGCACGGTGGGAGGCC

cg05261336 (Vagina1% Saliva 1% Blood 1% Semen 95%)
cg24187253 (Vagina15% Saliva 18% Blood 12% Semen 87%)
cg17120143 (Vagina8% Saliva 8% Blood 8% Semen 79%)

Sp_chr7_SE-I_f aus Lin et al. 64°C, 21nt

GAGGCTGCACTCAGCTTTATC

Sp_chr7_SE-I_r aus Lin et al. 64°C, 20(1)nt

FAM-(A)GCTCTTGCTCCAGTCACAGT

Sp_chr7_IB_r , 66°C, 21nt

CTTGCTCCAGTCACAGTCAGG

Sperm_chr7_I_For: TM: 68°C, Länge: 20 bp FAM

Sperm_chr7_I_rev: TM: 68°C, Länge: 20 bp

Sperm_chr8_I Cg26763284 (Lee 2015)

AGTGGGCTGCTCGGGCCCCTCTTCTGGTGGGGACAGCAGGGGGAGGCTGGGAGGGAGGGAGGGTGTGTGCCGAGGCCGCGGGCAGTGCTGGGGGGCTGC
GGCTTGGCCAGGCGTGCGGCTGGCCCCTCCTCCCGGCGGGTGTGGCCGCGGGGACGCAGGGGTTAAGGCCAACTAACCTTGAGCGTCAGTAGCCCTGCGCAG
GGACAGCTGAGCCGGCCGGGTGGGGAGCGAGGGTGGGGAGGCGCCGAGGGCCGGACCCGCGGAGGGAGGAGGAGAGGAGGCGGGCGGGAGCTGTTGTG
GGGCTGCCGCCGCGTATGGGGCGGTCCTGCGGGCAGGGCAGGCTCCGGTCCCGGCGCTCCCCCTCGCTCGCCCTGCACGCAGGGAGGGGGCTGTCTAGCCTG
GAGCAGCCGGTTCCCAGCGCAGGAAGGGCAGCCCTGGCGGCTCCGCGTGGCGGCTATGGAGCCCTCGGGCAGCCTGTTTCCCTCCCTGGTGGTTGTGGGTCA
CGTTGTCACCCTGGCCGCTGTGTGGCACTGGCGCAGGGGACGTCGGTGGGCGCAGGACGAGCAAGGTAAGCCCCGCTGGTGGCTGCCAGGGACTCGAG

Cg26763284 (Vagina1% Saliva 1% Blood 2% Semen 98%)

cg10319399 (Vagina4% Saliva 11% Blood 5% Semen 97%)
cg23715237 (Vagina5% Saliva 5% Blood 7% Semen 93%)
cg21550172 (Vagina3% Saliva 4% Blood 4% Semen 91%)
cg20154947(Vagina3% Saliva 3% Blood 5% Semen 80%)

Sperm_chr8_I_forw: TM: 62°C, Länge: 20 bp

Sperm_chr8_I_rev: TM: 56°C, Länge: 16 bp

Sperm_chr8_I_rev_alter TM:66°C, Länge: 19 bp

Sp_chr_8_IB_f, 18nt, 64°C
GGTGGGGAGCGAGGGTGG

Sp_chr_8_IB_r, 19nt, 66°C FAM
GGAACCGGCTGCTCCAGGC

JT.1 (Muc1.1) Teschner

AGGGCGGGGAAGTGGAGTGGGAGACCTAGGGGTGGGCTTCCCGACCTTGCTGTACAGGACCTCGACCTAGCTGGCTTTGTTCCCCATCCCCACGTTAGTTGTT
GCCCTGAGGCTAAAACTAGAGCCCAGGGGCCCCAAGTTCCAGACTGCCCCTCCCCCCTCCCCCGGAGCCAGGGAGTGGTTGGTGAAAGGGGGAGGCCAGCTG
GAGAACAAACGGGTAGTCAGGGGGTTGAGCGATTAGAGCCCTTGTACCCTACCCAGGAATGGTTGGGGAGGAGGAGGAAGAGGTAGGAGGTAGGGGAGGG
GGCGGGGTTTTGTCACCTGTCACCTGCTCCGGCTGTGCCTAGGGCGGGCGGGCGGGGAGTGGGGGGACCGGTATAAAGCGGTAGGCGCCTGTGCCCGCTCC
ACCTCTCAAGCAGCCAGCGCCTGCCTGAATCTGTTCTGCCCCCTCCCCACCCATTTCACCACCACCATGACACCGGGCACCCAGTCTCCTTTCTTCCTGCTGCTGC
TCCTCACAGTGCTTACAGGTGAGGGGCACGAGGTGGGGAGTGGGCTGCCCTGCTTAGGTGGTCTTCGTGGTCTTTCTGTGGGTTTTGCTCCCTGGCAGATGGC
ACCATGAAGTTAAGGTAAGAATTGCAGACAGAGGCTGCCCTGTCTGTGCCAGAAGGAGGGAGAGGCTAAGGACAGGCTGAGAAGAGTTGCCCCCAACCCTG
AGAGTGGGTACCAGGGGCAAGCAAATGTCCTGTAGAGAAGTCTAGGGGGAAGAGAGTAGGGAGAGGGAAGGCTTAAGAGGGGAAGAAATGCAGGGGCCA
TGAGCCAAGGCCTATGGGCAGAGAGAAGGAGGCTGCTGCAGGGAAG………

GCGC = Position 155162682 hg 17 in NCBI
Primerpaar 1 Primerpaar 2 = aktuell
HhaI Bindesequenz
CpGs
Protein-Startcodon (Sequenz ist anti reverse zur NCBI, Start Codon ist korrekt!!)
cg22500132 (Vaginal 15%, Saliva 3%, Blood 6 %, Semen 69%)
cg22531371 (Vaginal 16%, Saliva 7%, Blood 11 %, Semen 49%)
cg18804777 (nicht mehr zu sehen, überall methyliert)
TGAGGTGACATCGTGGGCTGGCGGGGTGGTGGAGCCCAGGGCTGGCCTGGTGACTGGGAC[CG]AGGTGACATCCTGTCCCCAGGTGGCAGCTGAACCTGAA
GCTGGTTCCGTGGCCGG

L6 Wasserstrom

GGGACAGCCCTGGATGTCTAGTTCCTCAGGCACGTGTGCACACATGTGTGCTGCGTAGCACTGGCACCCCATTCCTGTTCTGCCCCAGGTAGCTGGTGCATTCC
CCGGTGCTCACCGGCTCGACCCCCCAGCAGCACGAGAGACCTCACAGAGGGAGTCACACTAACGTGGTCGGCGCTCCAGAGCGAAACCCCAACCACTATGCTC
ACAGCCAGGACCGAGCAGGCTGGGCCAACGGCAGTCCCTGCCCAGCGCCCGGCTCCCTCCGAGTGGCCAGCAGCGCCCTCTGGTGGAGACTGGCTCGGCCTC
CGCGGCACTGCATTCCCACGGCAGTGGTCCATCTAGTCCCCAAGTCCTAGAGGAGGCCCCTCTCTCTCCCTCAGCCCTGGCAGGGTCCTTGGCGCCCATTCTCCA
CACCTGCACTCCTTGGCCCTCCAGGAATTACCGGCCTCAGGGGCGCTCCTGGAAGCAGAGAAGGGGAGCTGCCTGGACCTTCCTCAGAGGCACAGCCTTCACA
GAGGCACAGGGGCTTTGGAGCAGAGGAGGCTGCCACTTATCAGCCAGACGGCCTGGGCTCAATTCCCAGCTGCAGGGCCCTGG

cg10043090 HCCA2 (Vaginal 24%, Saliva 45%, Blood 57 %, Semen 93%)
cg15639910 (Vaginal 7%, Saliva 7%, Blood 8 %, Semen 80%)
cg08029231(Vaginal 34%, Saliva 45%, Blood 57 %, Semen 85%)

L7 Wasserstrom

GAGGCTAAGGGGGCACACCTGGGGGTAGGAGGAGTCTGGGAAGGAAACTCAGCTGGAAATGGTTTAGGCCTCAGAGCCCACATCCTGTGGGAGGGGCTGTC
AAGGGGCCTGCTGTCCCGGTCCTGTCGCAGTCCTCAAAGATGCTAGAGTGACAGTCCTCTAGGGGTAGAGATGGTCGTCCTCCCAGGAGAAGGTGGCCCGGA
GACTTGGAGGTGGGATCAATCCTGCCAGTCCTGGATCAGGAGGCCTCTGTCGGGCGCCGCCCCCCTTCCTCCTCCATCAGCAACAGGCGGCGCCGGCCAGCCT
CATAGTCAGCCTCATCCACACTGACCAGCAGGCGAACAGCCTCCCGGCCCACAGCCTCTCGCAGGGCCTCAGTCAGGAACACGCCCCGCAGGGCCTGCAGCAG

GGCGCCACTCAGGTAGTCGCCCCAGAAGGCGTCCAGATAGGAGAGCTCTGAGAACTTGATGTCACAAACCACAGAGCCCAGGTCCCTTGAGCGCAGCACTGC
GGTGGCCTGCCCAAACACGTCCAGCTGCCGCGCCAGCGCCTGGGGCCGCCGGGATGCCACGCCCTGCTCCAAGGCTGGCCCATGCTCGCA

cg11721464 (**Vaginal 49**% (Min 4 % max 79%), Saliva 6%, Blood 6 %, Semen 89%)
cg14013103 (Vaginal 51%, Saliva 21%, Blood 11 %, Semen 78%)
cg14189583 (Vaginal 44%, Saliva 11%, Blood 10 %, Semen 80%)

L7_for
AGCCTCATCCACACTGACCAG
L7_rev
GTTTCTTTCAGAGCTCTCCTATCTGGAC

Cg23521140 (Park 2014) no HhaI cutting site

AGAGTTTGATAGAGAGGAACCGGGAGAAGAGAGAAGGCATGGGATGATGAGGAGGGAGAAAGTCCCCTCCACTTCTGCCTCACCTCCCAGGAAGGAAACTA
GTCCATAACAGGGAGGCAGGAAATGTAACCTCCCACCTGGAGTTTCCCCTGTTGACAGATGGGGGAGGAAGTCATGGGGATGAGTCAGAAACTCTTTGAGGG
AGGCTTTGCTTGAGAGCTGCCTATGCAAATTGTCTTGGGCTTCCTTTTCTGCCACAGAGGAAACAGACTAAACCTAAAGGCTTTGCTGGTACACAGAAGCCAAA
AGGAGATCAAGTGTGACTATTCCGAGGTCAGGTTTCTCGCTCGATGCCTGAAGGAGGTTCAACACCCCCTCCTTCCTCTGCCTACTCCTTCTGGCCTTCTGCCCTC
CACCCAATTCTCTGTCTCACCCACAAATCCTACCCAATGCAGAAAAGTACTCCATACTGTGGTTCTTCATTTCTCTTTATTTCATAACCATATTTCTCATCTCTCTTCC
CTGGGACTTCCTCAAATGTCCTTCTTTTGAAGGAGAGAGATGAGGAGGAGGCAAGAGGCATACCTAGGGAATTCCCTGGAGTTCAACTGAAACTTGTTGCAGT
TCTGTTTCTAAGTCACCATTTCCCTGAGGGCAGCACTTTCAGAACCTACTGGCAACTGAAGTCTAGATCCCCACTC

Cg23521140 (Vaginal 1%, Saliva 1%, Blood 1%, Semen 97%)

cg23324953 no HhaI cutting site

CGCAGGGGCCCGCCCTGCCTGGCGGGGGTGCCCGAGGGGAGCCCTGCTACCTTTCTTGCCCTCAGAGGCCCTGAGCACAGCCAGGTACAGGTTGTCCTCCGAG
CTGGTTCTCTTTCGGCCCAGGCCCTCGGGCTGCGGCACGCGGAGCTGGTGCTGAGACATGCTGCCCCCACACCTTCGTCGCCCGGACCCTCGGCCTCAGGCACG
GTGCTCTGGGCAGCCCCGTGTGGCACACAGGCAGCTGAAGGCTGGCGGCCCCACGAGACGCTCACTCGGCACAGGCTCAGCTCAGAGCCCCAGTCGGTGCGG
CCACTCCCTGCCTGCTGTACTCCCCGGCGAGGCCGGCCCGCCCCCGAGGCCCCACCCTGCCCAGGCCGCCGCCAGGGAGGGGAGTGTCCCGGCGGGAAGGAG
CAAGCAGCCCCCTCCCCCACAGACACGCCCTCGGCCCGCCAGAAACAGGAAACCGGGGTTTCCTCACCCACCCCGACCACTGCAGCCCCCTAGGGGGACCTGG
GGACACGGGGAGCTGGCACTTCACTGCACACCAAATGGTGCCAGGACCGCAGAGGAACACAGGCCGTCCCCACAGCCACCCAGAAGG

cg23324953 kein Vaginal, 1%, 1%, 97% (1%, 1%, 2%)

L5 Wasserstrom not tested

>seq_24464 701 bp
GAGACATAGGACAGACAATGACTACCAAGCAGCCTTCAGCCCCACTGCCTGAATCCCCGCAGGGGTGTGTAGGAGAGAGGAAGAGCTTTGCTGCCTCACCCCT
GGGTGATGTTCAGGCTAGCCTGGTGCGCGAGAGGAGGGGAATGGCAGGTCCCCGCTTCGCGTACCTTGGAAATAAGCTCATCGTGTTTGGCCAGGTGTGCAC
GGCAGTGGACACAGCTGTAAGTGCGGTGACAGCGGGGCAGATAGCTGCGGAAAGTCTTGGTGGGGAGGCAGGCAGGGCCCGGACCGGGGTCACAGCTGGG
CATGACGGGCTGGAGGACAATGCCCTGGTGGGCTGGAGGGGCTGGCGCCGTGCAGCCCCCGCAGAGACGGTCGCAGGTGAAGCAGCGGAGCAGGTTGGCT
AGAGCCGTGTTTCAGGGCAGGAGAAGTGTTGGGGGGCTGCCCGGCCAGGGCCCCCCCAGACGAGAACCAGATAGAAATAGAAGTCACCTGGGAAGAGGGG
AAAGGACATCAGGGGAGCTGCGACCTCAGGAGGACCCCTCCTGAGGGAATGGAGGAAAAGGGAATCAGCTCACCCATACCTCTACTTTCCCCATCACCATCGA
CCCCCCCACAGCATCCAGTTGCATTGTCGCCCTGTCCCCTGGATCCCCATTCTGCTCTGGCAAACCTGGCAGCCGATCCAGGCTTCTGGCGCC

cg27368025 (Vaginal 24%, Saliva 21%, Blood 38%, Semen 87%)
cg10366093 (Vaginal 6%, Saliva 4%, Blood 14%, Semen 98%)
cg15363887 (Vaginal 8%, Saliva 12%, Blood 12%, Semen 91%)
cg03631561 (Vaginal 10%, Saliva 11%, Blood 19%, Semen 88%)
cg11324116 (Vaginal 12%, Saliva 13%, Blood 25%, Semen 92%)

L5 Forward
TAGCTGCGGAAAGTCTTGGTG

L5 Reverse
GCTTCACCTGCGACCGTCTC

Produkt: 142bp
Blast: keine Nebenprodukte

Cg05656364 (Forat 2016) not tested

(**Vagina 20%** Saliva 3% Blood 3% Semen 89%) semen?

cg14061378 not tested

CCTCAGAGTGTGAAATGTGGTGACAGCGGTGCCCGCTGGCTGCTTGCAGGATCCTGGCAGTGACCCGCGGGCAGGGCCCCCCGGACGGCCCCCTGCGGAGG
GCGCAGCCTTGCAGGTGGGGTCGGAGCACAGGGTCCCAGGGTCCCAGGCCCCACTTCCCAACCAAGCCAGGTCCTCTCTCCGCCCCTCTCCTCGAATCTTAAGA
GCCTTTGGAGAGAAGCCACGCAGGGCGGGACGCACAGGCTGTCAGGGTGGGAGGGGTGCGTGTCCCACGTGCAGGGAGCCCCGCCCAAGGCTGGCAGCAG
CAGGGTAGAGGCCACTGGTCCGGGGCAGCTTCCGCGGGGAAACACCGCCGCGCTGTGAGCATTGGGGGAACTGCAGCCGCCGCCCCATCTGCCGGCTGCGG
GTCGTTGGGGGGGCCCCATGCCAACGGCCACGGAGGCCAAGGACCCCCGGAACCCCTCGGCCTTTGTCCCAGGAGAGGCTCCTCACACCCTCGGTGCCAGGG
CCCTGGGAGCCCGTGAGACCAGGGTTCTCCCTGCTCAGACTCTTCCTGTGGCCTGGAGGGGACAGTGGGGGTGACAGGCACTGACCACCAGG

cg14061378 kein Vaginal, Saliva 1% (1%), Blood 2% (1%), Semen 97% (2%), Chromosom 9, LOC105376325

cg02543772 not tested

CCCCTTGAAAACTCCCAGCCCCCTTTGTCCAGATGGGGATGGAGGTGGCCAGGCTGCCCCGTTGATTGTGTGCCGAGGAGCCCTCCCCGGGAAGGCTGTGATT
TATACGCGCAGGCTTGTCACGGGGTGAAAGGAAGGGCCACTTTTTCATTTTGATCCAATGTTAGGTTTGAAAGCCACCCACTGCTGTAAACTCAGCTGGATCCG
CGGGCCGTGATTAAACACATTGCCCGCTTTGTTGCCGAGATGGTGTTTCGGAAGGCGCTGTGAATGCACTTCCCTTTGCGGGGCTCACACAGACAAGATGTGTG
TTGCAAGGATGAGGCGCCTGCTCGGCCTCCAGCCCAGGGCCGGGAAGGGAGAAGGTGCTGTGCGTCGCTGCCTGTGTCGCCCGCGGCTCTCCTCCCAGCGAC
AGACTCCAGACCTCTATGCTGTCTGCTTTTCCTTTTTTACCTCCCCTGTCCCCTACCCGGTTTTCTAGCCTTTGAGGGAACTGTTCAGACTGTTGGATTCAGACTGT
TAGTGCCATTTCCCCCCAGCGTGGACAACTGCACAGTGCGAACTTGCTGACTTTCCTTAGCTGCTCCTGCAGAATGGGGTT

cg02543772 kein Vaginal, Saliva 2% (1%), Blood 3% (1%), Semen 97% (1%), Chromosom 12, TMEM132C
cg25254825 Vaginal 6% (1%), Saliva 9% (1%), Blood 9% (1%), Semen 85% (2%)
cg06613286 Vaginal 21% (4%), Saliva 31% (9%), Blood 23% (5%), Semen 91% (2%)

cg04560810 not tested

TTGAAGCTGTCCTGTGCAGCCGTACCCAACCAAGAACCCTTGGGGACCTGGGTTCCCCTGTCCCCGGAACCCACCCCAGGGAGGACCTTAGTCAACAAAGTGG
AAAACAGAGCTCAGGGAGTGGGGACAGGGCAGCCAGCAGGCACCCCCTCGCCTCTGGGTGCCAGGGGCCCTAGGTTTGGCGTTCTGCCAGGCTGATCTTGAG
CCTACCTTCTGCCTCCCGGACACTGGCTGCTCTGGATGATGACGAGGAGAGAAGGCCCGGCGCTACGAGCAGCCAAGGGCAGCTTCTCTGAGTGCAGCCTGAA
AATGAGATGTTAACAACGATTGGTTTCTCCACAACACTCTAGCCCTGGCGTTTCTCCAAACCGCAGCTTTACTGGTGCTGGGAAGGAGGGTATTTCCTGTTCCTC
AGGAAGGGAGGTAGGAACCTTCCCTCAAAGCCCTGATGAGCTGGGCCTGAGTCCTGGGGTCAGGGGTGAGGTTTGAGAAAGCCTCCATTCCATTGCCCTTTGC
TACCCCCAAACCAAGGGTGTTTAGAAACACTCACCGCAGTCACAGCTCTGAACAGCGGGTTCCCCTCAAGCCACCACGATCAAAC

cg04560810 kein Vaginal, Saliva 1% (1%), Blood 2% (1%), Semen 97% (2%), Chromosom 5, STING1 (TMEM173)
cg03317505 Vaginal 5% (1%), Saliva 5% (2%), Blood 8% (2%), Semen 84% (2%)
cg14655316 Vaginal 5% (1%), Saliva 6% (2%), Blood 9% (2%), Semen 88% (3%)
cg16532438 Vaginal 5% (1%), Saliva 5% (2%), Blood 7% (2%), Semen 87% (3%)

Non Semen Marker

Non semen marker L3 Wasserstrom et al.

chromosom 11 Gen: ARAP1 (ArfGAP with RhoGAP domain, ankyrin repeat and PH domain 1)
TTCCCCCCACCCCGCCTGGGCTGCTGTGCCTCTCATGGTGGGTGACAGCAGTCCCTGTGCCCTCCAAGTCAATGCAGGCCAGGCATTCAACCAGGGTAAGGAAC
TCTCGGTCTTCCAGGGCCCCTGGCTCTGTATGGAGCGGCGATGGGAATGAGAAGCAGAGGCAGGCAACTGGAGCCTGTGGGTTAGGGTAAGCCTAGTGCAAG
TGCCACAAGGGAAGGGCGCAGGAGTCCGGAGGGATGGGTGGAGGAGGAGGAGGCTGGGCACGGGCTGCAGGGCCCACCTGAGGCCTCCAGCCAGGTTAG
GCGCTGGGCGCGAGTGAAGAGCCCATCAGGCAGGTCGCGCAGGAAGCGCTTGAGCGCCGAGGAAACATCATCCACGTGCTGCTCGCCCTCCTTGAGGTGCAC
AGAGCGCGCATCCTGCCGCAGGCTCTCCAGCAGCCGCTGTGTCTTCGATGTCTGCCCACACTTGCGGTAGATGCCCTCGGAGGTCAGGCCTAGGGAGGGGCGG
GGCCAAGCGTTCGGGGCCTGAGGCATAGAGTCATGGGGCGGGGCCGCGCAGCTCTGGGGCGGGAGGCGGCTCTCCGGGAGGGGCGGGGCTGGCACCCTAG
GGGCAGGGCTCACCGCACTGCGTGATGTAGTCCACACAGCGGTACACGATCACCGGGATATCCGAGTCCCCAAGCTGCTGCTCCGACAGCG

cg16617141 (Vaginal 83%, Saliva 88%, Blood 95 %, Semen 10% (max 29))
cg00238848 (Vaginal 79%, Saliva 85%, Blood 92 %, Semen 3% (max 12))
cg27090029 (Vaginal 86%, Saliva 93%, Blood 96 %, Semen 9% (max 32))

L3_for
GAGGCCTCCAGCCAGGTTAG
L3_rev
TGCACCTCAAGGAGGGCGAG

Produkt 121 bp
Blast: ein mögliches Nebenprodukt ca.3000bp

Non semen marker L4 Wasserstrom et al.

Chromosom 4 Gen SLC34A2 (solute carrier family 34 member 2)
CAGGCCCCTCACCTGTCCAACCTCTTGTGTTGCAGATCGCCCTGTGCCACTTTTTCTTCAACATCTCCGGCATCTTGCTGTGGTACCCGATCCCGTTCACTCGCCTG
CCCATCCGCATGGCCAAGGGGCTGGGCAACATCTCTGCCAAGTATCGCTGGTTCGCCGTCTTCTACCTGATCATCTTCTTCTTCCTGATCCCGCTGACGGTGTTTG
GCCTCTCGCTGGCCGGCTGGCGGGTGCTGGTTGGTGTCGGGGTTCCCGTCGTCTTCATCATCATCCTGGTACTGTGCCTCCGACTCCTGCAGTCTCGCTGCCCAC
GCGTCCTGCCGAAGAAACTCCAGAACTGGAACTTCCTGCCGCTGTGGATGCGCTCGCTGAAGCCCTGGGATGCCGTCGTCTCCAAGTTCACCGGCTGCTTCCAG
ATGCGCTGCTGCTGCTGCTGCCGCGTGTGCTGCCGCGCGTGCTGCTTGCTGTGTGACTGCCCCAAGTGCTGCCGCTGCAGCAAGTGCTGCGAGGACTTGGAGG
AGGCGCAGGAGGGGCAGGATGTCCCTGTCAAGGCTCCTGAGACCTTTGATAACATAACCATTAGCAGAGAGGCTCAGGGTGAGGTCCCTGCCTCGGACTCAA
AGACCGAATGCACGGCCTTGTAGGGGACGCCCCAGATTGTCAGGGATGGGGGGATGGTCCTTGAGTTTTGCATG

cg19241593 (Vaginal 87%, Saliva 84%, Blood 87 %, Semen 8% (max 19))
cg16363447 (Vaginal 98%, Saliva 97%, Blood 97 %, Semen 4% (max 20))

L4_for
CTGCAGTCTCGCTGCCCAC
L4_rev
AGCAGCAGCGCATCTGGAAG

Produkt: 136bp
Blast: 2 mögliche Nebenprodukte bei 250 bp und 111bp

L4_187
L4_187_for 66°C 20nt
GTCGGGGTTCCCGTCGTCTT
L4_rev 64°C 20nt
AGCAGCAGCGCATCTGGAAG

Produkt 187 bp

Cg17621389 (Lee 2015)

(Vagina 97% Saliva 96% Blood 96% Semen 1%) **Nicht Sperm?? Chr. 6**

Cg22407458 (Forat 2016)

(Vagina 13% Saliva 8% Blood 4% Semen 1%) non semen ?

Controls

PCR control 1 (L1) Wasserstrom et al.

auf Chromosom 19 Gen: *SAFB2 (scaffold attachment factor B2)?*
GCAAAGAGCAACTCCGGGTCAGCTATCTGGGCCGCAAGCCCAAAAGGCGGAGGAGGAACCCCGCACAACTTGGGTCTCTCTGGGTCCAAATGACAAAGGAGA
GGGAGGCTGTGAGCAGGAGCTCCAGAATTGGGGTCAAGGACCAGTTCCAAAATGGCAACTCAACTCGACAATGCCAAACCTTCGACTTTAAAGTTATATGATG
GGACAAATTAGAACAGAGAGGCTGCACTTGGCCCGGCCTCGGTGAAGGGCTGGCCACCGCTGGTGAGGAGGAACAGCAGCCAGCCTGGGGAGATTCAACAG
GTCCCTCAGTGCCTGCCACCTGCCGGGTCCAGGACTGGGTACCCACAAGCTCCCTCCCTATGCTCAAGTTGTTTGTTCCAGTTGCGGAAACAGACAATTCCCAAC
CCCTTAATATTTTGCATTTAGCCCCTGCTTTCAGCGAGGTGCGACACAAACGCAGGATGAGCTGGCAAGGAGGTGGGGCAGCGCATTAGGAAGGGCGCTCAG

GAAGGTGATGTCTCAGGCAGACCCAAAATGACAACAAGGGGCCGGCCACGAGAAGATCTGGGGGACCCGGGTGGACAGCTTCGTAAAGGCCAAAGCCCTGG
GGCAAGAACAAGCACCGCCCCCTTTCCTGTAAATGCTCTGGAGTCCGCCAGACACCCATTCTCCGGAGGAGGAAACTGAGGCACAGA

ApK1_for
CCTCAGTGCCTGCCACCTG

ApK1_rev
GAATTGTCTGTTTCCGCAACTG
Produkt: 97 bp
Blast: ein paar mögliche weitere Produkte, ein bei 369bp Länge

PCR control 2/ L8/
auf Chromosom 2, *Gen: SAFB2 (scaffold attachment factor B2)?*
AAAACTGTCTTCTATTTGTGCCACAATTTTGTATCACAGTTAAGGACAATTCAAAACCATCACATTGTCTGGTTAGAGGAACAGACTTTTTTGGGAAAGGAAACA
CAAAACTTTGTCTGCAAAAGAAGAGAAGCACTGGTTCTGCAGCCTCCCTCCTGCAATCAGGCTGGGACAGACTATTCTGCTGGACATGCAAGATGCACCGTAAT
CCAGGGGAGAAAAATCCTTCCCCTTGGCAACCTCTTCTTTCATAGGCAAGTATGAGAAAAGCATCACTGTTAGTGGGGGAATACCCGGAACACTTTGGGCCAA
GACATTCTGGCTTTTTGTGTCACCTGTTTAGAATTATTTTCTGGGAACCTAGAACCCTTGCCTGGTGTTGTACCCATGCTCCCAAACAATGGGGATTTATAGTCTG
TCCATTCTCCTTTCCTCTATCTCCTATTTGACTGGGGGAACCCCTGAGCCATGGGAAACTGACAAGAGTAGAAAGTCCACGACTGGACCCTTCTAACTCATTGTTC
AGGCAACGTGGTGAGTAATTAAATATTTCCAACCCAGTTTTGACTCAAGGGATTTAAGATGTTAACCACATCAATAT

ApK2_for
TGAGAAAAGCATCACTGTTAGTG

Apk2_rev
GGACAGACTATAAATCCCCATTG

Produkt: 160 bp

(Keine GCGC Schnittstelle)
Blast: kein Nebenprodukte

DC (Digestion control L2) Wasserstrom et al.

chromosom 22, gen BCL2L13 (BCL2 like 13
CGGCATCTGGGCGGCTCAAGGCCCGCGGCCTTCCCTAGGCGGGCTCCAGCCCACTCCCCGGGACTGCCGCCGCGGCTTCGTAAGACCCAACCAAGCCCCTCAC
CTGCTTTTGCACGTCAGCATCGCTGAGAGCCATGGCGAGAGCAATGCTAGGCCGGTGAACAGTAGGCTCGAGTTTAGGTTTGAAAGGTGAGGTGAGAGAAAT
CGGCAAAGGGAACCCCTGCGCAGATCTCGGGTTCCTTTACTTTATAACCGCGGGTTCCGGTTCCTGCCAGGTGACTGCACAGTTCATCCTCATGACCCTTCTCAG
CCAATGGGAAGAGAGCGACGCCCAGGAAGTCCCGCCCTGTCCCGGCCTGTGGGCGCGTCCTCGGGTCCTTCTACGTCGCTGACTCGTGACCTGACCGGTATTTT
TTTCCTAAACTGGGATCTTGGGTAGGAGGAAGAAAAGATAAGGAGTTCCTCTATCTGAAATTAGTCGGGCTGTTTTGAGGAGTACTGGTTAGGTATATTGGAG
AATGTGCCTTTATTGGAATTTTTCGCATAATTACACAGGGTTATGGGCTTAGGGGTACATGATTGTTGGCCGGGCGTGGTGGCTCACGCCTGCAATCCGGAGAC
TTTGGGAGACCAAGGCGGGTGTATCGCTTGACCTCAAGTTCGAGACCAGGCTGGACAACATAGAGGAACCCCGTCTCTAC
VdK_for
GAGCGACGCCCAGGAAGTC
Vdk_rev
ATCCCAGTTTAGGAAAAAAATACC
Kein Treffer auf Chip

Produkt: 108bp
Blast: keine Nebenprodukte

PCR control PC Lin et al.

GTGGGTGACCCCGTCACCGGAGTCCATCACGATGCCAGTGGTACGGCCAGAGGCGTACAGGGATAGCACAGCCTGGATAGCAACGTA
CATGGCTGGGGTGTTGAAGGTCTCAAACATGATCTGTAAGGCAGAGATACACCATGTCACACTGGGGAAGCCACTGGGGACAGCCAG
GCCAGACGGGGGACATGCAGAAAGTGCAAAGAACACGGCTAAGTGTGCTGGGGTCTTGGGATGGGGAGTCTGTTCAGACCTACTGTG
CACCTACTTAATACACACTCCAAGGCCGCTTTACACCAGCCTCATGGCCTTGTCACACGAGCCAGTGTTAGTACCTACACCCACAAC
ACTGTCTTAGACACCTAGTCAGAGAGACAAACACCAGAAAAAGAGCTCATCTGGGAAAAAGCAAATAGAACCTGCAGAGTTCCAAAG
GAGACTCAGGTCAGAGAAGAGAGTCCTACGGAAAACGGCAGAAGAGAGAACCAGTGAGAAAGGGCGCAGCTCCGGGAGGCCAGGAAG

GAGGGAGGCGGCCACCAGAAGAGGTAGCGGGCCACTCACCTGGGTCATCTTCTCGCGGTTGGCCTTGGGGTTCAGGGGGGCCTCGGT
CAGCAGCACGGGGTGCTCCTCGGGAGCCACACGCAGCTCATTGTAGAAGGTGTGGTGCCAGATTTTCTCCATGTCGTCCCAGTTGGT
GACGATGCCGTGCTCGATGGGGTACTTCAGGGTGAGGATGCCTCTCTTGCTCTGGGCCTCGTCGCCCACATAGGAATCCTTCTGACC
CATGCCCACCATCACGCC

PC for
6FAM-CGCTTTACACCAGCCTCAT

PC rev
TCTCACTGGTTCTCTCTTCTGC

Digestion Control (DC) Lin et al.
>seq_23269 503 bp
GGCCGCGTGCGAAGAGCCAGCGGCAGCCCGAGCGCGCCTGCGCGGGGACGGGCGGGACCGCCACGAGGGGTGGGGCATCCCCGGAGG
GCGGAGCGTGGGTGAGGGGCGGGGCATCCGTCGCCGGAAGCCTGTACCGTATTTCATGGACTCCTAGGCGCCATCGATTTTAAGACG
CGCTCGCATTCCACGCACCACTGAGCAGAACGTTCGAACCGGACAAAAGTGCGAGTCAAGGCGCCCTTCCTTGTCTCGGGCTACGCT
TCTGACTGCAGCCAGGCAGCTGTGGGCCGGGGCCTTGGCGGCTGGAGGAGGGGCGTGAGCTGGTCTCGCCCACGCACCTGGCGCGCG
TACGGGCCGCGCCCTTTCTCCTTTATTGGCCGAGATCCCCACGCTGCGCGCGGGAGGCTGGCGCGCGGGGTGGAGAAGGGCGTGAGG
AAGCGGTCCAAGTAGCAGGAGGGCGGTGCAGGGCCCGGAGGGAGCAGCCCAGGGCGGAGGCGGCGG

DC_ for
ATTCCACGCACCACTGAG
DC_rev
TCTCCTTTATTGGCCGAGAT
