# Supplementary material for: Expanding the scope of methylation-sensitive restriction enzyme (MSRE) PCR for forensic identification of body fluids through the novel use of methylation-dependent restriction enzymes (MDRE) and the combination of autosomal and Y-chromosomal markers
Source: Int J Legal Med. 2023 Oct 24;138(2):375–93. doi: 10.1007/s00414-023-03097-9 (PMC10861701; doi:10.1007/s00414-023-03097-9)
Supplement: Supplementary file 4 — Supplementary file4. Sequences of Y-chromosomal markers (PDF 823 KB) [file 414_2023_3097_MOESM4_ESM.pdf]

Inhaltsverzeichnis

Saliva 2
SA_chrY_I cg27355713 2
SA_chrY_II cg25012987 2
SA chrY III cg10213302 3
SA chrY IV cg05672930 3
Non saliva 4
SA(-)_chrY_I cg03905640 4
cg09804407 no HhaI cutting site 4
cg01828798 no cutting site 4
cg04351468 No cutting site HhaI 4
cg14442616 No cutting site HhaI 5
Blood 5
BL_Y_I cg03416979 5
BL_Y_II cg14029254 5
BL_chrY_III cg08265308 5
BL_chrY_IV cg10363397 6
BL_chrY_V cg09460641 6
cg00050873 unspecific 6
cg14005657 not tested 7
cg04016144 not tested 7
cg06322277 No cutting site HhaI 7
cg15935877 not tested 7
non semen 8
noSp_Y_I cg02288797 8
noSp_Y_II cg04964672 8
noSp_Y_III cg07728631 8
noSp_Y_IV cg23834181 9
cg27539833 No cutting site HhaI 10
cg01053349 No cutting site HhaI 10
cg03515901 No cutting site HhaI 10
cg04477336 No cutting site HhaI 10
cg17115812 No cutting site HhaI 10
cg26520468 No cutting site HhaI 11
cg23308414 not tested 11
semen 11
cg00035864 No cutting site HhaI 11
cg00479827 No cutting site HhaI 11
cg04817258 No cutting site HhaI 12
cg25918849 no cutting site 12

cg08921682 No cutting site HhaI 12

cg08593141 unspecific 12

cg05202434 no cutting site 12

cg15781156 No cutting site HhaI 13

GCGC HhaI /GlaI

CCCGGG SmaI

RCGY GlaI

Saliva

SA_chrY_I   cg27355713

GGGGAGGGGGAGTGGAAATCTGTTTAACTGAATTAAGGCCGCGCGCGAAGGGAGGAAGGAGCGCTGGGGACTTGAGTACACCAGGGCCCGTCCGTGTAAG
CACTGGCAACGCAAAGCACCTTACAAACGCCCGAGGTCTGAAGCCTGGGAGTGGCGGTGGAGGTGAGTCTCCCAGCCTCCTCTTCAGTTCCTACACCCCAGCTC
CTGGCTTTTACGCCAGTTTGTCCCTAAGAATTTCTGGCTCTTTTTCTGGGAGGTGTTTTGCAAAGGGGGGAGCCTCTGCCTGTCCCCTTTTTCGAGATGGGAAAT
GACAACTGGGGTGTCTTCTAAGACGATTTTGACGGAGATACACCCTCGGACAGGTGACGGGAGTTCCGTGGGGGCAGAGGGCGGCTTCCTTTTTCGGTTGGG
GGAAGGCAACAGTCTAGCAGTGGCCCAGGTTCCCCTCGCCCACCCTCGGCATAGGGGAGGGGCCGGTTCGCAGTTCCCCACCCTCCTTAGGGGAAGGGCTGG
TTCCGGATCTAAACCACTGGGGCGCTGGAGCTGGGGCTGGTGGGAGGCGGGTTTCCCTCAGCTGGCTGTTGGGGAGGGGCGGGCCGCGGCGAGTGGGTGCC
CTCTCGCTCTCAGTCCTCCCGGTCCGCGTTTGCATGCTAGCGGCCGCTCCCAGTGGTGGTCACCGCCAGTGCCAATCCCTTGCGCCGCCGTGCAGTCCCGCCCTC
TGTCGCTGCAGCCGCCGCGCCCGCTCCAGTGCCCCCAATTCGCGCTCGGGAGTGAGGAGCCGCCAACACCCCAGCTCCGCTGCTGCCGCCACCGCAGTGCTCTC
TAGTCGCCATTGGTTACCTAAACTTTCCAGTACCCGCGACCTTTCCGGGACTCCGCGGCGGCGGGGCAGAGGCGGCCCAGGGGCGGCACGCGGCTGCTCCGGT
GCGCCGGGCTCTTTTCGGCCGCCGCACGGCCGGGCCTCACAGGCTGCCCTCCTGAGAGAGGCTCAGAACACGGCC

cg27355713 (Speichel 63%, Blut 5%, Sperma 3%)
cg04042030 (Speichel 18%, Blut 1%, Sperma 1%)
cg02839557 (Speichel 14%, Blut 1%, Sperma 1%)
cg01707559 (Speichel 16%, Blut 9%, Sperma 1%)
cg15197499 (Speichel 18%, Blut 8%, Sperma 6%)

SA_chrY_I_for: TM: 64°C, 20 nt

AGTCTAGCAGTGGCCCAGGT

SA_chrY_I_rev 60°C

SA_YI_cut1_r, 64°C, 19nt
GAAACCCGCCTCCCACCAG

SA_YI_cut1,2_r, 66°C 19nt
CAGCGACAGAGGGCGGGAC

SA_YI_cut1,2,3_r, 66°C 20nt
GAATTGGGGGCACTGGAGCG

SA_chrY_II   cg25012987

GAGTTACCCGTTCTACCTTAACAATCCCAAGTTGACGGTCAAGTCCAGCGCTCGCCGATGACATCTTATGGACCCAGGGCTCGCGGAAGGACCCAGTTGGAACT
TTATCACACCAAGCCGGCCCACCTTTGCTGCCATCTAACTCCGCCGACCTGCCCGCAGTTCCCCACTCTCCGCTGGTTGACCGGGGCAGCGGAGAGAGGAGGAA
AGCACAAAGGCAGTGACTGGTTCATATCCCATCGCCGCGCGCCTGGGGTGGGGAGAGGGGCCGTGACCCCATCGGCCACCACTGGGCACGCGTGAACACTAC
TCGGACAGGAACCG CAGGGGCACACCCTCGGACAACCACTCCTGAGTCATTCTCACCAGGCTCGGTGAATCCAATCTCAGCCCCTGGAGGAGTTCCCAGCGGG

GTGCGAGGAGGAGGTGGAATCGGAGAGGTGGAAGGAGGAGCAGGGGAGCCACCTCTACCTCAGCAACCCCTGGCGCGGCACCTTGGAAAGCCCGAGGGTG
GGGACGTGAGCCTCCCAATGCACAGCGGGCGCCGATGAGCTCTCGCGAGAGACACCCACTGCGTGATCCGGGGCTCAGGGGTGGGTGG

cg25012987 (Speichel 88%, Blut 36%, Sperma 10%)

cg16292375 (Speichel 21%, Blut 39%, Sperma 6%)

SA_chrY_II_for TM: 64°C, Länge: 21 bp

SA_chrY_II_rev TM: 64°C, Länge: 19 bp

SA_chrY_II_for_alter TM: 64°C, Länge: 19 bp

SA chrY III   cg10213302

ATGCCAAAAGATGAAACACTGCCATAAAGAACACACACTAGACATTACAAATCTTGGTCAAAATACCTACGTTAACGCAAAAAAAAAAATCAATTGGCTCAGTA
ATTGATTAGGATAATCCTCCCCTCCTCTTGCCTGTACACTGTGGCAAGTCCATTTACTTTCTTTTTTTTTTTTTTTAAAGCCAACAAAGGAGACAGTGGGGAATGCT
ATATGTCTGTATCTGCTTTCCTCCTCAACCCTAGGAATAAAGTAAACACGTTTACTGAGGGCGGGGGTCTAAGGGCCTGCAACAATGAGATCTGTCGCCTTGGC
TAGGACTGGCGCCGAGAGGCGATAGGTCTCGGGAGAGCCTGGCGCAGGGTGTGGGAGATTAGGAATCCCAGGTCCACCGGAGATGGCAGGGGGTGGCCTG
GCCCGGTGCGGGGCCGCTTGCCTGCACGCAACCAACTAAGGCGGTGGTGCGCAAGTAGTGGTGACGGCGGGCGCGCGGAGAAAAGGAACGTTGTGACGGA
AACTCCAGCTGCCGGAGACCCCACCGCAGTGAGGTCACTGGACTCCCCGGACTCGGGGCGTGACCGGCGCCGACCCGGGGCGCCGA

cg10213302 Speichel 55% (15%), Blut 10% (3%), Sperma 3%
cg02577797 (Speichel 19% (17%), Blut 8% (2%), Sperma 6% (2%))

cg00272582 (Speichel 23% (22%), Blut 6% (2%), Sperma 5% (2%))

cg24837623 (Speichel 22% (19%), Blut 3% (2%), Sperma 3% (1%))

cg27433982 (Speichel 15% (18%), Blut 3% (1%), Sperma 2% (1%))

cg03683899 (Speichel 12% (16%), Blut 1% (0,4%), Sperma 0,3% (0,3%))

cg02842889 (Speichel 14% (19%), Blut 1% (0,4%), Sperma 1% (0,4%))

SA_chrY_III_for TM:62°C 20 bp
SA_chrY_III_rev TM:60°C , 19 bp

SA chrY IV cg05672930

GAGAGGTAACACCGAGAAGAGGCAGCGGCGGTGGCGCCAAGACGATTGGTGCCAAACAGGGCAGAACGCAACTCAGCTCTGGGGTGGGTTCCTGTCCAGAG
GGGTAGAGAAGAGTGGCTGAGGGCGCGCCCTGATCTCAGCGGGAAGAGTGCGACCCCGAGGGCGCGCTAGGTATGAAAGCCACGTGTGGCTGGCACCGCG
GGGACCCACGCACCAGGTGTACCCTTTCCAGTCCCTGCCGTCGCCTTATTCCCGGTGCTGGTCACCACCGTCTAGGGCTTACTTGGGACACAGGCTAGTTCTCTC
CTGAAGCTATTGAGCAGTATGTGTTGAGGTGCGCTACGCCAGTTGAGGTGAAGCTGTTACACAGTATGAAAGCCGGGCTTTGTAGCTGCAGCTGCGCATTGCA
CCCCCAGCTACGCAGTCTCCTTTCCTTCTCAGTCACAGGACCGGATGGCAAGTGGCCGCAGCCAGTCGGTGAGACCGACTGAGCTCTGGGGCTTCAGTTCTTGA
CGCTACCTACATGGCTACATCTCCAGCCAAGGATGAGAGGTGATGCCAGAGGACCTCGATCTAAATTGGGCACCATTATCGTATGACA

cg05672930 Speichel 36% (15%), Blut 4% (0,4%), Sperma 4% (1%)); cg17660627 (Speichel 23% (16%), Blut 5% (1%), Sperma 4% (1%))
cg07731488 (Speichel 17% (21%), Blut 1% (0,4%), Sperma 1% (0,4%))

SA_chrY_IV_for TM: 60°C, 20 bp

SA_chrY_IV_rev TM: 62°C 20 bp

SA_chrY_IV_alter_for TM: 64°C, 20 bp

SA_chrY_IV_alter_rev TM:62°C, 20 bp

Non saliva

SA(-)_chrY_I cg03905640

AGCTATGAAATATCTGAGATGAGCAAGTGATTCACAAGGAGACAGAAGCAGTATGACCTTTACCCTGTGACCCGGTTCAATTATTTCAGGCTCTCATTAACCAG
GCGAGCTCCTCTTTCTCTCAGAGGTAGGTAAACTTGAGGGGGTAAAGTAGGATTTGGGATTTGGGGATAATGGAAAAGAAAGCCTGGTAATATTTCTTCTATCT
CTGTTTTAAATAAAAAGTAAAACATAAATGCCCTTTCTCAGGGCCCAAATGTTAGGCAAAAACAATGTTTGTCATCTCAGTACACACATGGTCATTTATTTTTTCC
CTCTGCATGTTGTGCGCTTTTTCAGTTTATAATGTCCCTGATCCACTTGTCTCATACTAGCTGCAAACAAGGAAACTGAATGTCACACCAGGTGGCCAGCATTTCT
GTGAAGGGCAGAAACCGGGGCAGCCAAACAGCTGGCAGAGGCCAGCTAGTAAGTACCTGATGCCCACTCCGTAGAGGACTCCACACTTAAAAGACCAAATAA
GCAAGTGTCAGGCTGACTCACTTGTTATCCCCCCAAATAAAAAAATAAAAGTAACCCCTCCAGGGAAAGTGTGTGTGTA

cg03905640 (Speichel 9% (14%), Blut 94% (1%), Sperma 94% (1%)),

SA(-)_chrY_I_for

SA(-)_chrY_I_rev

SA(-)_chrY_I_alter_for

SA(-)_chrY_I_alter_rev

cg09804407 no HhaI cutting site

TGCATCAACACTTTTAGTTATGAAAGAAGTCTTCAAGAGACCAGCACTGAAGCATGTACTTGAAATGCACCATCTTGTACAGTTTTTTTTTTTTTTACAAGAAACT
GAGATTCAGAACAGTGAAGTATGTAGCCTAAATATATATGTGCACTTGAGTAGAACAAGGAAAATTCGTGTCCAAAGTCTACACTCTTTTCATTTGATGATTTTC
CCCTTGTGGCCTGATAAATATCCACATCACAATGACAGGATGGCCTGGATGCATGCTTCTATTTTGCTCCTACTGGAAAACTTTTAGATCTGCATGCATATCCCCT
TAGGAAAGAGTGAAAATTGCCTTAAACATTTGAGAAAAAGTTCTTTTGATAACCCGTCTGGTAAACAATAGTGAAATTGGTAGGTGTCATTATTATCACTTGCAT
AACCTGTACAATTCTTGAACGTCGGTTTGTTCATTCAACATAGATGTGATGAGTGTTTTCTAAATGTCAGGCATTGTTTCTGGTGATAGGATATACAGCCAGGAT
TAAGAAAAGTGATGGACACTAGGCATGGTGACTGACGCGTGTTATCCCAACACTTTGAAAGTTTGATTGAGGAG

cg09804407 (Speichel 9% (14%), Blut 85% (4%), Sperma 88% (2%));

cg08816194 no, saliva 14%, blood 87%, semen 89%

cg01828798 no cutting site

GCCTCCCAATTTACAGTAGATTTATGATCCACATAAAAATAATAAACAAGGAGCCCCACAGCTCAAGCCAAGCCGCATGGACAGGTCATCAACTAAAGGGTTGG
TGACTCAAAAAAAAAAAAAAGGAAGAAGTGATTACACACACACACACACATACACGATGCCACACAGACATGCGGACATTCAACATTAGCAACTCTCTGACAG
AAACACAGCCCAGCAACTCCCTTGGCTGTGTGTTTCTGCAGGAAACCCCACCTCAAGAGAGCCAACCTAAGGTACAGAGGCAGGCTAGAAATCTGACTGTGGC
AAGTTTCAAAAAGACTCATATCTACAACCCCTAGGCAGGCCAAGGAATCCTGCAGATTTTTTGTGTCCTTAATGATTCCACAATTTATTCCTGGGGTTGTATTTGA
CGTTTCTTCAGGCTGGCTCTTGTCTGCCCTCTCTTGGGATCATGGAACTATCCCATCTATCCCAAAGAAGATAGGCAAGAGTTCGCAGCCAATGCAACTCAACGG
AAGTGACCTTCTCCTCCAAGCCACAGAGACTTGGCACCAGGCAACAATAACATCAATTGTGATGCTACCCAGGGCTCACAAACAGTCCTGGTGCCCTGAGACTA
GTGCATGTGCATTCGTGAGGCAGGATCTGGTGCCAGGCTTCAGAGCTTTCAGCCTGCCTAAGCAGTGGGAAATGGT

cg01828798 (Speichel 10% (16%), Blut 66% (5%), Sperma 91% (2%)

cg04351468 No cutting site HhaI

ACAGATTGGGATAATGTTGTTTATTTATTTTATTTATTTTTTATTTTTTTGAGACGGATTCTTGCTCTGTTGCCAGGCTGGAGTGCATCAGCGTGATCTCGGCTCAC
TGCCACCTCTGCCTCCTTGGGTTTAACTGATTCCCCTGCCTCAGCCTACTGAGTAGCTGGAACTATAGGCACACACAACCAGACGCAGCTAATTTTTTTGTGTGTG
TGTTTTAGTAGAGACAGGGTTTCACCATGTTGGCCATTATGGTTTCTTTCTCCTGACCTCGTGATCTGCCTGCCTTGGCCACCCAAAGTGCTGGGATTACATCCGT
GAGCCACCATGCCCGGCATGATTGGGATAATGTTACAAAGCAAAAAAGCACTAAAGAGCACAGAATGGAATGCTCTTGACTACAATGTAAAGGAATTCAATAA
TTAATATAATTACATAAAAGTTTAAAGCTTTAGTAAACACACAATCTCTAGATTTAAGACTCAACAGGACAAGAGACCATTGGTTGAATCAAAACAAGTCCTCAA
ACACACTGGGGAAATGAGTAATTAGCTATTCATGTTACATAAATCACTCTGGTGACAGGAAAGAAATGTCCTTA

cg04351468 (Speichel 9% (14%), Blut 90% (2%), Sperma 92% (1%)); RBMY1A3P

cg14442616 No cutting site HhaI
ACCTAGGCAAACGGCATGCTATCACAAGAAAGGTTTAAAGCTTTGATAAAATGGGGGAGATTTAATCAGTTTTTTTAATGCCTGCTATAAAAATTTGAAATATTA
GAATGGCCGACCATGGCAGTGACCAGGCCTCACTACAGGCCTGGTTGGATTCTGGTCTTTAATGCATGCTAGTGTTGATGTTTTTTGGTCAAGAACGGTTTAAA
CAGGAAGGATTGTGCAGCAGGCTTTAATTTAATGTAGATTCATACTGCTCTGTTAAAGCTGCATTGAAATGTTAAAATGGCTTACACTTGCAGACTTTGCAAATC
TTAAGACTAACAAATCCTTGAAATCACACAGCTTGCAAATACGTACTAAACTGCACAAGGTGTGTGTTCTATATGTGCAGTTTTAGCGTATTTTAGTTGCATAGG
TTTCCATGGTATTTATAGTCTCTTGTGCTAAATTTGGCCAAAGATGATTGTCCACCACTAAAAATGCCTCTCCCACTTGGAATTCTGTACTGATTTTGTGGCCAGA
TGCAATGATCTTTAAAAACAAATCTTTTCAATGGCATAAGAAGTTGACAAAAATTTCTTAAAGTGCAATAGATTTT

cg14442616 (Speichel 7% (11%), Blut 94% (3%), Sperma 92% (2%)); DDX3Y (DBY)

Blood

BL_Y_I cg03416979
ACAATTACAGGCTTTACAGAGGCAAACTGAAACTCAGATTATTTACGGCCCCAGACTTCTACATAAACTAACGTAATACAATTTATGTCAAAATTTGGTAATTCC
CGTCAAGCAAATCAGACATGTGACATGCGCTGACTAAAAGTATAAGTTTTTAATAGCCTTGGTTAAATATATTGCCTGTATTTTGAATGATGACCACATTCACAG
AGAAAACCCTGTTTAATAAAAAGTGCACATGGCGCCTTAGCACCATCTCCCACAACTTGCCCACATGTCGGACATATCCGACAGTTAAAGGTAGAATCCTCAAG
AAAATTAATGAGTTTAACAAAAATGAGTTTCTTAATAGCACTAAGGAGTTCTCTCCCCACTGTCTCCTCCTATAATTCAACACCCTC

cg03416979 **Blood 91%, saliva16% (max 60%), sperm5%)**

Blood_Ychr_I forward: TM: 64°C, Länge: 22 bp

Blood_Ychr_I reverse: TM: 60°C, Länge: 20 bp

Tested by MTA students Primer Blast>> viele matches bei 199☹ nochmal neue Primer

Primerpaar 2
Blood_Y_I for_neu
ACCACATTCACAGAGAAAACCCT
Blood_Y_I rev_neu
CGACATGTGGGCAAGTTGTG

BL_Y_II cg14029254
GTAAGACGTCTGCGTCCTAGCTCCGGATTTATCTGCCTCCATCCTTTGAAGAAGGGCCTTCCATCTTGGCAAGCTTTTACTTTTCGGGTACCGCTGCTTGGCGGG
TGGGAATGCTGCTGGTGCTGCTTCAGGAAACCCGATTTGGAGAGCGGAGGTCTGAGAAGAGTGCGGCTGGGGGTTCCGGGCTGGCCCTTGTGCTCGGCCTGG
GCGCCGGTGGTGCTGTTGGCACGTGAGTCTTGTTAGGAATAAGCTTCCTCGGCTAACCTTACAAGTCCACCCTTGACTCCGCAGCCCAGCACTGGCTTTTTACGC
GGGTTAGCTTATATCCATACGTTGAAGCAGTCTCAGATTCGCTTGGAGAGCACTGTGGACGCTGTCTTGACAGACGCCAGAGAGACTCG

cg14029254 **(Blood 89%, saliva11%, sperm3%)**

Blood Ychr II Forward: TM: 66°C, Länge: 20 bp

Blood Ychr II Reverse: TM: 64°C, Länge: 19 bp

BL_chrY_III cg08265308
GCTCTCGGGGTCTGTGCTGAGGATAACTCAGCTCCGCCCTCGCAAAGGCAGACAGCGCAGGCGCCGACAGCGACAGCTCAGACAGCGCTGGCGCGGCGGACA
GGCGCACAGAAGACCTCAGGCTCAGGTTCCACTCCCCAGCTGTGAAAGGGTAAGAACTGAGGGTGGCTGAGGCTGGGGGTTGTTCAGGGCGGGGTGGGCTC
TGGACCCAGCAGGCCCTGCACCCAGGCCAGGGCTTCAGGGGAGGCCAGGTAAGGCCAAGATGGGGCCGGGGCTGGTCAGAAAGATCCGAGCGTTGGGAGC
CGGTGGAAGAAAAGAGCGCGCGGGCGACAGTCAAACAGGCCTTGGGGCAGGGCACGCCTCGCGCTCCAGGCAGCCCTGCCAGCCCAGGGCACCTGAGCAGC
AACCGCCGCCTGCACTGGGCGCGCCAGAGAGCTGCTACGGTGGTTTCTCCCCCTCGGGCCTGTTGGGCGGGGCCGTTATGGTGCACGTGCTCGCTGGTCCTAA
TGGTTCTCTTGGGCGTTTCTGCTGAGAGGCGGGAGGCGCTGAGAGTCTGTGCGAAGGTCCGTGGACAGACTGCATTGCTTGTTGTGCTTCAGAG

cg08265308 (Speichel 4%, Blut 90%, Sperma 2%)

cg21106100 (Speichel 24%, Blut 82%, Sperma 3%)

BL_chrY_lll_for TM: 62°C, Länge: 19 bp

BL_chrY_lll_rev TM: 62°C, Länge: 20 bp

BL_Y_lll_for_A TM: 64°C, Länge: 21 bp

BL_Y_lll_rev_A TM: 64°C, Länge: 19 bp

BL_Y_lll_rev_B TM: 64°C, Länge: 21 bp + BL_Y_lll_for_A

BL_chrY_lV cg10363397

TCGTGTTCCAGGGTGCGTGTATGCGCGTGTTGGAGGGTGCAGGTGGCTCCGCGCGCCTCATTTCGGAGTCCCTAAAGATTCAAGGTCTTGTACACTAGCGTAG
AGCGTCAGAGAAGTGCCGGGGACAGGGGATTTGGCAGGACGGTGTTTGGATACGCCAGAGACAGGCTCCAAGGTCACTGTCCCCCAAACCCCATACTTAGGT
TCTTTTAGCCGGCGAGTAGAGGAGTGTGTGTGTGCGCTTGGTCGCGTGTGTGGGAGTCTACGGGAACCGCGCGCTTGGCTCCTGAGGGTTGCCCGGACCCTGC
CCCGTGGCATGGACCCCCGCGGGGCCTGCGCGGAGGCTCTGGGTGCGGGTGGGCACACTATGGCAGGTTTCGGACTCAGGGGGTGCAGAGTACAGCTGCTG
CCTCTCCCAGCCCTGTCGAGCACGCGAGTTCTGAAACCCTGCGTATATTTTTCACCTCTCCTGTCATTCTTACCTCTGTCTGTAGGTGCCCACCTGTTCCAAGTTTC
CACCGTCTCGCCTCAGGGAAATTCTGGGGGGACCAAAAGGAGAGGTAGGTGCTTGTTAGGAAGGCTGGCTTCCTGGCTGGGCGGG

cg10363397 (Speichel 12%, Blut 94%, Sperma 1%)

BL_chrY_lV_for TM: 64°C, Länge: 21 bp

BL_chrY_lV_rev TM: 64°C, Länge: 21 bp

BL_chrY_V cg09460641

CTGCCTAACGAAGAGAGATGTTTTGTGTGTTCATGACACATAGAGATGATCATGGCTTGCCACACTCAGGATGTCAGGGCACAGGGCTACCATGCCCACAATTC
CAAAGACCACGCAGCCCGCGTGTGCCTGGACTCTGAGCTACCCGGCACAAGCTCCAAGGGCTTCTCGGAGGAGGCTCGGGGACGGAAGGCGTGGGGTGAGT
GGGCTGGAGATGCAGGCGCGCCCGTGGCTGTGCAGCCCAGGGAGACGCCCGCCGCCCTCCCATTGATTGGCCATGAGGGAAGGAAGTCGGCCTGGGTGCGG
CCCCTTGGCCCTTAGCGCGCAGTCCCTTAGGGGTCTCCTGGAAACCCGGCGCATGCGGCCCTGAGGGCCCGCTGACCCACCGGGTGCCCATCTGCGACAGGGT
TCCTATGGCGTGGGTGGAGCAGCACAGGCCTTGGTGTGTGCGGTGCCGAGGAGGGCACTGCCTTCAGGATGGAGGCTGTACAGGAGGGGCGGCCGGGGTG
GAGAGTGAGCAGGCGGCTTTGGGAGTTGGAGGCAGTGCTGGTGGCAGATGACATAATGGCGGAGGTGGAGGTGGTGGCCCAGGAGGAGGCCC

cg00455876 (Speichel 13%, Blut 79%, Sperma 6%)

cg09460641 (Speichel 14%, Blut 96%, Sperma 5%)

BL_chrY_V_forA TM: 64°C, Länge: 21 bp

BL_chrY_V_revA TM: 64°C, Länge: 21 bp

BL_chrY_V_forB TM: 66°C, Länge: 20 bp

BL_chrY_V_forC TM: 64°C, Länge: 20 bp

cg00050873 unspecific

CTGGTCCCCAGGGGGACCTCAAACTGACTCAGACACTGGACTCCTCCCACAGACCCAGGCTCCCCAGCCTGACCTGCAAATCCATCACGTAGCAAAGCAGGACT
TCCGCATGCTTTCCGACCCACGCCGACATCTCGTGTGCCAAACAATCTACCTCTGCGCAAGAACTCTCCAGAGGATTGGGTGGGCAAGCCTCGTGACGCCTTGC
AATTTCGCAAGAACACAGACAATGTGGAACAGGGCCATCTCCCAGACATTTGGCCAGTCACCCTTCATTGTTGGCCCTCTATCTCTGTCTGGCGAGGAGGCAAC
GCACAACTGTGGTGGTTTTTGGAGTGGGTGGACCCCGGCCAAGACGGCCTGGGCTGACCAGAGACGGGAGGCAGAAAAAGTGGGCAGGTGGTTGCAGCTGA
GGGACGGGAGGGACCGGGGGTGGTGTGAGGCGGCTGCTTCTCTGGGTTTCTGAGATGCAGGAGGCCTTTGTGTGCTGGGTGCTGGACATGCTCCGCTGATGT
CCGGGTGTGTGGTGTCCTCTTATCCTAGTCTCCCTGAGGGGTGGGCCTGTCCACCTGAGGGAAGCCTTGTAGTTAGAAGCCACAGC

cg00050873 Speichel 11% (16%, max: 63%), Blut 89% (1%, min: 88%), Sperma 10% (4%, max: 22%)
cg03443143 Speichel 30% (19%, max: 73%), Blut 92% (2%, min: 90%), Sperma 8% (4%, max: 25%)

sequence not specific

cg14005657 not tested

TCGCCCTGCTGAGAACCTGGTCCCACACCTACGTGGACCCAGGTTTCCTGAGGAGCTCCGCTGGACCCGCAGATCCCGCACTGGCCAAAGGGCTCCGGTCCCCA
GCAGGCTCAACTGCGCACAGGAGCTCGGGAGCCAGAGGCCCCGGCCCTGGGCTTGCAGAGCCCCACCAACAGGCACCGCAACCGCTGCTGCGGGTGCGGGA
GCCTCTGGGTCGTCAAGGCAGCGCACAACAGCGTGCGCGCAGGCCGACAATGGCCAACCCTGGCGGCTGGCCTCTGGTGTGCCCAGGGCATAGGACAAGAG
GCCCTTTGGAATGCTCCTTGGAGTACAGCATCCTCAGGGAGGAAGCATGGTACTCGGAGCCTCTATTTGCCTCGACCTGTGAGAGTGTGTGCCGGGGCTCTGG
CCTCTACAGCAGATCAATTCCACCTCAGCACCGGCAGGCGACTTTCCTCCCACGTGCCCGCCCCGATCACTTCCCCCAGGACACCCCTGCCGCCCTTGCCCCAGC
AACCAGAGAGAGTTCTCTGCATCTGCTGTATTACCTCCGTACCATCTACCTGGCCTGCCTAACGAAGAGAGATGTTTCCTGTGTTCA

cg14005657 Speichel 12% (16%, max: 56%), Blut 75% (1%, min: 74%), Sperma 5% (1%, max: 9%)
cg09350919 Speichel 19% (19%, max: 73%), Blut 97% (0,3%, min: 94%), Sperma 11% (2%, max: 22%)

cg04016144 not tested

CTGCGCAGCCCAGCCCAGCCCAGCCCATGCCCCGCACCCGTAGGCTCTGGGGGCCCGCTCCCCAGCAGACCCGCTCCCTGCAAGACCCACGGGCGTCGCCCTG
CTGTGAACCTGGTCCCACACCTACGTGGACCCAGGTTTCCTGAGGAGCTCCGCTGGACCCGCAGATCCCGCACTGGCCAAAGGGCTCCGGTCCCCAGCAGGCT
CAACTGCGCACAGGAGCTCGGGAGCCAGAGGCCCCGGCCCTGGGCTTGCAGAGCCCCACCAACAGGCACCGCAACCGCTGCTGCGGGTGCGGGAGCCTCTGG
GTCGTCAAGGCAGCGCACAACAGCGTGTGTGCAGGCCGACAATGGCCAACCCTGGCGGCTGGCCTCTGGTGTGCCCAGGGCATAGGACAAGAGGCCCTTTGG
AATGCTCCTTGGAGTACAGCATCCTCAGGGAGGAAGCATGGTACTCGGAGCCTCTATTTGCCTCGACCTGTGAGAGTGTGTGCCGGGGCTCTGGCCTCTACAG
CAGATCAATTCCACCTCAGCACCGGCAGGCGACTTTCCTCCCACGTGCCCGCCCCGATCACTTCCCCCAGGACACCCCTGCCGCCCTA

cg04016144 Speichel 14% (19%, max: 90%), Blut 95% (1%, min: 93%), Sperma 9% (9%, max: 42%), TSPY4

cg06322277 No cutting site HhaI

TGGCCCTAATTCAGAAAAACGAATTGTTTCTTAAATCATGATTTTACAATACAACATGTCACAGTCACAAGGAATCATACAGGTTATTTCTTAACGATTGGCTTTT
TTTTTTTTAAGGCGGCTAAATCCTGCTCCTTCAGAGCTTCTGTTAAATATTTTTAAAGTTGGCGAAAGATAATTTTTAGAGTGTAAACTGCGTCCTCCCAGCTTAG
CCGACCTGAGGGGAGGAAGCACAACAAAAAGTCCCGTGGCATAACTGACACCTATGAATCAGTATCAGAAACTGAATATGCTAAGAAACTCAGTTCCGAGCTG
TGACCTCTACAACGGAAGAATAATAGGTTGGTTACCCGAGGCGGAATCTGGAACTGAAATTCTCACTCATTTGACTTCCCGCTGTTTATCACCGCAGATTACGGT
TGCACCGTAAAAGGAGAGGTTCTGTTCCGTCGCGGGATTTCGCCATGGTAAGACGTCTGCGTCCTAGCTCCGGATTTATCTGCCTCCATCCTTTGAAGAAGGGC
CTTCCATCTTGGCAAGCTTTTACTTTTCGGGTACCGCTGCTTGGCGGGTGGGAATGCTGCTGGTGCTGCTTCAGGAA

cg06322277 Speichel 14% (15%), Blut 96% (1%), Sperma 1% (1%), RPS4Y2

cg15935877 not tested

TCTGCCACCCCACGTCGGGCTCCACCTCAACCACCACCTCCACCTCAGCCATGATGTCTTCCACCTTCAGCACCGCCTCCTCTTCCAAGGCCGCCTCCTTGCTCTGT
ACCCCGGCCGTCCTCTCCAGCATTGCCTCCAGCCTGAACACGGTTTTCTCCTGGGTGCTCCCACAGACCCTGGGCCTGCGCAGCCCAGCCCAGCCCAGCCCATGC
CCCGCACCCGTAGGCTCTGGGGGCCCGCTCCCCAGCAGACCCGCTCCCTGCAAGACCCACGCGTCGCCCTGCTGTGAACCTGGTCCCACACCTACGTGGACCCA
GGTTTCCTGAGGACGTCCGCTGGACCCGCAGATCCCGCACTGGCCAAAGGGCTCCGGTCCCCAGCAGGCTCAACTGCGCACAGGAGCTCGGGAGCCAGAGGC
CCCGGCCCTGGGCTTGCAGAGCCCCACCAACAGGCACCGCAACCGCTGCTGCGGGTGCGGGAGCCTCTGGGTCGTCAAGGCAGCGCACAACAGCGTGCGCGC
AGGCCGACAATGGCCAACCTGGCGGCTGGCCTCTGGTGTGCCCAGGGCATAGGACAAGAGGCCCTTTGGAATGCTCCTTGG

cg15935877 Speichel 15% (12%, max: 60%), Blut 92% (1%, min: 90%), Sperma 5% (4%, max: 25%), TSPY1 (TSPY4)
cg05544622 Speichel 16% (18%, max: 52%), Blut 98% (0,3%, min: 98%), Sperma 4% (6%, max: 30%)

non semen

noSp_Y_I cg02288797

TCTGAACTTTAAGAACTCAAGGCAGCGTCTTCCCAGAGCTTTAGTTAGGGCCGTTTCAGAACAGACTTCTTCCCATGGGCCTTCCAGGCAGTGACAGGCTCAGA
CACTGCTATTGGGAATAGCCAATGCTATTCCGAAGAAACAAGCCCATTGAGTTTCAGGACTATATTTTACAAAAGGAAACTGGTGAGAACCCAAGGTCCCCTTC
ACTTGCTGTAAATCACCCCCTCCCAGCAGCTGAGGCTGGTATCTCCATTCCCTCCAGCCCCAGAGAGACCTGGGCGAGGGAGTGAGGGGGAGACTTGGGGTTT
GGGGCAATCCTGCGGTCAAAGTGGGGTTCTCCCCAGTGCAGACCAATCTCTCACCCATGCCAGTAGGAACAGGCAGGCTGAACTGAAGGAGCTTGAGCTACTC
ACCCGACATGGTCCGTGGCGCCGCACGCAGTGGGCGCGGACCCGCAGACCTGCGCCCAGGCGCAGTGGGTGGAAATAAATCCTCGTCACGAACCCGCAGAAC
AGAGGCGCCGTCTTTGGGAAGCCGCGTGGATTTCCAGGCCTGGGGGCGACAGCGTGGCTGCCTCGACACCCTCGGCTGCAGGCCT

cg02288797 (Speichel 87%, Blut 87%, Sperma 9%)

noSp_Y_I_forA TM: 64°C, Länge: 20 bp

noSp_Y_I_revA TM: 66°C, Länge: 22 bp

noSp_Y_I_forB TM: 64°C, Länge: 22 bp

noSp_Y_I_revB TM: 64 °C, Länge: 19 bp

noSp_Y_II cg04964672

TACATGCTCACAGAGACATACAGGGGCATATACACAGACATATACACATGCATACACACACCAAACCACACAGATACACACAGAACAGACACCGAGGCACACA
GACACCCACACCCAGCCACTCACCATCTTTGCTAAGGGGAACAGTACTTTCAGAGTCTTCAGACCTACTTACAGCCGCATCCCTCCTTACATGAACTGGATCCCA
CATGCCGCCGATACACCCAGCCCTGTGACCATCAGAGGCCCTGTCTCCCCTTTCTTGAGGGAGCCACCCCCTACCTGGCTCTCAAACTCATTCTCTACCTGGAAA
ACATAAAGCTCTTCTATGACCGTCCACACCATCGTCCAGCAAGCCTACTGGACACACAACCGCACTTTAGCGTCCAGCTCCTTCCGGAACCTCCCTAAAGGAATC
TAAACAAGGACCACTCCAAGCCGCAGCGTGGAAAGCCGCCGTGGTGCTACCAGGCGCTACCGTGGTCCGTCTACCATGTGGCGTCCACAGTCCGTTGAGACTC
CGGCAGGAAGCCTCGTCCCTGGCAGTAGACAGAGCCTTTTCCCCAAGCTTTCCTGGCTGCCGACATCCCGCTGCTTGGTGTCCACCGCCCTCTGCTTGGGAGGG
AGATGAGAGCCCGGCCAGCGAGCTACCGCACATGGCTCCTGGGTCCCGCACGCTGGCCGGGAAAGGAAGCACCTTA

cg04964672Speichel 62% (8%, min: 54%), Blut 84% (12%, min: 60%), Sperma 3% (5%, max: 25%)

Sp (-)_chrY_II_for TM: 66°C, Länge: 20 bp
Sp (-)_chrY_II_rev TM: 64°C, Länge: 20 bp
Produktlänge: 71 bp, 1 Schnitstelle, keine Nebenprodukte

Sp-_Y_II_lalu_r
PCR Produkt: 153bp

noSp_Y_III cg07728631

CACTACCAGGACGTCACCGTCTACCTGGGAGACACCATTACAATGGAGTGTCTGGCGAAAGGGACCCCAGCCCCCCAAATTTCCTGGATCTTCCGTGACAGGA
GGGTGTGGCAAACTCTGTCCTCCGTGGAGGGCCGGATCACCCTGCACCAAAACCGGACCCTTTCCATCAAGGAGGCGTCCTTCTCAGACAGAGGCGTCTATAA
GTGCGTGGCCAGCAACGCAACCCGGGCGGACAGCGTGTCCATCCGCCTACACGTGGCGGCACTGCCCCCCATTATCCACCAGGAGAAGCTGTAGAACATCTCG
CTGCCCCCGGGGCTCAGCATTCACATTCACTGCACTGCCAAAGCTGCGCCCCTGCCCAGCGTGCTCTGGGTGCTCGGGGATGGTACCCAAATCCGCCCCTCGCA
TTTCCTCCACCGGAACTTGTTTGTTTTCCCCAACGGGACGCTCTACATCTGCAACCTCGCGCCCAAGGACAGCGGGCGCTATGAGTGCGTGGCCGCCAACCTGA
TCGGCTCCGCGCGCAGTACGGTGCAGCTGAACGTGCAGCGCGCAGCAGCGAACGTGCAGCGCGCAGCAGCGAACGTGCAGCGCG

cg07728631 (Speichel 90%, Blut 84%, Sperma 6%)
cg05213048 (Speichel 65%, Blut 71%, Sperma 7%)

noSp_YIII_forA TM: 64°C, Länge: 19 bp

noSp_YIII_revA TM: 64°C, Länge: 20 bp

noSp_YIII_forB TM: 66°C, Länge: 20 bp

noSp_YIII_revB TM: 64°C, Länge: 19 bp

Sp-YIII_la_f TM 64°C,

Sp-YIII_la_r (reverse primer not Y spezific), TM: 64°C

CAAATTTCCTGGATCTTCCGTGACAGGAGGGTGTGGCAAACTCTGTCCTCCGTGGAGGGCCGGATCACCCTGCACCAAAACCGGACCCTTTCCATCAAGGAGGC
GTCCTTCTCAGACAGAGGCGTCTATAAGTGCGTGGCCAGCAACGCAACCCGGGCGGACAGCGTGTCCATCCGCCTACACGTGGCGGCACTGCCCCCCATTATCC
ACCAGGAGAAGCTGTAGAACATCTCGCTGCCCCCGGGGCTCAGCATTCACATTCACTGCACTGCCAAAGCTGCGCCCCTGCCCAGCGTGCTC

Sp (-)_chrY_lll_forA TM: 64°C, 19 nt

Sp-YIII_Glu_rI TM: 66°C, 22nt

Sp-YIII_Glu_rII TM: 64°C, 20nt

Sp-YIII_Glu_rIII Tm 64°C, 22nt

Sp-YIII_sma_for TM 64°C, 18nt

SmaI / GlaI Digestion

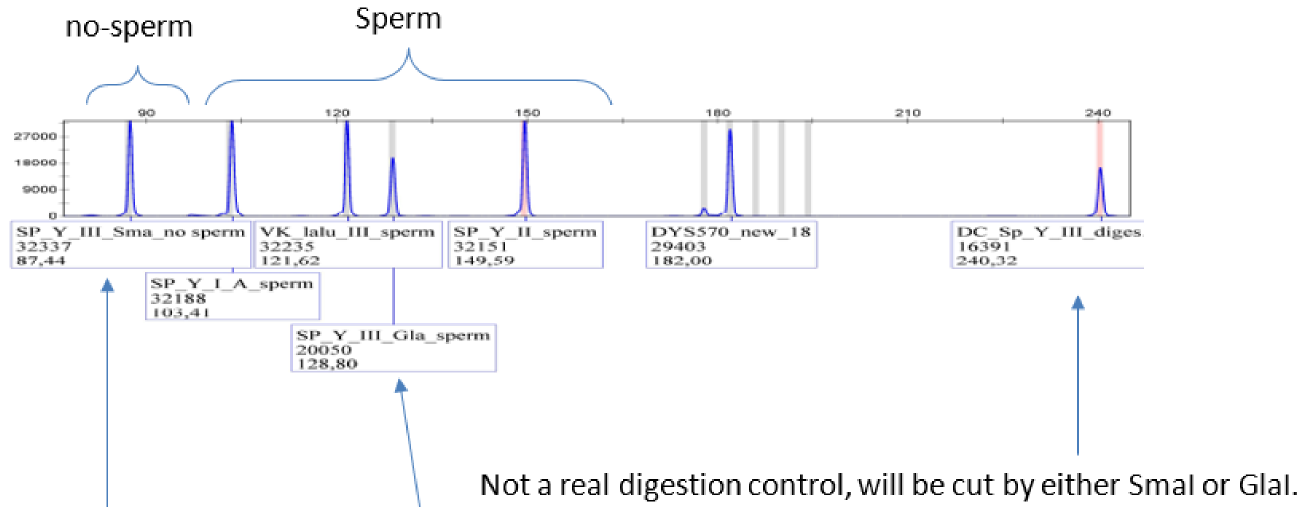

TGGCGGCACTGCCCCCCATTATCCACCAGGAGAAGCTGTAGAACATCTCGCTGCCCCCGGGGCTCAGCATTCA
CATTCACTGCACTGCCAAAGCTGCGCCCCTGCCCAGCGTGCTCGGGTGCTCGGGGATGGTACCCAAATCCGCC
CCTCGCATTTCCTCCACCGGAACTTGTTTGTTTTCCCCAACGGGACGCTCTACATCTGCAACCTCGCGCCCAAGG
ACAGCGGGCGCTATGAGTG

noSp_Y_lV cg23834181

AGGTCGACTAGGGAGATTGCCCCCTAATGGTATTTACCTTAGACCTCGGTGCTTGAGCTGTAATCATTCCTAGAACTACTCTCTTAACCATGTCAATTATCCACAA
GTGTGTTTACTCAGTTTGTGTTGTTAATTGTATACGAAATAAATGCCTGGAGTGCCAGCTGCTCAGGGCCGCCGCAGTGACAAACCTCTCTTGGTGTGCAGGCG
GTCGGACACTCAGCTGGACAGGCAAAGCAGAATACCTTTCTGTCAGTGTACGTTTCATTCATACATCGTTTGGGTCAGGGTCTCCGGCCAGATACCCGCAGCTA
ATGCCCTCAAGTGTGAGGTGCAACACCTCAAGGCTGGGCACCGCTCAAGGTCGCAAGCCTGGGTAAGAGAAGAGAGGACTGAGGTCCGGCCCCACCGCGCTC
TGTGTAGAGGACGCAAAGCCCAGCGCGGGCCCGGTGGTCCCAAGGCACCGCGCCTACGTGCCCAGGTCCCACACATAGGGCGACCGGAATCAGGTGGTGGG
GCCGCACAGACTCCGCGACCACTCGCCACCATCGGCCCAGCCCGCTGGCGTTGCTGGACGCAGCTCCACTTTGGGGCCGGCTCC

cg23834181 (Speichel 68%, Blut 71%, Sperma 7%)
cg25667057 (Speichel 23%, Blut 83%, Sperma 8%)

noSp_Y_lV_for TM: 64°C, Länge: 21 bp

noSp_Y_lV_rev TM: 64°C; Länge: 20 bp

cg27539833 No cutting site HhaI

ACCCCATCATTGGCCACAAAACGGGCAAAGATGCCCATGCCACCCACCTGCAGAGCACCGAGGCGTCTGTGCCGGCCAAGTCAGGGCCAGTGGTCCAGACAAT
GTACAAAGCCTGCCCCTGTGGCCCTTGCCAAAGGGCCAGATGTCAAGAGAGAAGCAACCAGATCCGGAGTCGGCCCCTAGCCAGAAGCTCACCGGAATAGCC
CAGCCAAAGAAAAAGGTCAAGATTTCTATCACTTCCCCGTCTTCTCCAGAACTTGTGTCTCCAGAGGAGTACGCCGACTTCAGCAGCTTCCTGCTGCTAGTCGAT
GCAGCAGTACAAACGGCTGCCGAGCTGGAGCTAGAGAAGAAGCAAGAGCCTAATCCATGATTGATGATGTTCCAAAAACCCAAGTAGTCAGTCCCTTGTGTAC
TGTGGTAAACCTGTTTATGTTCACCCCAACTTATTTGTATGCTTATCTTTTATAGAGGCATCTTTCTTTCTACTGGTTTTATGAGTACCAATCTTAATTATTGGGACT
AAATTCTGTCAGATATTTCAGTGTTTCCAGGTGAAAGACACTAAGGTGCCATCATAATGAACACTGCAGCAAAGATTTCTTAACTCTCCTTCCTCCTGGATTTGAA
CTCTTTAGACCATCAGACTAGTACATCTGAGATTAAACATCAAGCTGAGATTTCAGAGATAAATGTTTTTGGCT

cg27539833 no, saliva 92%, blood 93%, semen 6%

cg01053349 No cutting site HhaI

AGTGAGCCGAGATGCCGCCATTGTGCCACTGCACTCCAGCCTGGGCGACAGAGCGAGACTCCATATCAAAAAAAAAAAAAAAAAAAAAAAAAAAGACAAAGACA
AAGATTTTAAGGCCATAGAGAAGAAAAATTTAAGCAGCAAGGCATCAAAAGCAAAAGTCTAGTTCAAAATTATTATTAATATCTGCTTTGAACATGGAAGAATG
GAAGAGGGGAGTCAACGTACAGTAGTTACACTCTTTTATTGAGTTTAGTAGCTAATCAACAATTTGTTCCTGATATATCTAGATGAGCCAGTGAGACAATTATAA
GTAATAACGGGAAGAACAATACCAGAGTAAAATCAAATAGGAAGAAAGTGATAGGCGTATTTACACTAATAGAAATAGAGTCCGTCAGTTAGTGGAAACTGG
TGACAACAGCAGAGAAAACTCTGGAGAGCTATAAACTGCGTGAAATGAAGTGCATTGGAAGTTACAGAAAATACGCAAGAACAATGGGAGAAGAACTCCAGG
AAGTGTAGGGGAGGGGCAGCGTGGAGAAGAAAGGATGTGACGTCACTGAGATAACCAGCTCGGCTGTTAGCAACTCTGTTAGCAAA

cg01053349 no, saliva 84%, blood 87%, semen 5%

cg03515901 No cutting site HhaI

GACAATTATAAGTAATAACGGGAAGAACAATACCAGAGTAAAATCAAATAGGAAGAAAGTGATAGGCGTATTTACACTAATAGAAATAGAGTCCGTCAGTTAG
TGGAAACTGGTGACAACAGCAGAGAAAACTCTGGAGAGCTATAAACTGCGTGAAATGAAGTGCATTGGAAGTTACAGAAAATACGCAAGAACAATGGGAGA
AGAACTCCAGGAAGTGTAGGGGAGGGGCAGCGTGGAGAAGAAAGGATGTGACGTCACTGAGATAACCAGCTCGGCTGTTAGCAACTCTGTTAGCAAAGCTG
TTTGTCTTTCTCGGAAACAACAGGTGAGAATTCCCCTTACAGACCTGCCCATGCTTTCTAAAGTGGCTCTCCCAAACCTACCTTTGTCCTAACTCAGTTGTCTGTG
ATTCTCAATATAGTAACGATAAGCCTCTTTGAATATGGAGGCCGCTGCAGACGGCCCGGCTGAGACCCAAAGCCCGGTGGAAAAAGACAGCCCGGCGAAGAC
CCAAAGCCCAGCCCAAGACACCTCAATCATGTCGAGAAATAACGCAGATACAGGCAGAGTTCTTGCCTTACCAGAGCACAAGAAGAAG

cg03515901 no, saliva 89%,blood 90%, semen 4%

cg04477336 No cutting site HhaI

CTGTCAGAGAAGACCAATTTGTCTTTGTTGCGGATTTCTAACTGGTTTATCAATGCTCGCAGACGCATTCTCCCGGATATGCTTCAACAGCGTAGAAACGACCCC
ATCATTGGCCACAAAACGGGCAAAGATGCCCATGCCACCCACCTGCAGAGCACCGAGGCGTCTGTGCCGGCCAAGTCAGGGCCAGTGGTCCAGACAATGTAC
AAAGCCTGCCCCTGTGGCCCTTGCCAAAGGGCCAGATGTCAAGAGAGAAGCAACCAGATCCGGAGTCGGCCCCTAGCCAGAAGCTCACCGGAATAGCCCAGC
CAAAGAAAAAGGTCAAGATTTCTATCACTTCCCCGTCTTCTCCAGAACTTGTGTCTCCAGAGGAGTACGCCGACTTCAGCAGCTTCCTGCTGCTAGTCGATGCAG
CAGTACAAACGGCTGCCGAGCTGGAGCTAGAGAAGAAGCAAGAGCCTAATCCATGATTGATGATGTTCCAAAAACCCAAGTAGTCAGTCCCTTGTGTACTGTG
GTAAACCTGTTTATGTTCACCCCAACTTATTTGTATGCTTATCTTTTATAGAGGCATCTTTCTTTCTACTGGTTTTATGAGTAC

cg04477336 no, saliva 92%, blood 91%, semen 5%

cg17115812 No cutting site HhaI

CTGCAGGAAAGGCACTATCGTCCGTGGGAGGACTCCAGCCTTACCACTTTTTTCCTCGGGCTGCCTCAATGTCCTCCTTCACGAAGACTGACATGCTCTCCCCTTC
CTTCCGGGGCTTTCTTGACGTTTTGGGTTATCCCCGTGGGCGAGAAACGCACCCTGAGTGCACACCAGGTATCCACGGTCCCTCGGTTCCGGCGCAGAGGCTGC
TGGGAAGACACTGTCGTCCGTGGGAGGATCCCTGCCCACCGCTTTTTCCCTGTGCTGCCTCAGAAGTCTTCTTTCAAGCCTGGCATCTTGTTCCCTTCCTTTAGGG
GCTTTCGTGTTGCTTTGAGATGCCCCCTTGGGCGTGACCCGCAGCTGGGTGCAAGGCAGAAGCTCCACCACTCGCCTGGCCCGGAGCAGGGTCTGCCAGGAAG
GCACTGTTGCCATGGGAAGTTTTTCCTTGTACTGTCTCAACGTCCCCTTTCAAGCCTGGAGCTCTGTTCCCGTCCCCCGGGGGTCTTCTTGCCGCTTTGAAGTGCC
CCCCATAGGCCTGACACGGACCCTGGGTGCAAGACAGGGACTCCACGACCACCCCGGCCCCAACACAGGGGCTGCC

cg17115812 Speichel 74% (10%, min: 48%), Blut 78% (8%, min: 58%), Sperma 6% (2%, max: 11%)
cg14151065 Speichel 13% (20%, min: 0,3%), Blut 86% (3%, min: 80%), Sperma 5% (2%, max: 9%)

cg26520468 No cutting site HhaI

CACCCCCGCCTGGGAGACAGAGCAAAACCCTATCTCTAACAAATGAAATAAACATAAAAAAACTTTTAAAAACACTAAAACATTCTACCTTCTACACTTGCTTTT
GCTGTCTGGGTGATAAAGTGGAGACTTAGATTAACTAATATTGCTCATTTGAGCATTATCAAGAAGGCACAGTTTATGCCAAAGCTTACTATGTTAATTTTCTCC
AGGAGTTCTTATGACTCCGAATACCAGGATACAACGGTTTGAGGTTCTCAAGAACGGTACCTTAGTGATAAGGAAGTTTCAAGTGCAAGATCGAGGCCAGTAT
ATGTGCACCGCCAGCAACCTGTACGGCCTGGACAGGATGGTGGTCTTTCTCTGGGTCACCGTGCAGCAACCTCAAATCCTAGCCTCCCACTACCAGGACGTCAC
CGTCTACCTGGGAGACACCATTACAATGGAGTGTCTGGCGAAAGGGACCCCAGCCCCCCAAATTTCCTGGATCTTCCGTGACAGGAGGGTGTGGCAAACTCTG
TCCTCCGTGGAGGGCCGGATCACCCTGCACCAAAACCGGACCCTTTCCATCAAGGAGGCGTCCTTCTCAGACAGAGGCGTC

cg26520468 no, saliva 96%, blood 96%, semen 9%

cg23308414 not tested

GCCAGAGATCAGGTGTCTCAGGGCGGTAAGGCCTTAGACACCCGCAGTGATACCTGGCTGACACAAGAGCCTGGCCCACCATGCCCGCCAGACTCCAGGCATT
ACCGCCACTCCAAAGCCACGCGGCTTGGATCGGGACAACTCTAAGCCACTCCTCGCCTGCTGCTCGGCTGCTCTCCCCCTCCACGCCGGAGCACTGTGGGCCAC
CGCGCTGCACCTTCTGCCGGCTTCCAAGGGCTCCAGCCATAGCAGACAAGGCCATGCACTGGACCATCTCGGCGCCGCCCTGCTGCTGAGTGAGGACGCCAGA
GGTGTCCATCCGCTGCACAGACTTCGGGCTCTCTGGTTGGTCTCCATTCCTCCGACGCTTCAGGGCTTCCCCAGGCTCATGAACTCCCAAGCTTCCACCACATCG
GGCTTGCTGAGCTTGTGCTCCCAGGAGTCAGGGATACCCTTCTGTCCTCCTTGCCACGGAGGGATTGTTTTGGTTCCCTCGCCGCCCCTCCTGCAAGGCCCCCTC
TCTCCTCACCCACCCAGTGCTGCCAGGGCTGCTCAAGGGTGAACAGGTGGCCTAGCCCCATGGGCCCTTTCTCTTACAACG

cg23308414 Speichel 77% (7%, min: 58%), Blut 75% (6%, min: 65%), Sperma 11% (2%, max: 18%)
cg10252249 Speichel 36% (22%, min: 0,2%), Blut 98% (1%, min: 97%), Sperma 5% (2%, max: 14%)

semen

cg00035864 No cutting site HhaI

AAAGTTGGAGTATTCCCACCTGAACACTGGGCCATGGTGTGGACTGCTTGTGCAACTAAGAGAATGTGGGGATGCAGTTTAAAACACCTCTGTTTCACTGTCTT
AACCTTTTTTGCTGGTGAAGGTACAGGGCCCCATCAACCCCTCACCAAATTATATTTTCACCTGTTTGACCTTATTGCAGCTCACAATCTCTTTCCCAGTTTGAAAT
CCAAAGATGATGGAGGAGTGCCCGCTCATGATGTGAAGTACCTGCTCAGCTGGAAACCGAATTTGAGATAAATTCAAGGGTCTATGTGGACAAGACTGCTAGT
GTCTCTCTCTGGATTGGTCATATTGCAATGAAACACCAGACGATGTCTGTTCCTGGGTGTTGTATTCCACTCTTCTTTCTAGAAGAGTGCCTTTATTTGCAGGGGA
AGATAATGGAGACCCTGGCTGGTCACAACTAGCCTACCTGTTCATTGTGGATTCATGACCCACAGAAACATAAAGAACAGGGAGCCCACAGAGCCCACAGCCC
AAGTACAGCCACACAGACAGGCCACCCAAAGGTTAAGACGCTAAAAAATAAAAAGCCCTGCAGTGTGTTAGCCACATT

cg00035864 Speichel 14% (19%), Blut 24% (2%), Sperma 77% (4%)

cg00479827 No cutting site HhaI

TGTAAAGTAATGGAGATTATTCTATGTGATCTTAGTGGGTCTGACTCTGTTAGCAAAAGGCATTAAGAATAGAGGTGCAGCTTCCCTGGCTGGAGAAGATTCTG
CTCATGGACAGGAGCTTCCACTCACTTCTGAGAATTCCAGCTAGCGTTTCTTGCCTTATAGATTTCAGGAATGCCTCATCAGCTCCATGATCACATAGCCAATTCC
TATGTCTTCTTTTCTATCTCCCATTGCCCTTGCTGGATTACCTGAACTGACACATTCACGTTTTCGTAAAATTTACTGCGAAAGTCTTACCGTCTTGCAGCTTCCTTG
CAAAACTGTAACTTCCCCAAAACCACAACCTTGCAGCTCCATTTTTTTAAAGTAAATGTTCCTTTTTCATACTTTGTTCTTGTGGGAGGATACATTGAGATGTGAA
ACATTGTGTTTCAAAATGAATGGGGGATACGATTTGACTATGTCCCCAGCCAAAACTCATTTTGAATTGTAGCTGCCATAATCCCTACCTGTTGTAGGAGGGACC
CAGTAGTATATAACTAAATCATGGGGGAGATTTCCCATTCTTGTGGTAGTGAATAAGTCACAAAATATCTGA
cg00479827 Speichel 12% (17%), Blut 33% (7%), Sperma 86% (4%)

cg04817258 No cutting site HhaI

AACATTCTCAAGGATGTGTGACTCTGTGTCTGTGTGTGTGTGTGTGTCTTTGTGTTTGTGTGTGTGTGTGTGTGTGTGTATGTTTATCCACTTTATTCGGGTGT
CATAATGAATTGATCAATCCACGTGCTTTATTCTCTTCATGGAAATAACCAGTCTGCGTTGGAGCTGGGCCTCTAAAGTTGTAGAGTGAATGGGTGTGGGATGT
GTTGGGATTCTTCCTACAGGACAGAGTGGGAGAGGTAAAAGCAAAAGACAGCTTAGTTGGAGGCTGACTTCGTCCTATGGAAGCAGAGATAGTTCAAGGAAT
GGGGTTACTGGGTTTCCAGGGCCCAGTTTGCTGGGACCTCCAAAATCCTTCATTTTGGGTATCATCATACACAGTAGCTAAGCACAGGATGATGGAAATCTTAA
AGTTCGCTTTCGTGTTGAATCCACATGTTCTTTTAAAGGTGAATGCATGATCCTTTTCTGGGACAATCAGCCTCTCAGGACTTCTAAAACATCAACGTGAGAAGA
AATGGGCATGTAAGGTGTATGGAGGGACTGTGGGAAAGGTGACAGAGGCATGTGGGAAGGCATTCAGGATACGCTTTTGG

cg04817258 Speichel 16% (21%), Blut 36% (1%), Sperma 74% (2%)

cg25918849 no cutting site

AGCCCAGACATCTTTGCTAAAAAATGCCTCTGGGGTCTCAGGTATGATGCTATGACCCAAAGAACCATCAACAACACACCAGACAGACTATATGCCAATCACCA
TGGGACCCGATTCTTGCAGACACACATTCTCTTTTGGGAATGGATTTCAAAGGGCAGTTTTCAGTGACCACCTCAGAGTCTTGAAACACCTCATCCTCCATCAGG
ACACAACCACAGAGATGGTCCAAATGAGCCCTGAGGTCGAGGCTTTTATTGTCCTGCCATGGGTCTTCACAGGCAGCCTTTTTCTTGATACCAGGCCAGCTCCG
ACTGTACCATTTTCCTCTGCTTAGGCAGGCTGAATGCTGTTACAGCAGGACACATAAGCCTGTCTCAGGAATCCACATAAGCTAGTCTCAGGGCACCAGTCCTGA
GTGTGAACTCTGTCTAGAGTCACAGTGAATGTCACTGTTGCCTAGCGACAAGTCCCCGCGGCTTTGTGGAGAAGACTCCCAAGGAGGAGACCTCCATGGAGGT
TCGTCGGCTGAGGTCTCTCACCTGTCTACTCTGTGAGATCCACAGGATAGTCCCATAATCCTAGGATAGGGAGGATGTGA

cg25918849 Speichel 17% (14%), Blut 31% (3%), Sperma 80% (2%)

cg08921682 No cutting site HhaI

AATAACTTGTCTAACATACTCTTTCTGCCTGTGTTGGGCACTGCGGCTTAGAAAGTGAAGGCTTATAATTGTTTCCTTGAGATGTGTGTGGTTTCAACACATGTCC
TCCTTTCAAAGTCATCCATAACAGACCTGGGTAATTGTGTTTTATTGACTCTCAGTCTTAAACTGGTTGGTAAGATTATGAGGAATGTTCACATTAGGGGCGTTC
ACCATTGAGGTTCTTAGGCAGGAAGATTCCACACCCAGAAGGGACTAGGCAGTTGTTCTCTGAAGTAGAGGTGGGAAAATCTGTTGTAAATCGAGAAGGACTG
AGAGCAGGCTCAGCAAACGTGGTCTGTGATGGACCTGATAGTAAGGGGCTTAGGATTTGTGGGCCAGGCAGTTGCAGCTGCTTTACTCTGCCATTGCATTATG
AAGGCCCTACGGAGATGAATGAACATGGCTCAGTGCCAAGAAAACTTTATTTATAGACATTGATACTGGGTTGCAGTTTGCTGACTCTGACCTAGAGCAACACA
CTGTTTTTTTTTTCCTTTTATGTCTTTTTATTTTTATTTTAAGTACTTGGGTACATATGCAGGATGTGCAGATTTGTTAC

cg08921682 Speichel 15% (20%), Blut 53% (5%), Sperma 93% (1%)

cg08593141 unspecific

TCCAGAAGCTGAGGAAGTGGAGGTTGCAGTAAGCTGAGATTGCGCCACTTCTCTACTCCAGCCTGGGTGAAAGAGCGAAACTCTGTCTCAAAAAAAAAAAAAA
AAAAAAAAAAAAAAAAAAAAAAAAAAAAAAGAAGGAAGATGGGCCGGATGCAGTGGCTCAAGGACTGTAATCCCTGTACTTGAGGCAGGCGGATCATTTGAG
GTCAGGAGTTGGAGACCAGTGTGGCCAACATAGTGGAACCCCATGTCCACCAACTCATGACACCGCATGTGGTGTTTATTAATCTACTTGTAAAGGCTGGGCTA
CAAGAAGTCTTAGGAAGACTCTTTTTCTACAGGGTCACCCCACACCTCCAACTCCTGCAAATGAGCCCATCCAGAAAGCTGTCTCTGGAGCACATGCCTGTTTCC
ATCTTCTCTTGCACAGTTTCAGGCCATGTTTAAGGATGGGAGAAGATTGCCTGGCCTCCAGCCTGGATGCCAGCCAGGTATGTGGACTTTCAAGTATGAAAGCT
GTCAGGCTTTGCCTCAGGAGAGCACAACTTATGCACTTGTGGTATAGGCAGTGACTCAGATTCCCTGGGGCTGTGACCTGCTGT
cg08593141 Speichel 25% (21%, max: 65%), Blut 56% (4%, max: 62%), Sperma 92% (1%, min: 90%)

cg05202434 no cutting site

GCAGCCAATGCAACTCAACGGAAGTGACCTTCTCCTCCAAGCCACAGAGACTTGGCACCAGGCAACAATAACATCAATTGTGATGCTACCCAGGGCTCACAAAC
AGTCCTGGTGCCCTGAGACTAGTGCATGTGCATTCGTGAGGCAGGATCTGGTGCCAGGCTTCAGAGCTTTCAGCCTGCCTAAGCAGTGGGAAATGGTATGGGC
AGAGCCAGCCTGGCATAGGGCAAAAGGTGGCCTGTGAAAACCCACTATGGGACACTAAACTTCTCGACCTCAGGGCCCCTTCAGACCATTTACATGGTCGGGT
CTCACAGGAGGAGAAAATATTTTGAGACTCTGAGGTGGTCACTGGAAAAGCTTCTTCTGACTTCATTTCCAAAAAAGGCTGTGTGTGAGAATCTGGTTCCACAG
AGATTGGAATATAGTACCGTATTGGAATTGTTGTGTTGTTGAAGGTTCTTTGGGTGATAGAATCATACCTGAGACCACATGTGTGGATGTCAGTGAAAGATGGC
TGGGCTCTTGACCTCACCATCTCCATTCATCCTTGACATCACAGAGGCTTTCTGGGAAATGTAGGAGCCACGACGAAGGGAAG

cg05202434 Speichel 19% (21%), Blut 52% (7%), Sperma 87% (2%)

cg15781156 No cutting site HhaI

TTCTCTGAATAGAATTGTGTTTATAAAACATAGCTCATAAATCCTATTTGGCAAAAATAAGATATTCTTCAATATTTAGACATCTCTGCTGATTCATACAATAACCT
AAATTTCAGTCTTCCGAACTTCTGAGAACATAACCTCTGATGTTACAAATTCTGGTTTTAGGGTTCAACAAGAGTCTTCGTAGCAAGTATATTAACTCAAACATAT
GACTAGAGTAAAGGTGCCTGTGGTTTCATGAGAAATAACAGCTGTCTCGAAGAGCCAGAAAGCCTGGAAACTTTCTTTCCAGATCACAGATAGTTGACACTCAA
TCAGGGAAACGTTGAGTTCTGCATGCCTAAGAGGTTGTTGTCCAGGCTTGGGTCCAGATTGAGATCCAGGCATCAGGAGAGGTTGTTTTGTTAAAAGTGTGGC
TGGAGCAGCATGCAAGGATGAAAAAGAAGGAGAAAGAATGGACTCTTCAGTAAATGTTGAGGAAGATATTTTCTGAAGAGAATTAGCAGAAGAGGCTATGG
ATAGGACAATAGAAGAGGAGACCAGAGGAGAGGAGAAAAAAATGGAGCAAGGTGGGAAAATGCAGAATGAAGGGAATGTA

cg15781156 Speichel 18% (18%), Blut 44% (6%), Sperma 69% (2%)

Digestion controls

VK_GlaI cg15183843 ca. 3,5kb distance

Homo sapiens chromosome Y, GRCh38.p13 Primary Assembly Position: 12421150 to 12421449

CACGAACCCGCAGAACAGAGGCGCCGTCTTTGGGAAGCCGCGTGGATTTCCAGGCCTGGGGGCGACAGCGTGGCTGCCTCGACACCCTCGGCTGCAGGCCTC
GCCGGTGTCACCCCCGGCCTCCTACTCACTCCATGTCACCTGCGCCCCATGGGGCCCCGGCCTCCCCCGACGCCCCTGAGACCCCCACGCCCCGCAATTCCTCCT
CGGCGCTCTAGGTCAGCCTCGTGCCCTCTGGCCAGGGGCCCCTTGACCCCCTGTCCTCCCCACAGTCACCTGCGACCCCGCTGATTCTTAATACCCATGCGCCCA
GCAACGACACCGCACGCGGAACACCGCCCGACCTCAGCGGTGTCAGCAGCTGCCCCGTCGCCCAGCGCACTTGCTTGGAGTACTGGTACATCCTCTTCCCCCAA
CCCAGCTGCCCCCCGACCCGGTCACCCCTCTCCACTCCCTGGCCCACCCCTGCGCTCCCGGACCCCAGTATCTGCACCTCCTCCGAGCTTGAGCTGCCTCTGCCCG
TGTCCCCAGGCCAGGCGCCCCCTCCCCCGTCACTCCAGTGCCGCCCCACCCCAGCCCACTCCTGCGCCTCTCGCCGCCCCTCCTCCCCGTCCCGGCCTTCTGCCCA
TCCTCTCCAAGCGCCCCCGAATCCCCTCCACGCCCTGCACCTCTCCCTCCCCGCGCGCTCCCGCAGCCTCTGCCTTGGGCCTGCGCCTCCCTCCCCAAGCCTCTCA
GCTCTACGTCCCCGCCCCCGTCTGTCCTCCATACTGAGGCAGCCTCCGTCTCCCCGTGCCCACTGCGCCTGGACCACCTGTACTCCTCAGCACTCGGCGCCTCGG
CTCGGCCCACCCACCCGCAAGCGCCGCTAGCCTATCCCTGCGCCCCGCACCCCTCCCCGCCTTTGCTTTTGGGCAACCTCTCTCTGACCTCCAGCTCCCAGCGCCT
CTCGCCCCACCTCTGCACTCCAAGGCCGGCGCCCCTCCCCCAGCCCCCAGCCTCTGCCTCTGGGTTCCGCCCGCGCCCCGCTGTACGCCACCCACCCTCGAGACC
TCCTAGGCGTTCGGGCAGCCTCAGCGCCCCCCGGCCCCCAGCCGCGCCCAGGCCTCCTCCACTGCATACGATCCCCTGCACCCCGCTGCAGCCCCCGCACCTCTT
CCGGCGCCCTGGCTGCCTCTGCCCAAAGCCGGCGCTGGGGGCCCGGAGGGCAGCGATTGGCCTCTGTCCTGAAGCCCGGCCCCGCCTCCGCCCAGCCCTGGTA
GGCGCGGGTCAGCTCCTCAGCCCTGCAGCCCCTGCCCGCAAGCCCGCGCGCCTCCTGGTGTCCAGCCCCAGCTACCAAGGAGAGAGACCCAGGAACCGCCAGT
GGCTGCGCTCCACGCCGGCGCCCAGCCCCTCGCCCACCGCGGCCCCCTTGGCAGTTGGCGGTTTCTAGGTTTGGACATTTAACCTTTAATTGGCCGGCCCCAAA
GTAAATGTTTGCGATGACTTAAGGCCCTGGGCCCAAGGCCAGCGCGCAGCCCGAGTGGAGGCCGCTCTGGAAGCCTCCCAGAGGAGAAAGCAGAGCTCATAC
ACTATCTTGTTGAATGCATGAAAAATACACGTGCGCAAACTTGCACGCATGAATGAATGAATGAATGCACGAAAGAGAGAAAAAATGAGTGTTCAGGCCTCAG
CTGTTGAATA

VK_Gla_for_I Tm 66°C, 19bp
VK_Gla_rev_ITm 66°C, 19bp

VK IaIu I cg15183843

AAGCCCAGGAGAGAGGCCTCAGGAGGAACCAGCCCTGCCCACACTTACCTTGATCTCAAACCCAAAAGAGAATGCAGACAGGGCTGCATTTAGAGTGAGAGT
TTTATGGAACTACTGGACACCCTGTTTCTCTCTTTGGTTTAGAGTTGGGGGAATACTGACCACAGTCACACAATGACAAGACAACACCAGTTGGCACAAGAGCT
CTCCAGCAACAAGGTATCCGAGACTCAGACTTCTGCTTTGGAACAAAAATGTGCACCTCCTGAAGGTTAGCCACTTCCCATCCAGGACACTGTAAAACCCATCTA
AAGTGCAAAGACAGACCAGCGGATTGAAATAGAACAGATCATGAAACATTGATTGATAGGTGCTTAGCTTCCACATCACCTTGCCCTGAAGAAACAACTGGTG
ATCAAGTCGTGGTGCGGCGCCACAGAACATCCACAATTATCCAATGCTGCTGCATTCAAAGAGGGAGTTTCCACTTCATGTGCCAGTGCGGCAGACTTTCAGCA
AAGGCACGGAGTGGAAAAACAGCAAAAGAACTTGTCACTACACTACAGCCCACAAAAAGGAGACACTGTCACAAAATCAAGTCCAGCCTGGAGATTGCAGTG
GCTTAGAATTTTATGACTCTGTGACAACAGTTTCTTTCTACCCCTTGCCTGCCCCCCTGCACTCAAGGAATCTTCTGATCACCCCTCAGAATATCCTTAGCATCTTC
CTTCTCAGCCCTTCTCTACTCAAAGGCAGTTTCTGTTTTCTGCAAAGAGTTGAGTCTTCTCTTCTGGCATGAGG

cg15183843 (-vaginal, 96% Speichel, 85% Blut, 82% Sperma)

VK_IaIu_I for
GCTTCCACATCACCTTGCCC

VK_IaIu_I rev
CCGTGCCTTTGCTGAAAGTCT

VK_lalu_II **27254225** ca. 4kb distance

AGATCCTTTGTTCTGAGGCTCACTCATCTCCCGAGCCCCGAGCCGTCTCCCAGCCTCAGACGGCTCTGCGGGCTGCATCTGTGCAGCCTGGCAGCGGCGGCGCTGCGCTGTGACATTTTCACAGCCCTTCTTGCAGAGGCATGTGTGCTAGGGATGCCGAAATGCCGAGAGCGCCGGCAGGACTAGCTTCCGGGCCGCGCTTTGTGTGCTGGGCTGCAGTGTGGCGCGGGCGAGGAAGCTGGTAGGGCGGTTGTCGCAAGCTCCAGCTGCAGCCTCCGCCTACGTGAGAAGACTAGAAAGCGGGCGCAGGACCAGGCCTGCGTTGTTTGC

VK_lalu_II_forw 20bp 66Tm

VK_lalu_II_rev 19bp 66Tm

VK_lalu III cg17913570 ca. 1.7kb distance

GGGCCTGTCCACTTGACGGAAGCCCTGTAGTTAGAAGCGACAGCAGGGTCGTGCCTGGCGCTCTCCATGGGAATTGGGTGGGTGCAAAGGAAGTTATATAGGCTCAAGGCCTACACACCTTTGGGTGCAGCGCCCGCAGGGGGAAGAAAGCATATCTGGGGAGCTGG

VK_lalu_III_forw Tm 64°, 21bp

VK_lalu_III_rev Tm 64°, 21bp

VK lalu IV cg05999368 ca. 0.5kb distance

AGTGATCATTCAGGAGTACTGCCTTGGCATCCTCGGTAAGGAGCGCCTCCCAGCATGGTAGGGGAGCTGGTGTGTGGGAGGGTAGGTCTGGCATGAACCTTCCTGACTCCTCTCTCTGCAGGATACGGGATGTCTCATTCCACTGCAGTCTAGTGGTTGTGGGATCATGAAGGTCAAGCCTCCAGCTGCAGGCCGTACAGCTCCTACCTGACCTTCTTCAGCTGGTTGGCTGACCATGACTGCCCAGGTTCTGGCAGGATTGCTGAGGTGAGCGCCAGGTAGGGCATCATGGGA

More matches on Y Chromosome !!

VK_lalu_IV_fow Tm 64, 20bp

VK_lalu_IV_rev Tm 62, 19bp
